# Supplementary material for: Evolutionary trajectory of pattern recognition receptors in plants
Source: Nat Commun. 2024 Feb 1;15:308. doi: 10.1038/s41467-023-44408-3 (PMC10834447; doi:10.1038/s41467-023-44408-3)
Supplement: Supplementary file 1 — Supplementary Information file [file 41467_2023_44408_MOESM1_ESM.pdf]

Evolutionary Trajectory of Pattern Recognition Receptors in Plants

Bruno Pok Man Ngou[1], Michele Wyler[2], Marc W Schmid[2], Yasuhiro Kadota[1,#], Ken Shirasu[1,#]

[1]RIKEN Center for Sustainable Resource Science, Yokohama, Japan

[2]MWSchmid GmbH, 8750 Glarus, Switzerland

[#]Correspondence to: yasuhiro.kadota@riken.jp, ken.shirasu@riken.jp

Supplementary materials

Contains:

Supplementary Note 1

Supplementary Note 2

Supplementary figure 1-18

Supplementary References

Supplementary Note 1 :

For extended figure 2: *At*FLS2[1], *At*EFR[2], *Os*Xa21[3], *At*LORE[4], AtRDA2[5], AtWAK1/2[6,7], AtCRK13,28,38[8–10], AtDORN1[11], AtLecRK1.8[12], AtCERK1[13], AtFERONIA[14–18], AtCORK1[19], AtCLV1[20], AtHAESA[21], SRKs[22], OsDEES1[23], AtLecRK-IV.2[24], OsLecRK-S.7[25], AtBRI1[26], AtRGFRs[27,28], AtTMK1[29], OsSIK2[30], AtWAK4[31], AtCRK5,36[32,33], AtLecRK-A4 family (LecRKA4.1, LecRKA4.2, LecRKA4.3)[34], SlCf-4[35], AtRLP23[36], NbRXEG1[37], AtLYM1/3[38], OsLYP4/6[39], AtCLV2[40], AtTMM[41].

For extended figure 4: BAK1[42–45], SOBIR1[46], AtBIK1/AtPBL1[47,48], AtPBL19-20, 30-32, 34-40[49,50] AtCPK1,2,5,6,11,28[51–53], AtMEKK1, AtMAPKKK3/5, AtMKK4/5, AtMPK3/4/6[54], AtCNGC2,4,19,20[55–57], AtOSCA1.3, AtOSCA1.7[58], AtRbohD/F[59,60], AtEDS1[61], AtPAD4[62–64], AtSAG101[65], AtADR1[66], AtNRG1[67], AtSARD1, AtCBP60G[68], AtCPK2,11,20,24,33[69–71], AtCNGC8,16,18[72–74], AtRbohH, AtRbohJ[75], AtCPK11,12,28,30,32[76–78], AtCNGC5,6,9[79], AtRbohC[80].

For extended figure 5: AtRLP1[81], AtRLP23[36], AtRLP30[82], AtRLP32[83], AtRLP42[84], NbCSPR[85], NbRXEG1[37], SlCf-2/4/5/9[35,86–90], SlEIX2[91], SlVe1[92,93], SlI[94], SmELR[95,96], BnRLM2[97], SlCuRe1[98], VnINR[99], NbREL[100], AtCLV2[40], AtTMM[41].

For reference in supplementary figure legends: [101–118].

Supplementary Note 2

The expansion rate of cell-surface receptors and signalling components

The expansion rate of cell-surface receptors and signalling components in this study is determined by calculating the number of identified genes divided by the number searched genes, or the number of annotated proteins in each genome. We have tried to repeat this analysis while implementing evolutionary models and housekeeping genes. We originally tried to reconstruct a species phylogenetic tree with tools like Orthofinder2, but that simply does not find any genes that could be used to reconstruct the species tree. We have also searched through the 350 genomes for single copy BUSCO genes and not a single one was found in all species. The maximum was a single gene in 300 species. Most of the BUSCO genes are found in about 250-270 species (see Peer Review file). As a result, we cannot improve our gene family expansion analysis in this study. We believe that more tailored analysis are required to study the expansion/contract rate of each cell-surface receptor classess in the future.

Further discussions

The study of PRRs and the core PTI-signalling pathway has primarily focused on model plants, such as Arabidopsis and rice. However, recent investigations into chitin perception by LysM-RLKs in the Bryophyte *Marchantia polymorpha* have revealed a high degree of conservation in the PTI-signalling pathway across land plants[134,135]. In fact, most cell-surface receptors and PTI signalling components in the Tracheophytes are conserved in Bryophytes, with the exception of Duf26-RLKs, EP proteins, and RPW8-NLRs (Main figure 6a). Therefore, the most recent common ancestor of land plants is likely to possess a considerable number of cell-surface receptors and basic components of immune signalling network, facilitating adaptation to terrestrial environments (Main figure 6a). The presence of diverse cell-surface receptor classes and PTI signalling components in algal species suggests that algae may also have PTI system (Main figure 6a), as indicated by the MAMPs triggering of defense responses in some algal species. For instance, treatment with lipopolysaccharides (LPS) and oligosaccharides in multiple red algae species (Rhodophyta) stimulated ROS production, activation of nitric oxide (NO) signalling, changes in protein expression and hypersensitive responses (HR)[136]. Land plants perceive LPS and oligosaccharides through G-lectin- and WAK-/LysM-receptors[4,6]. In Rhodophyta, we identified the presence of LysM-RLPs, but not WAK- or G-lectin-containing cell-surface receptors, suggesting that other PRRs perceive LPS in algae. Nevertheless, PTI is likely to be present in multiple algal lineages. Further investigation into PRRs and signalling network in the Glaucophyta, Rhodophyta, and green algal species holds promise for shedding light on the evolution PTI in Viridiplantae.

a

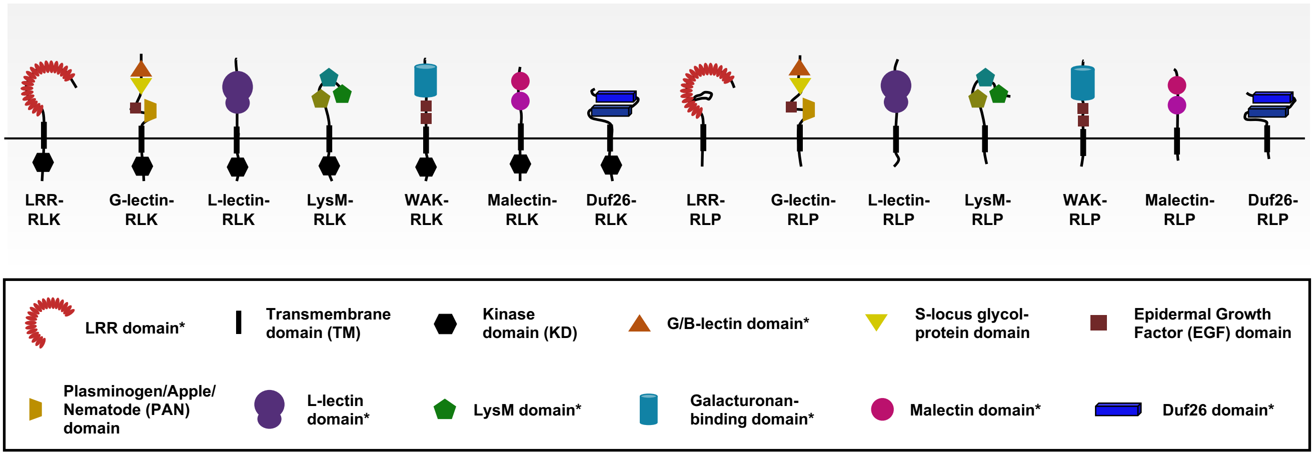

b

Type of receptor		Immunity-related		Reproduction-related		Development-related	
**Name**	**Icon**	**Example**	**L**	**Example**	**L**	**Example**	**L**
**LRR-RLK**		*At*FLS2, *At*EFR, *Os*Xa21		*At*CLV1, *At*HAESA		*At*BRI1, *At*RGFRs, *At*TMK1	
**G-lectin-RLK**		*At*LORE, *At*RDA2		SRKs involved in self-incompatibility		*Os*SIK2	?
**WAK-RLK**		*At*WAK1/2		*Os*DEES1	?	AtWAK4	?
**Duf26-RLK**		*At*CRK13, 28, 36	?			AtCRK5, 36	?
**L-lectin-RLK**		*At*DORN1, *At*LecRK1.8		*At*LecRK-IV.2, *Os*LecRK-S.7	?	*At*LecRK-A4 family	?
**LysM-RLK**		*At*CERK1, multiple LYKs					
**Malectin-RLK**		*At*FERONIA, AtCORK1		*At*FERONIA		*At*FERONIA	

c

Type of receptor		Immunity-related		Reproduction-related		Development-related	
**Name**	**Icon**	**Example**	**L**	**Example**	**L**	**Example**	**L**
**LRR-RLP**		*Sl*Cf-4, *At*RLP23, *Nb*RXEG1		*At*CLV2		*At*CLV2, *At*TMM	
**G-lectin-RLP**							
**WAK-RLP**							
**Duf26-RLP**							
**L-lectin-RLP**							
**LysM-RLP**		*At*LYM1/3, *Os*LYP4/6					
**Malectin-RLP**							

Pathogen-associated molecular patterns (PAMPs) | **Damage-associated molecular patterns (DAMPs)** | **Phytohormones, Self-proteins/peptides/phytocytokines**

Supplementary figure 1. Domain architecture and roles of the major cell-surface receptors classes in plants. (a) Schematic figure represents the domain architecture of different classes of receptor-like kinase (RLKs) and receptor-like proteins (RLP) in plants. Lower box defines the domains in the receptor classes. '*' represents the definitive domains in these receptors. Note that the number of definitive domains and the domain architecture in each receptor is variable. **(b-c)** Table representing the characterized (b) RLKs and (c) RLPs in plants; the biological processes of which the characterized members are involved in, and type of ligand (L) of which they perceive. Grey box indicate indicates that receptor class has not been reported to involved in that biological process. For ligands, red box indicates PAMPs from pathogens, purple box indicates DAMPs released from damage and blue box indicates phytohormones, self-protein/peptide or phytocytokines, question mark (?) indicates unidentified ligand. Abbreviations for plant species: *A. thaliana*, *At*; *S. lycopersicum*, *Sl*; *O. sativa*, *Os*; *N. benthamiana*, *Nb*. References to the genes are included in the supplementary information.

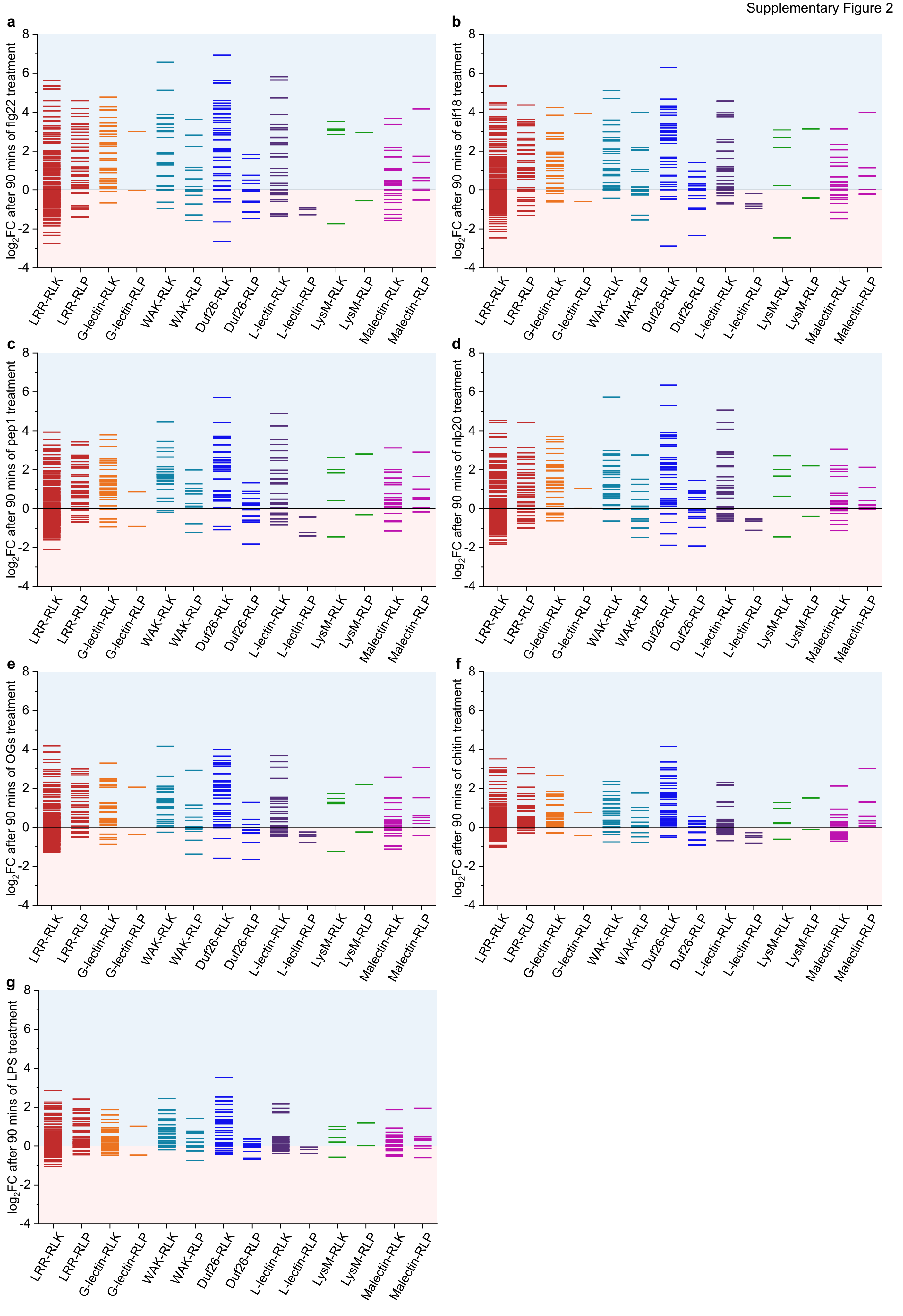
a
b
c
d
e
f
g
log2FC after 90 mins of flg22 treatment
log2FC after 90 mins of elf18 treatment
log2FC after 90 mins of pep1 treatment
log2FC after 90 mins of nlp20 treatment
log2FC after 90 mins of OGs treatment
log2FC after 90 mins of chitin treatment
log2FC after 90 mins of LPS treatment
8
6
4
2
0
-2
-4
LRR-RLK
LRR-RLP
G-lectin-RLK
G-lectin-RLP
WAK-RLK
WAK-RLP
Duf26-RLK
Duf26-RLP
L-lectin-RLK
L-lectin-RLP
LysM-RLK
LysM-RLP
Malectin-RLK
Malectin-RLP

Supplementary figure 2. Expression of cell-surface receptors in *Arabidopsis thaliana* during PTI. *A. thaliana* seedlings were treated with **(a)** flg22, **(b)** elf18, **(c)** pep1, **(d)** nlp20, **(e)** OGs, **(f)** chitin, or **(g)** LPS to activate PTI. Light blue represents increased expression and light pink represents decreased expression during PTI. X-axis values represent log2 (fold change during PTI relative to samples at 0 min after PAMP/DAMP treatment). RNA-seq data analyzed here were reported previously, where PTI was activated by different PAMPs/DAMPs in *A. thaliana* for 90mins. RNA-seq data were obtained from Bjornson et al, *Nature Plant*s 2021 (reference 15 in main text).). For a-g, n (number of cell-surface receptors analyzed in the RNAseq data) is provided in the Source Data file.

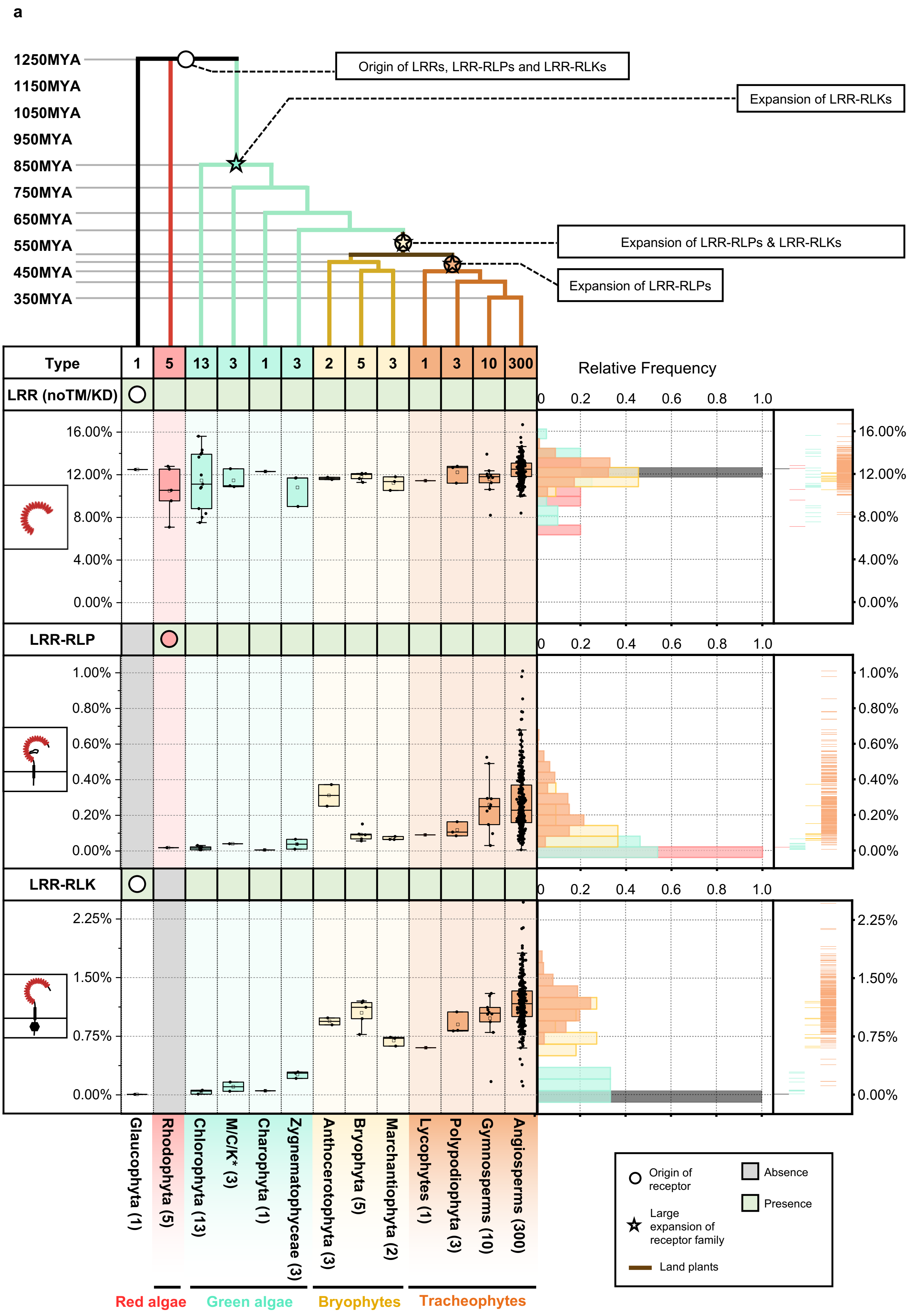

a
1250MYA
1150MYA
1050MYA
950MYA
850MYA
750MYA
650MYA
550MYA
450MYA
350MYA
Origin of LRRs, LRR-RLPs and LRR-RLKs
Expansion of LRR-RLKs
Expansion of LRR-RLPs & LRR-RLKs
Expansion of LRR-RLPs
Type
1
5
13
3
1
3
2
5
3
1
3
10
300
LRR (noTM/KD)
LRR-RLP
LRR-RLK
Relative Frequency
0
0.2
0.4
0.6
0.8
1.0
16.00%
12.00%
8.00%
4.00%
0.00%
1.00%
0.80%
0.60%
0.40%
0.20%
0.00%
2.25%
1.50%
0.75%
0.00%
Glaucophyta (1)
Rhodophyta (5)
Chlorophyta (13)
M/C/K* (3)
Charophyta (1)
Zygnematophyceae (3)
Anthocerotophyta (3)
Bryophyta (5)
Marchantiophyta (2)
Lycophytes (1)
Polypodiophyta (3)
Gymnosperms (10)
Angiosperms (300)
Red algae
Green algae
Bryophytes
Tracheophytes
Origin of receptor
Large expansion of receptor family
Land plants
Absence
Presence

a (continued)

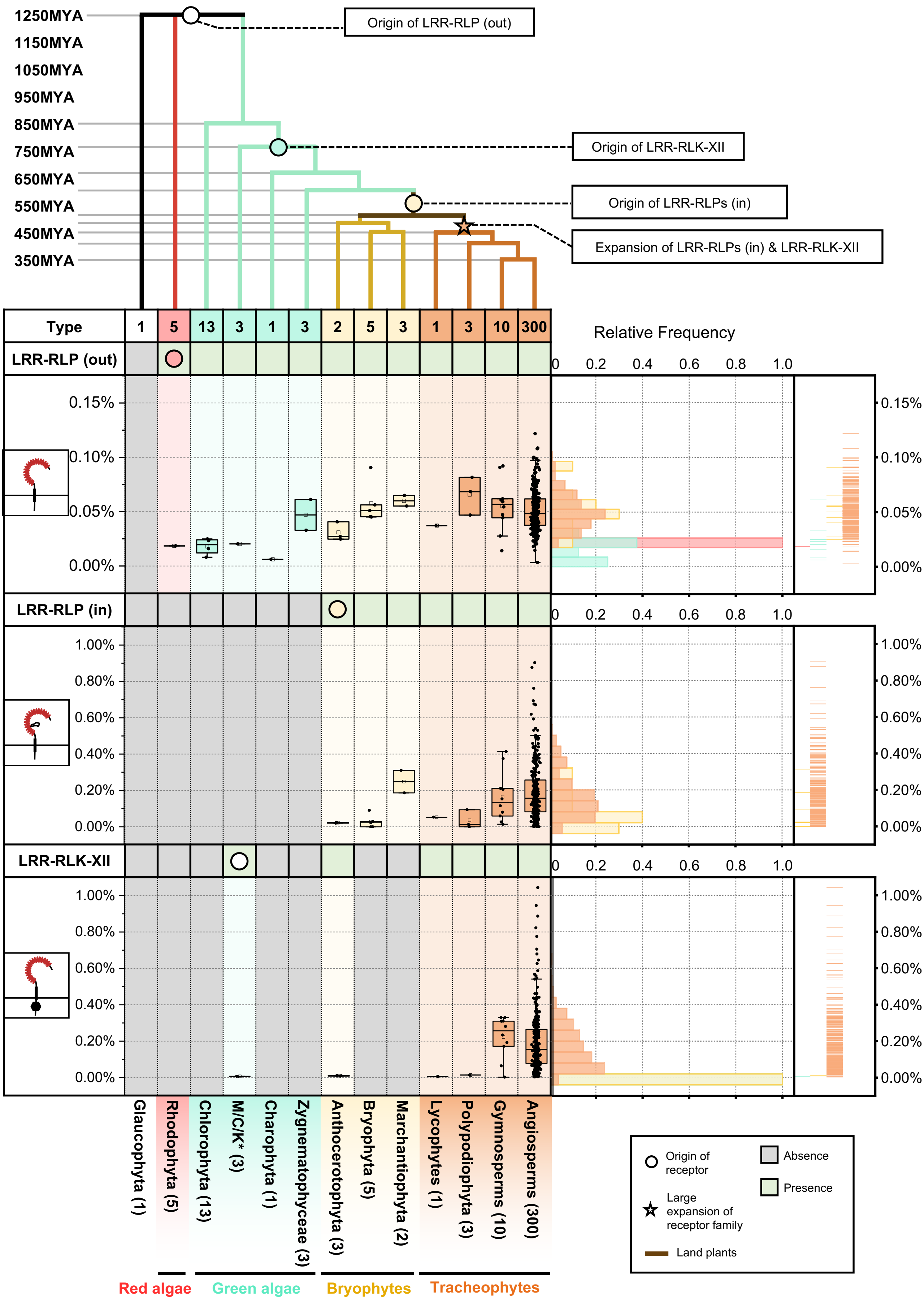
1250MYA
1150MYA
1050MYA
950MYA
850MYA
750MYA
650MYA
550MYA
450MYA
350MYA
Origin of LRR-RLP (out)
Origin of LRR-RLK-XII
Origin of LRR-RLPs (in)
Expansion of LRR-RLPs (in) & LRR-RLK-XII
Type
1
5
13
3
1
3
2
5
3
1
3
10
300
LRR-RLP (out)
LRR-RLP (in)
LRR-RLK-XII
Relative Frequency
0
0.2
0.4
0.6
0.8
1.0
0.15%
0.10%
0.05%
0.00%
1.00%
0.80%
0.60%
0.40%
0.20%
Glaucophyta (1)
Rhodophyta (5)
Chlorophyta (13)
M/C/K* (3)
Charophyta (1)
Zygnematophyceae (3)
Anthocerotophyta (3)
Bryophyta (5)
Marchantiophyta (2)
Lycophytes (1)
Polypodiophyta (3)
Gymnosperms (10)
Angiosperms (300)
Red algae
Green algae
Bryophytes
Tracheophytes
Origin of receptor
Large expansion of receptor family
Land plants
Absence
Presence

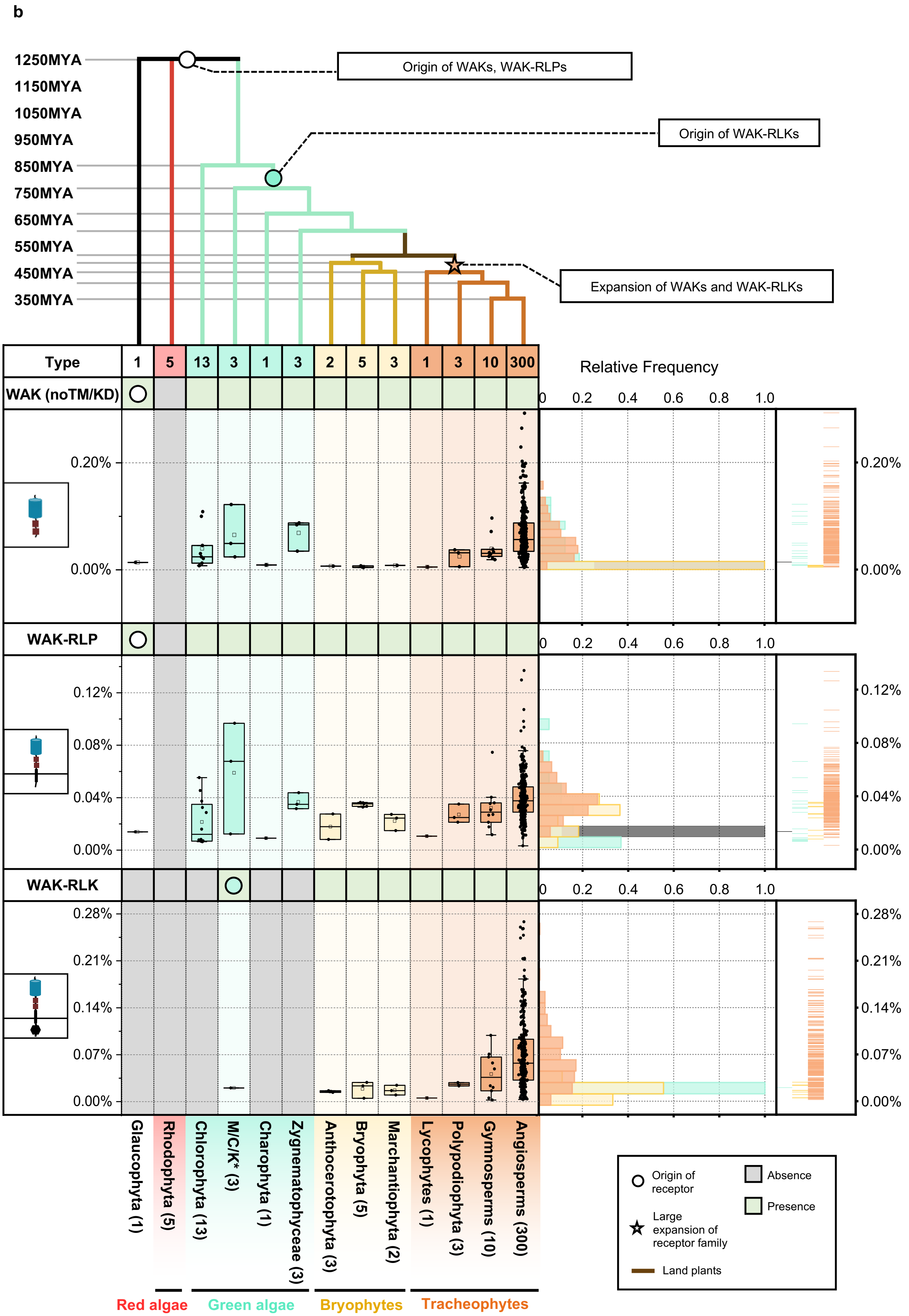
b
1250MYA
1150MYA
1050MYA
950MYA
850MYA
750MYA
650MYA
550MYA
450MYA
350MYA
Origin of WAKs, WAK-RLPs
Origin of WAK-RLKs
Expansion of WAKs and WAK-RLKs
Type
1 5 13 3 1 3 2 5 3 1 3 10 300
WAK (noTM/KD)
WAK-RLP
WAK-RLK
Relative Frequency
0 0.2 0.4 0.6 0.8 1.0
0.20%
0.00%
0.12%
0.08%
0.04%
0.28%
0.21%
0.14%
0.07%
Glaucophyta (1)
Rhodophyta (5)
Chlorophyta (13)
M/C/K* (3)
Charophyta (1)
Zygnematophyceae (3)
Anthocerotophyta (3)
Bryophyta (5)
Marchantiophyta (2)
Lycophytes (1)
Polypodiophyta (3)
Gymnosperms (10)
Angiosperms (300)
Red algae
Green algae
Bryophytes
Tracheophytes
Origin of receptor
Large expansion of receptor family
Land plants
Absence
Presence

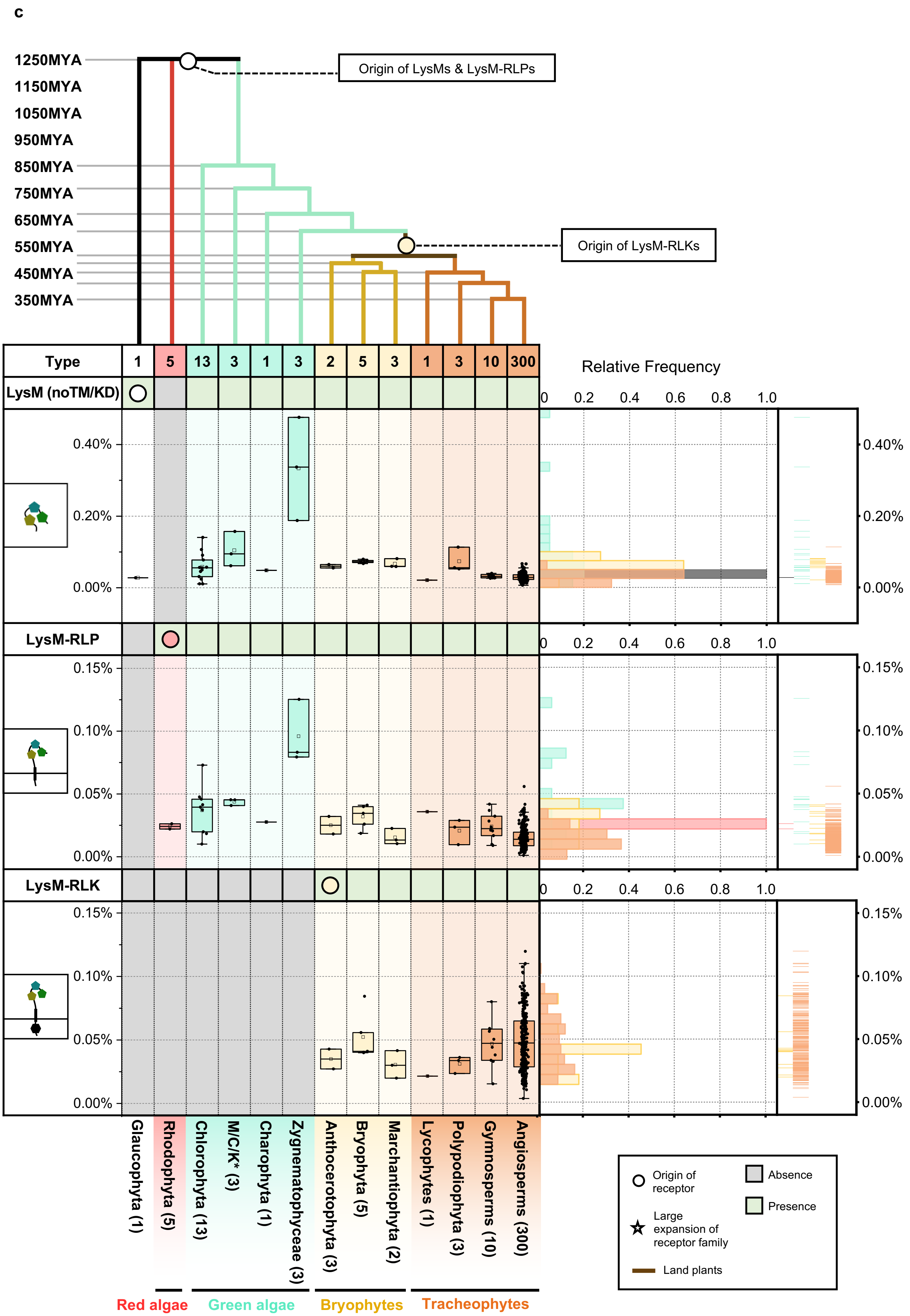
c
1250MYA
1150MYA
1050MYA
950MYA
850MYA
750MYA
650MYA
550MYA
450MYA
350MYA
Origin of LysMs & LysM-RLPs
Origin of LysM-RLKs
Type
1
5
13
3
1
3
2
5
3
1
3
10
300
LysM (noTM/KD)
LysM-RLP
LysM-RLK
0.40%
0.20%
0.00%
0.15%
0.10%
0.05%
Relative Frequency
0
0.2
0.4
0.6
0.8
1.0
Glaucophyta (1)
Rhodophyta (5)
Chlorophyta (13)
M/C/K* (3)
Charophyta (1)
Zygnematophyceae (3)
Anthocerotophyta (3)
Bryophyta (5)
Marchantiophyta (2)
Lycophytes (1)
Polypodiophyta (3)
Gymnosperms (10)
Angiosperms (300)
Red algae
Green algae
Bryophytes
Tracheophytes
Origin of receptor
Large expansion of receptor family
Land plants
Absence
Presence

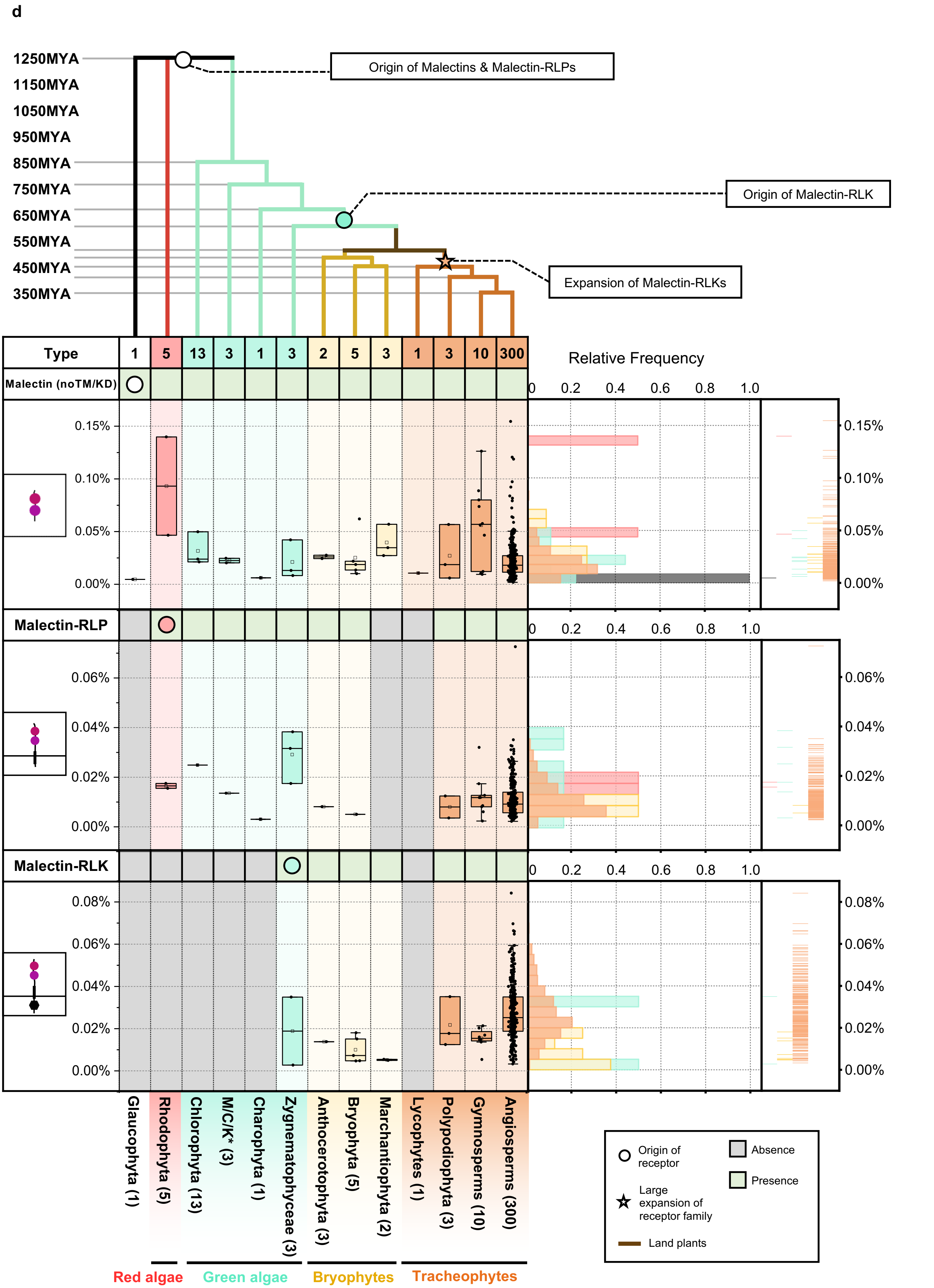
d
1250MYA
1150MYA
1050MYA
950MYA
850MYA
750MYA
650MYA
550MYA
450MYA
350MYA
Origin of Malectins & Malectin-RLPs
Origin of Malectin-RLK
Expansion of Malectin-RLKs
Type
1 5 13 3 1 3 2 5 3 1 3 10 300
Malectin (noTM/KD)
Malectin-RLP
Malectin-RLK
Relative Frequency
0 0.2 0.4 0.6 0.8 1.0
0.15%
0.10%
0.05%
0.00%
0.06%
0.04%
0.02%
0.08%
Glaucophyta (1)
Rhodophyta (5)
Chlorophyta (13)
M/C/K* (3)
Charophyta (1)
Zygnematophyceae (3)
Anthocerotophyta (3)
Bryophyta (5)
Marchantiophyta (2)
Lycophytes (1)
Polypodiophyta (3)
Gymnosperms (10)
Angiosperms (300)
Red algae
Green algae
Bryophytes
Tracheophytes
Origin of receptor
Large expansion of receptor family
Land plants
Absence
Presence

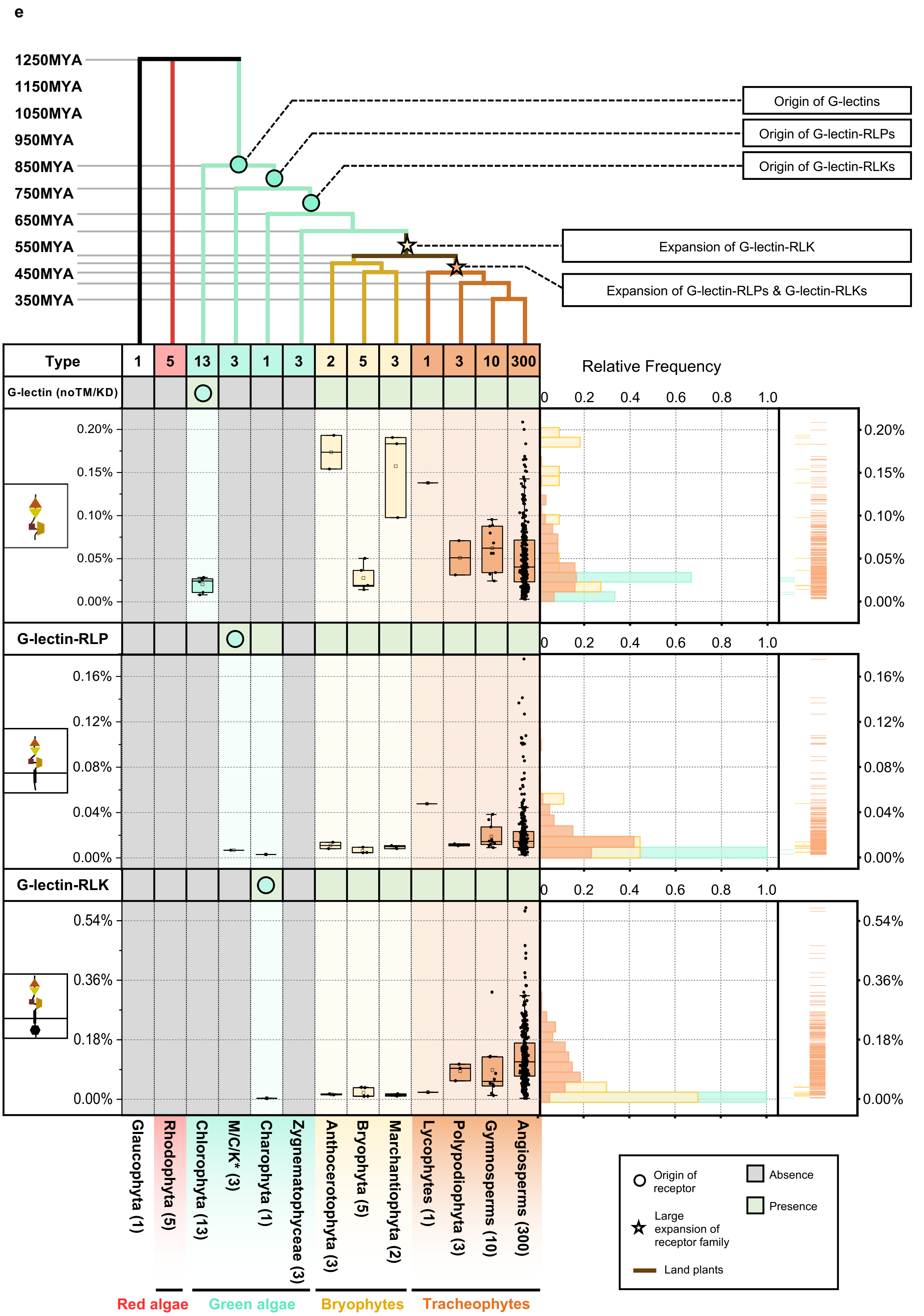
e
1250MYA
1150MYA
1050MYA
950MYA
850MYA
750MYA
650MYA
550MYA
450MYA
350MYA
Origin of G-lectins
Origin of G-lectin-RLPs
Origin of G-lectin-RLKs
Expansion of G-lectin-RLK
Expansion of G-lectin-RLPs & G-lectin-RLKs
Type
1
5
13
3
1
3
2
5
3
1
3
10
300
G-lectin (noTM/KD)
G-lectin-RLP
G-lectin-RLK
Relative Frequency
0
0.2
0.4
0.6
0.8
1.0
0.20%
0.15%
0.10%
0.05%
0.00%
0.16%
0.12%
0.08%
0.04%
0.54%
0.36%
0.18%
Glaucophyta (1)
Rhodophyta (5)
Chlorophyta (13)
M/C/K* (3)
Charophyta (1)
Zygnematophyceae (3)
Anthocerotophyta (3)
Bryophyta (5)
Marchantiophyta (2)
Lycophytes (1)
Polypodiophyta (3)
Gymnosperms (10)
Angiosperms (300)
Red algae
Green algae
Bryophytes
Tracheophytes
Origin of receptor
Large expansion of receptor family
Land plants
Absence
Presence

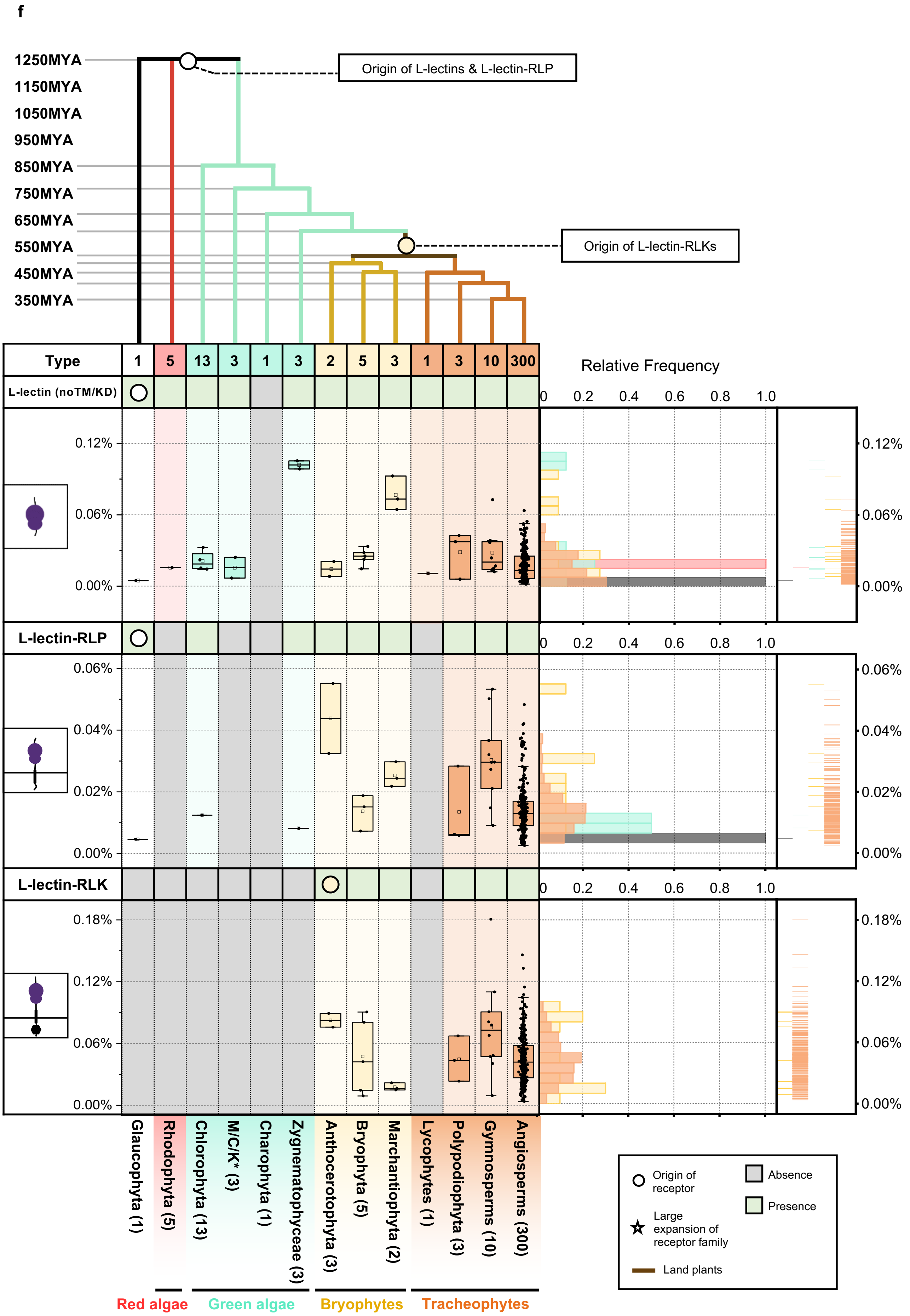
f
1250MYA
1150MYA
1050MYA
950MYA
850MYA
750MYA
650MYA
550MYA
450MYA
350MYA
Origin of L-lectins & L-lectin-RLP
Origin of L-lectin-RLKs
Type
1
5
13
3
1
3
2
5
3
1
3
10
300
L-lectin (noTM/KD)
L-lectin-RLP
L-lectin-RLK
Relative Frequency
0
0.2
0.4
0.6
0.8
1.0
0.12%
0.06%
0.00%
0.04%
0.02%
0.18%
Glaucophyta (1)
Rhodophyta (5)
Chlorophyta (13)
M/C/K* (3)
Charophyta (1)
Zygnematophyceae (3)
Anthocerotophyta (3)
Bryophyta (5)
Marchantiophyta (2)
Lycophytes (1)
Polypodiophyta (3)
Gymnosperms (10)
Angiosperms (300)
Red algae
Green algae
Bryophytes
Tracheophytes
Origin of receptor
Large expansion of receptor family
Land plants
Absence
Presence

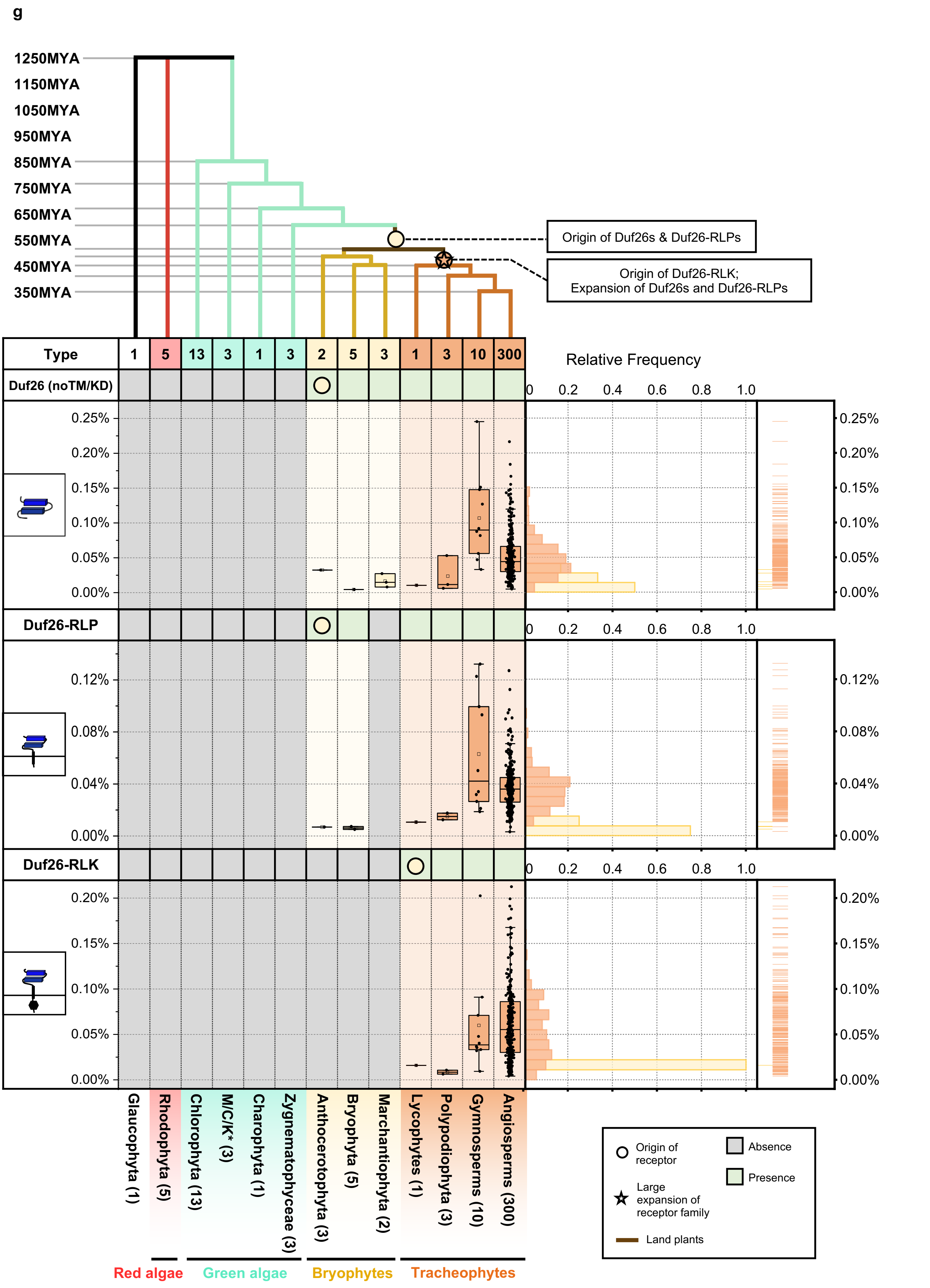
g
1250MYA
1150MYA
1050MYA
950MYA
850MYA
750MYA
650MYA
550MYA
450MYA
350MYA
Origin of Duf26s & Duf26-RLPs
Origin of Duf26-RLK;
Expansion of Duf26s and Duf26-RLPs
Type
1
5
13
3
1
3
2
5
3
1
3
10
300
Duf26 (noTM/KD)
Duf26-RLP
Duf26-RLK
Relative Frequency
0
0.2
0.4
0.6
0.8
1.0
0.25%
0.20%
0.15%
0.10%
0.05%
0.00%
0.12%
0.08%
0.04%
0.00%
Glaucophyta (1)
Rhodophyta (5)
Chlorophyta (13)
M/C/K* (3)
Charophyta (1)
Zygnematophyceae (3)
Anthocerotophyta (3)
Bryophyta (5)
Marchantiophyta (2)
Lycophytes (1)
Polypodiophyta (3)
Gymnosperms (10)
Angiosperms (300)
Red algae
Green algae
Bryophytes
Tracheophytes
Origin of receptor
Large expansion of receptor family
Land plants
Absence
Presence

Supplementary figure 3. The origin and expansion of cell-surface receptors in Viridiplantae. The origin and expansion of **(a)** LRR-, **(a continued)** LRR-RLP subgroups and LRR-RLK-XII, **(b)** WAK-, **(c)** LysM-, **(d)** Malectin, **(e)** G-lectin, **(f)** L-lectin, and **(g)** Duf26-domains in plants. Top panel represents a sequence similarity tree of multiple algal and plant lineages. Circles (○) and stars (☆) indicate the origin and expansion of receptor families. The timescale (in million years; MYA) of the sequence similarity tree is estimated by TIMETREE. Bottom panel represents the present/absence of an ectodomain (with no TM/KD), ectodomain-RLP, ectodomain-RLK in different algal and plant lineages. Grey box indicates the absence of receptor and green box indicates the presence of receptors in each lineage. *M/C/K represents Mesostigmatophyceae, Chlorokybophyceae and Klebsormidiophyceae. Number of available species from algae and plant lineages are indicated by numbers in the boxes. Boxplot below represents the percentage (%) of ectodomains (in proteins without TM or KD), ectodomain-RLPs and ectodomain-RLKs in each lineage. Right plot represents the distribution of the relative frequency of the percentage of ectodomains, ectodomain-RLPs and ectodomain-RLKs in each lineage. Boxplot elements: centre line, median; bounds of box, 25th and 75th percentiles; whiskers, 1.5 × IQR from 25th and 75th percentiles. For **a-g**, n (number of cell-surface receptors analyzed) is provided in the 'Protein counts per species' file on *Zenodo* (see Data availability section).

a

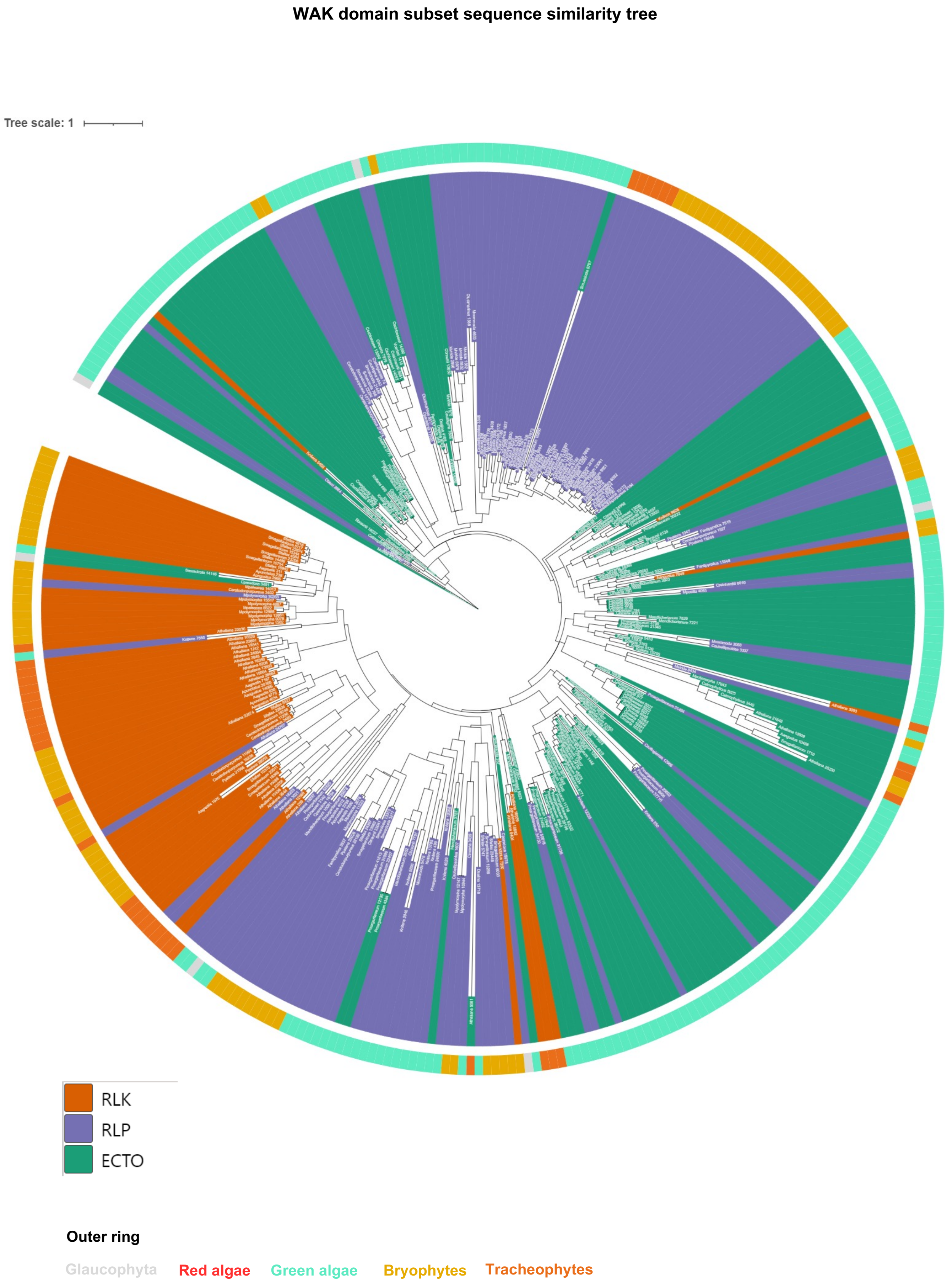
WAK domain subset sequence similarity tree
Tree scale: 1
RLK
RLP
ECTO
Outer ring
Glaucophyta
Red algae
Green algae
Bryophytes
Tracheophytes

b

WAK domain full sequence similarity tree

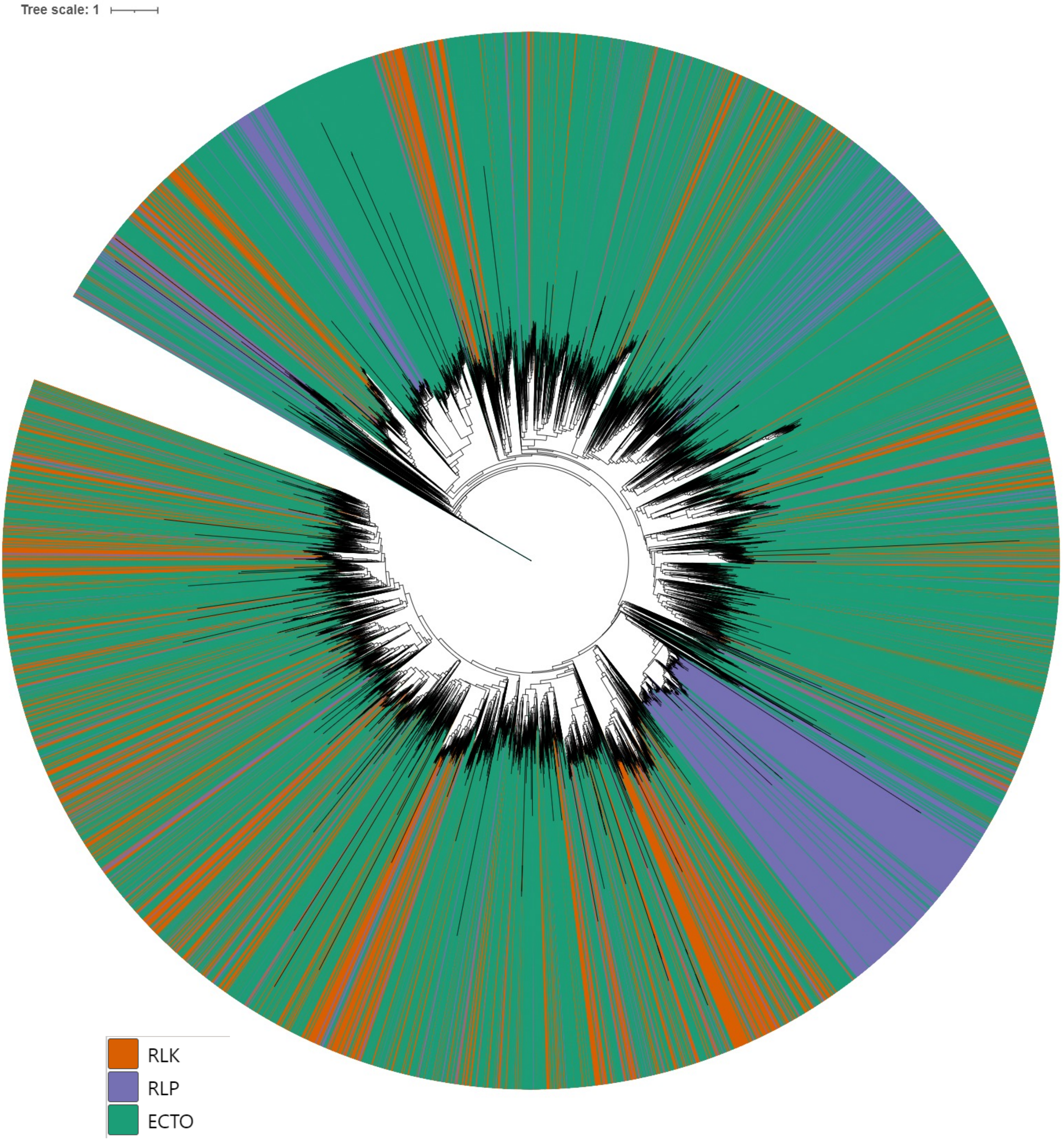

c

LysM domain subset sequence similarity tree

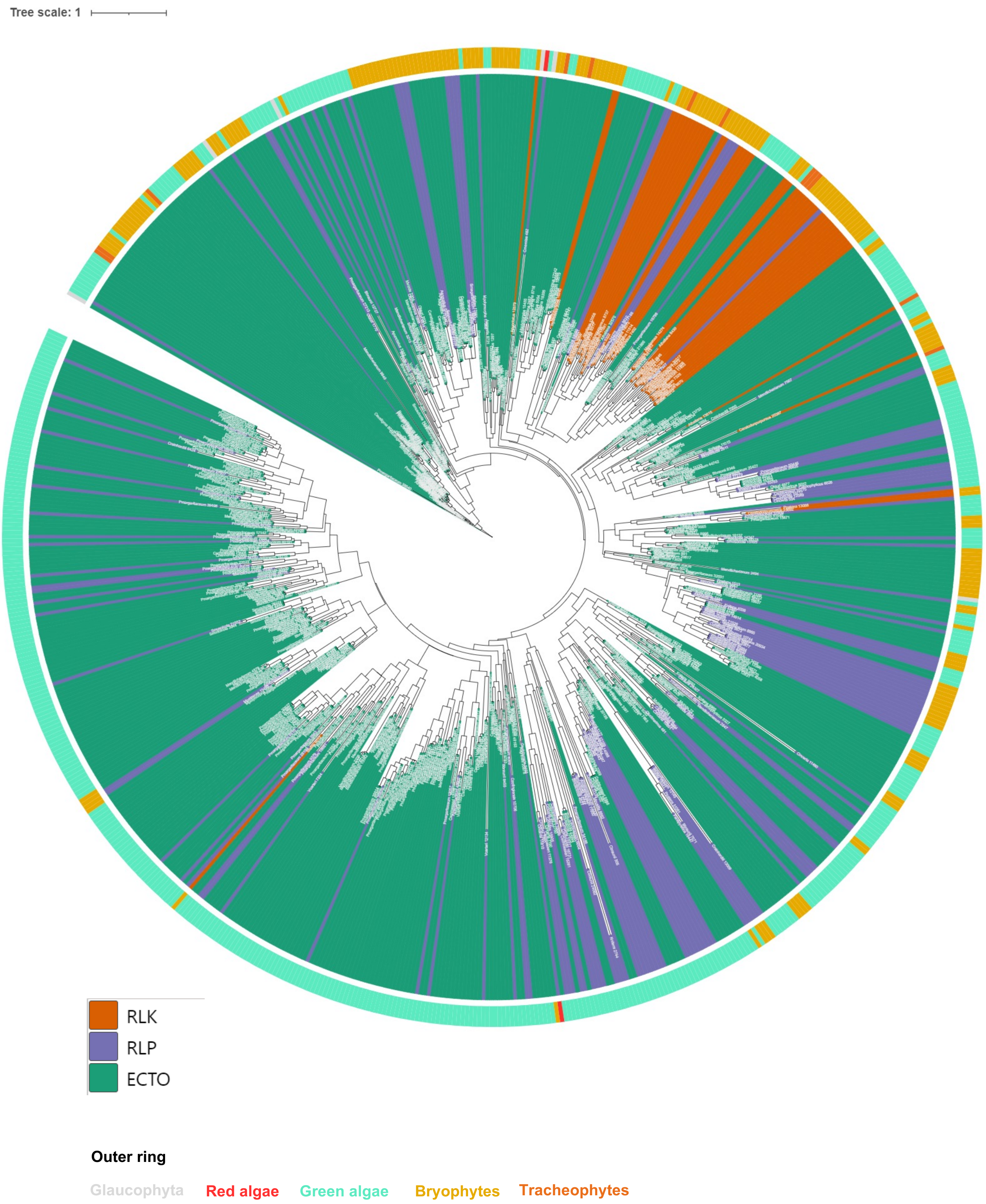

d

LysM domain full sequence similarity tree

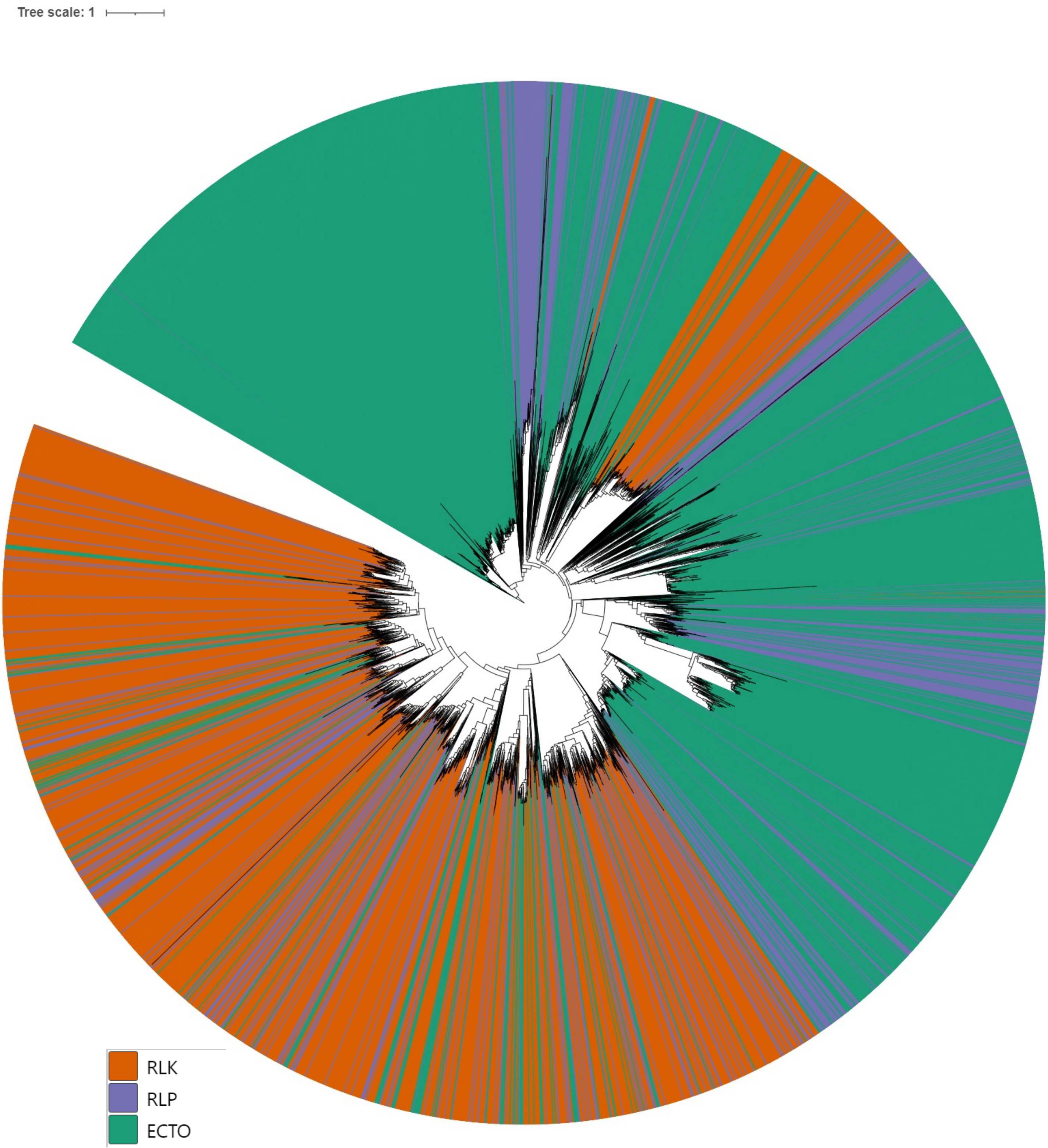

e

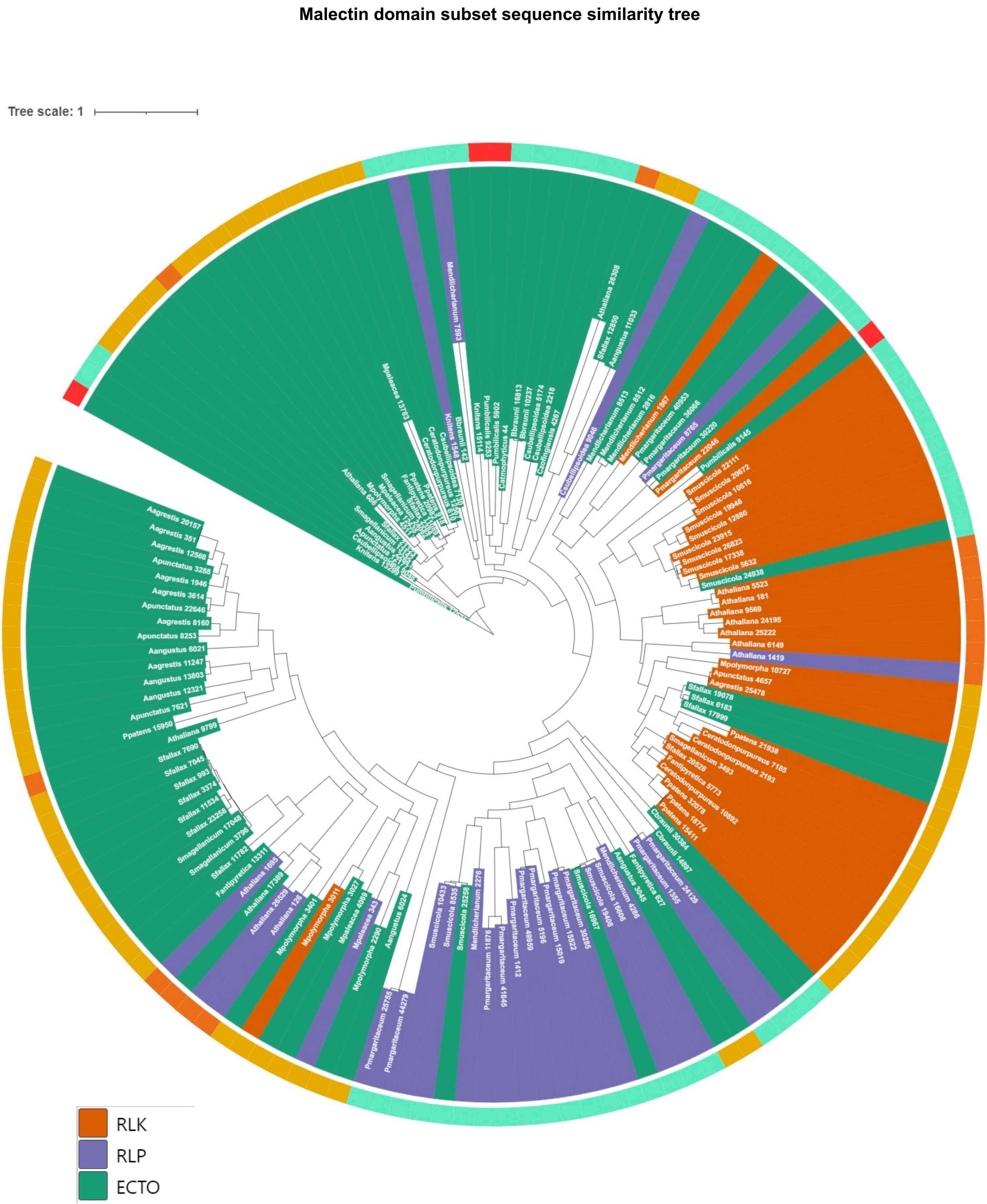

Outer ring

Glaucophyta Red algae Green algae Bryophytes Tracheophytes

f

Malectin domain full sequence similarity tree

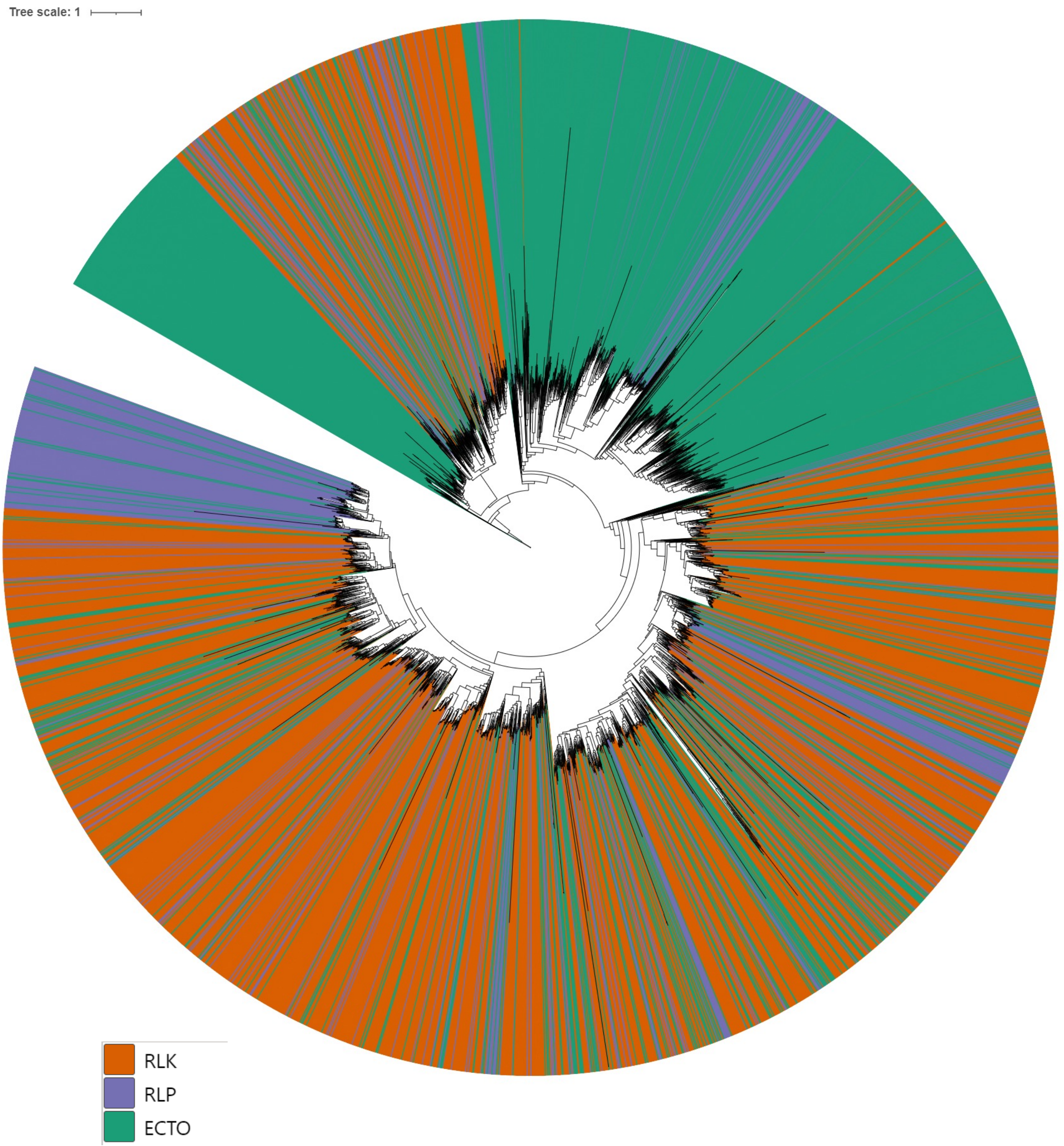

g

G-lectin domain subset sequence similarity tree

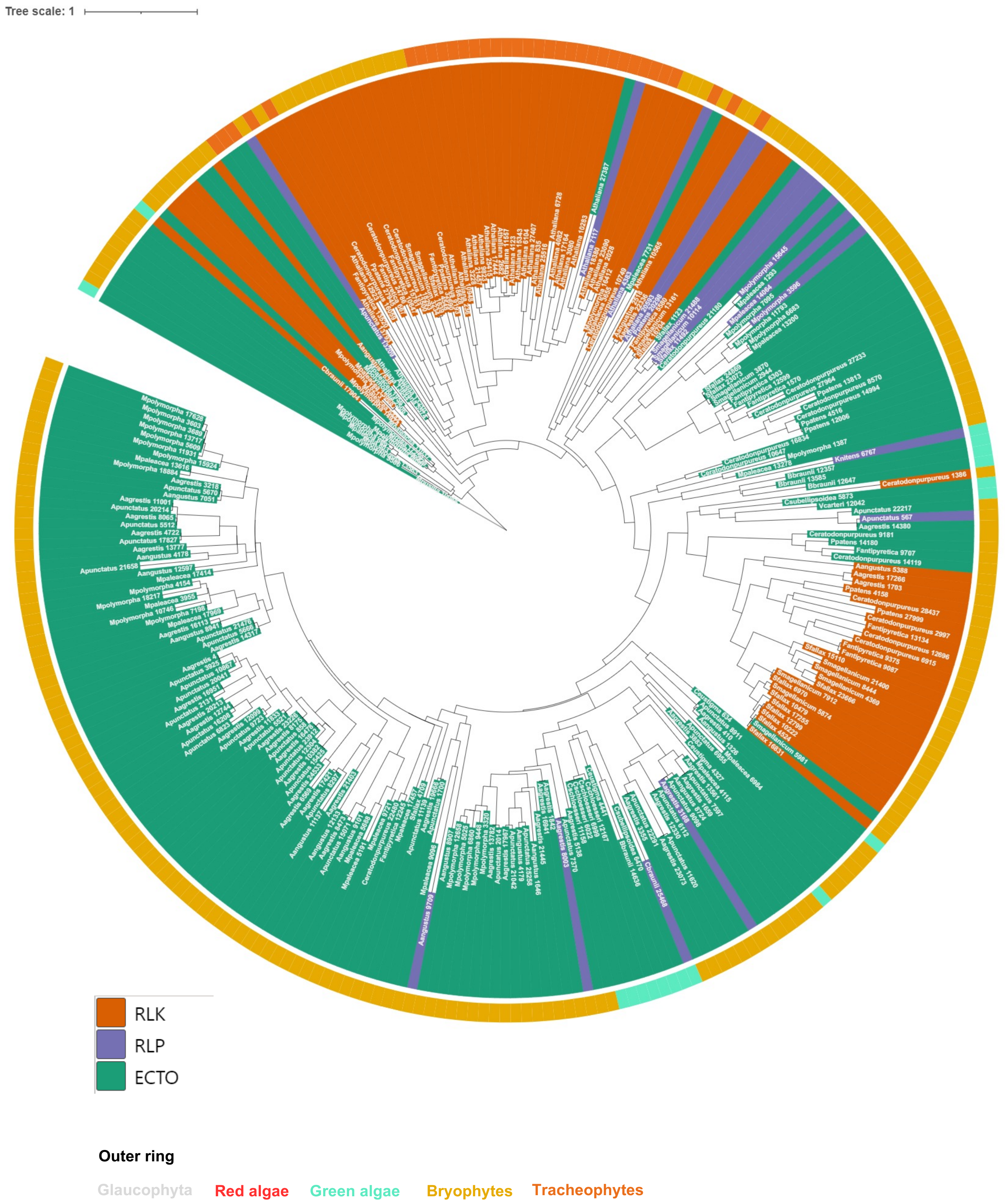

h

G-lectin domain full sequence similarity tree

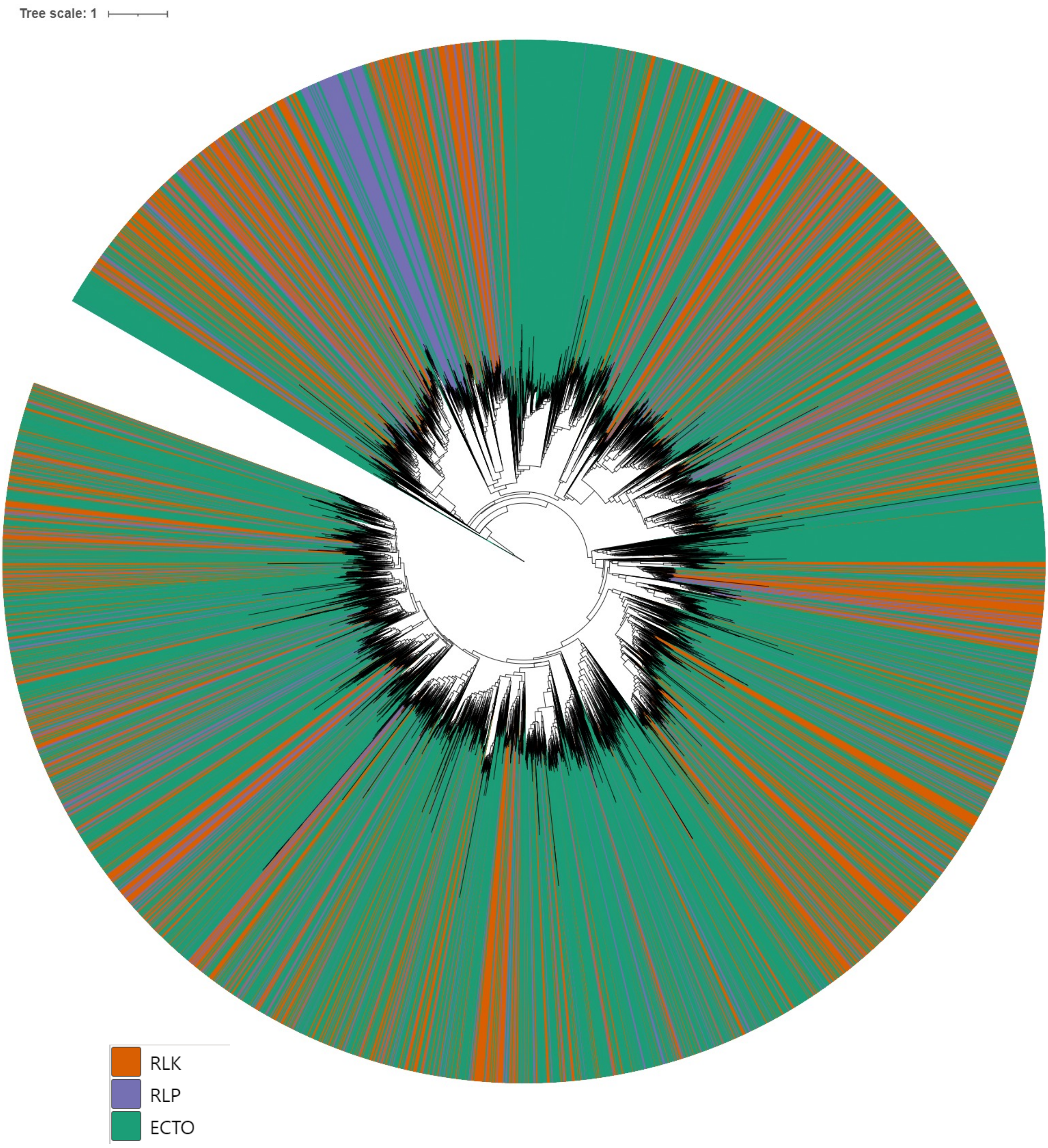

i

L-lectin domain subset sequence similarity tree

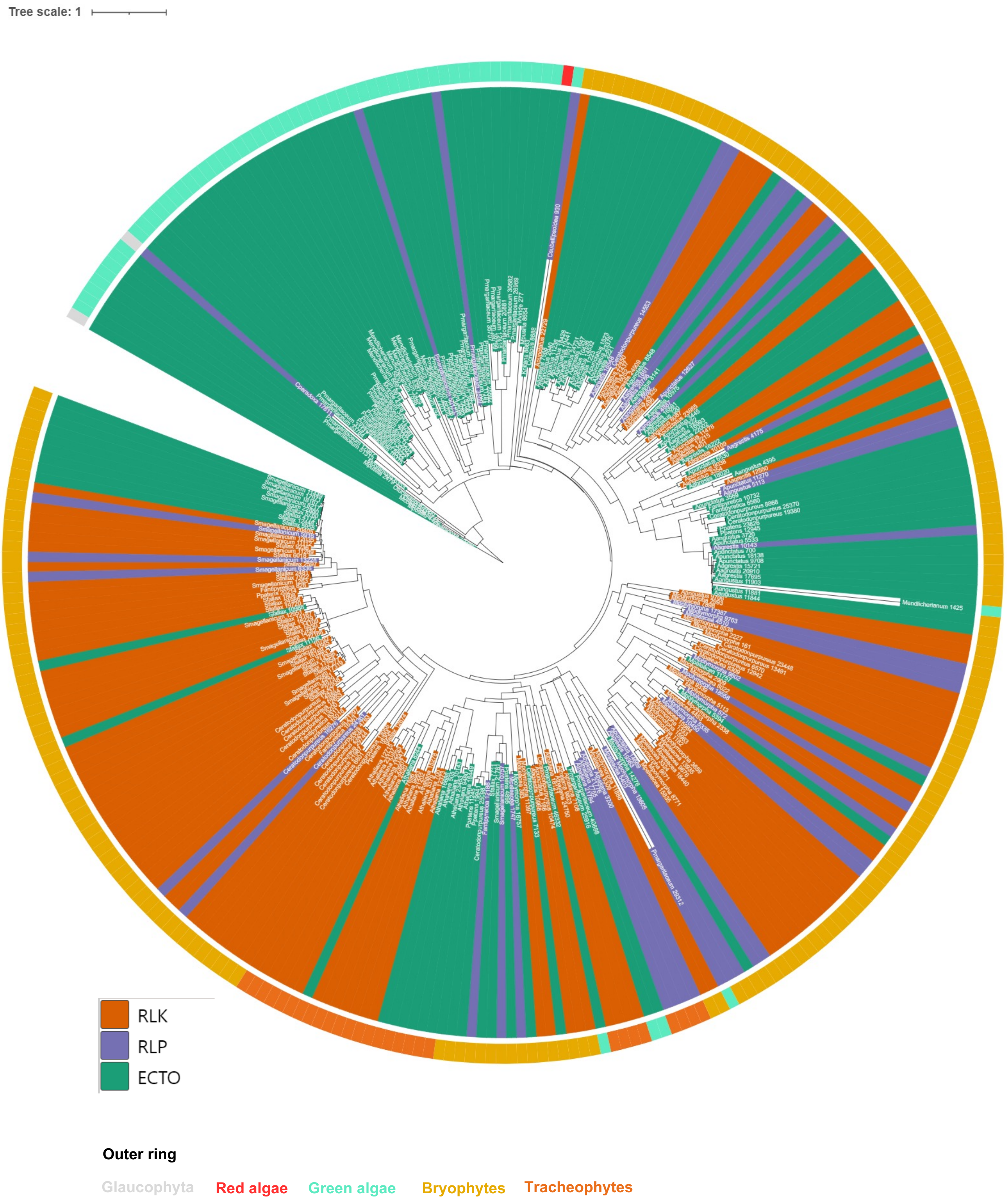

j

L-lectin domain full sequence similarity tree

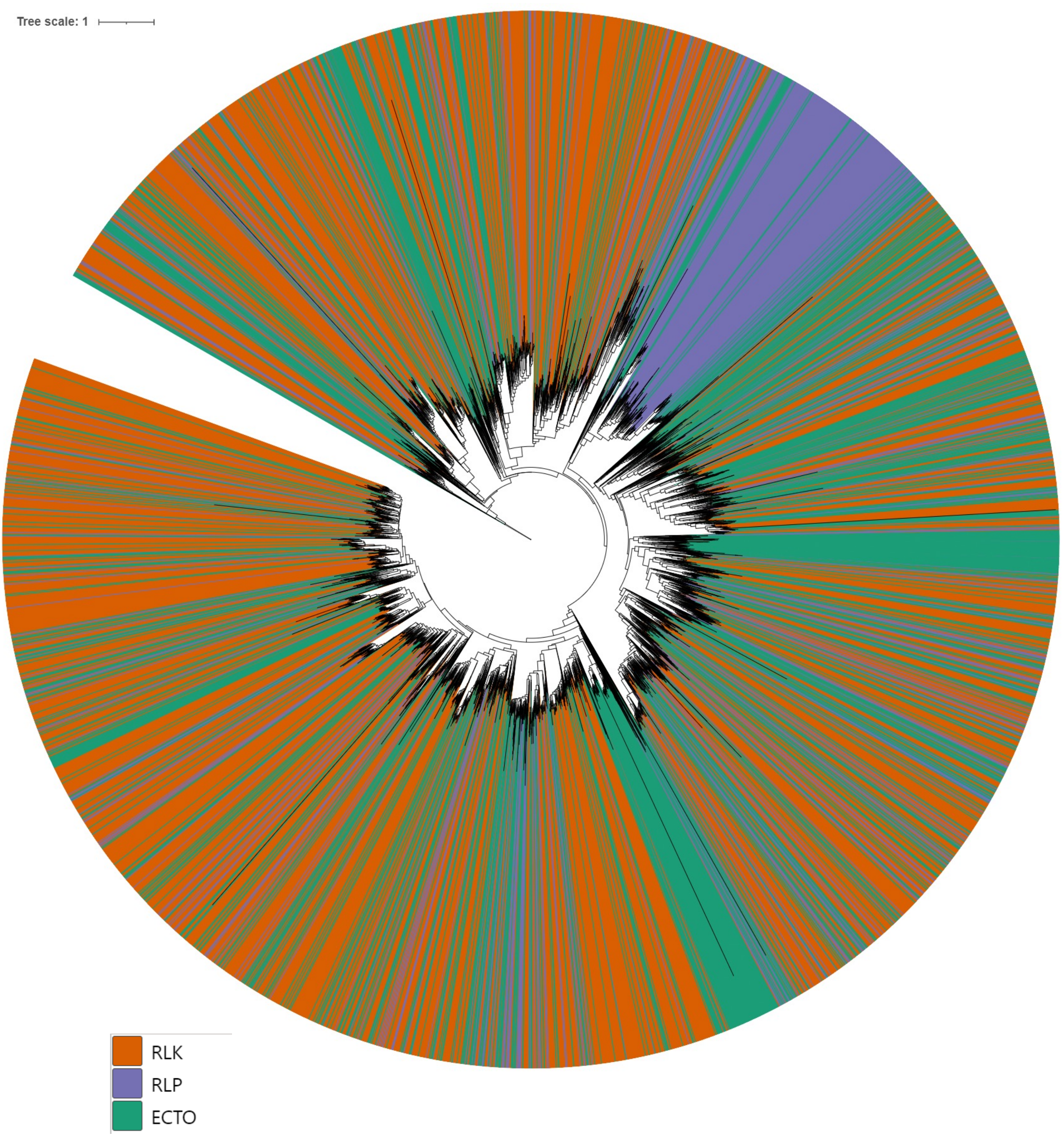

k

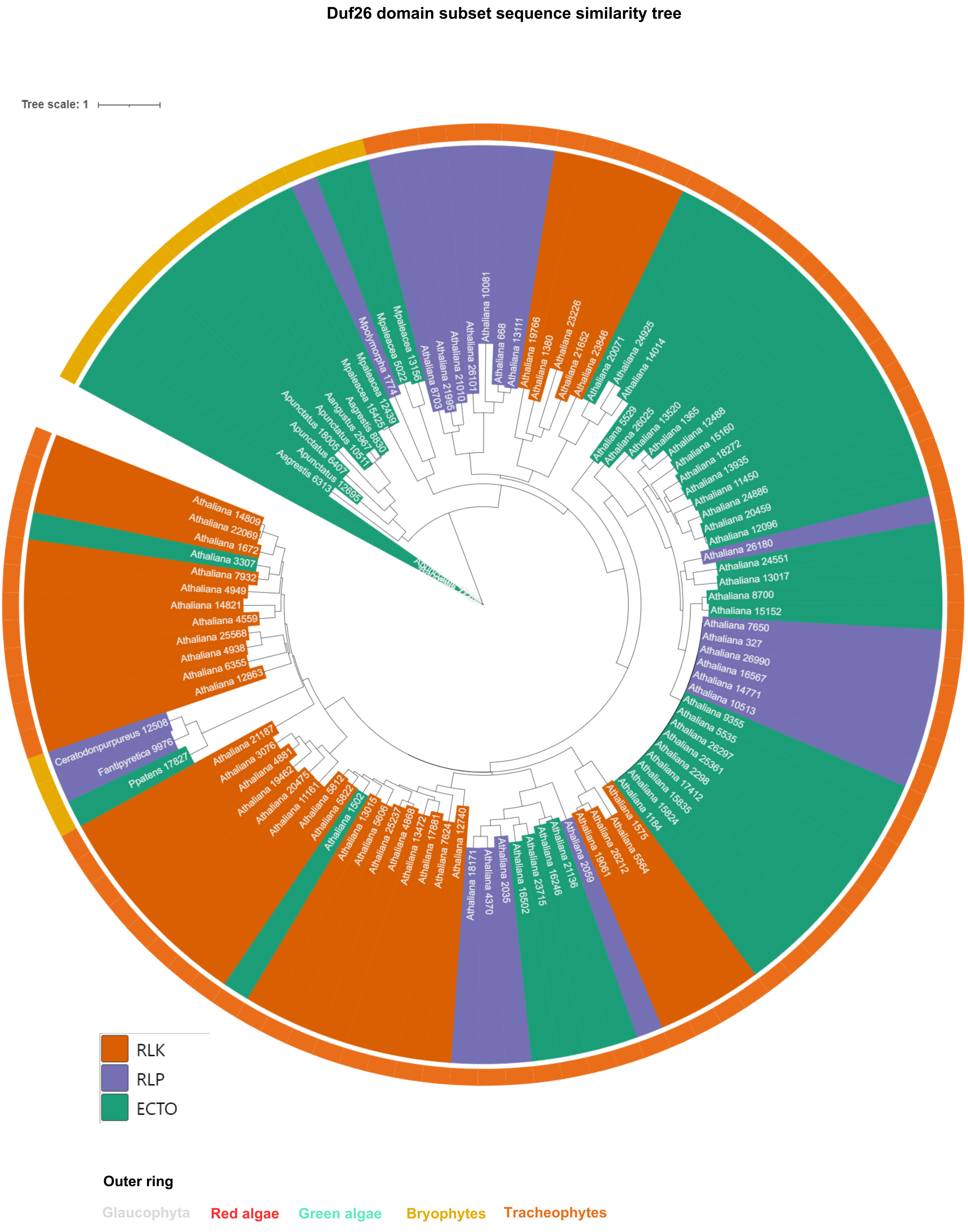
Duf26 domain subset sequence similarity tree
Tree scale: 1
RLK
RLP
ECTO
Outer ring
Glaucophyta
Red algae
Green algae
Bryophytes
Tracheophytes

I

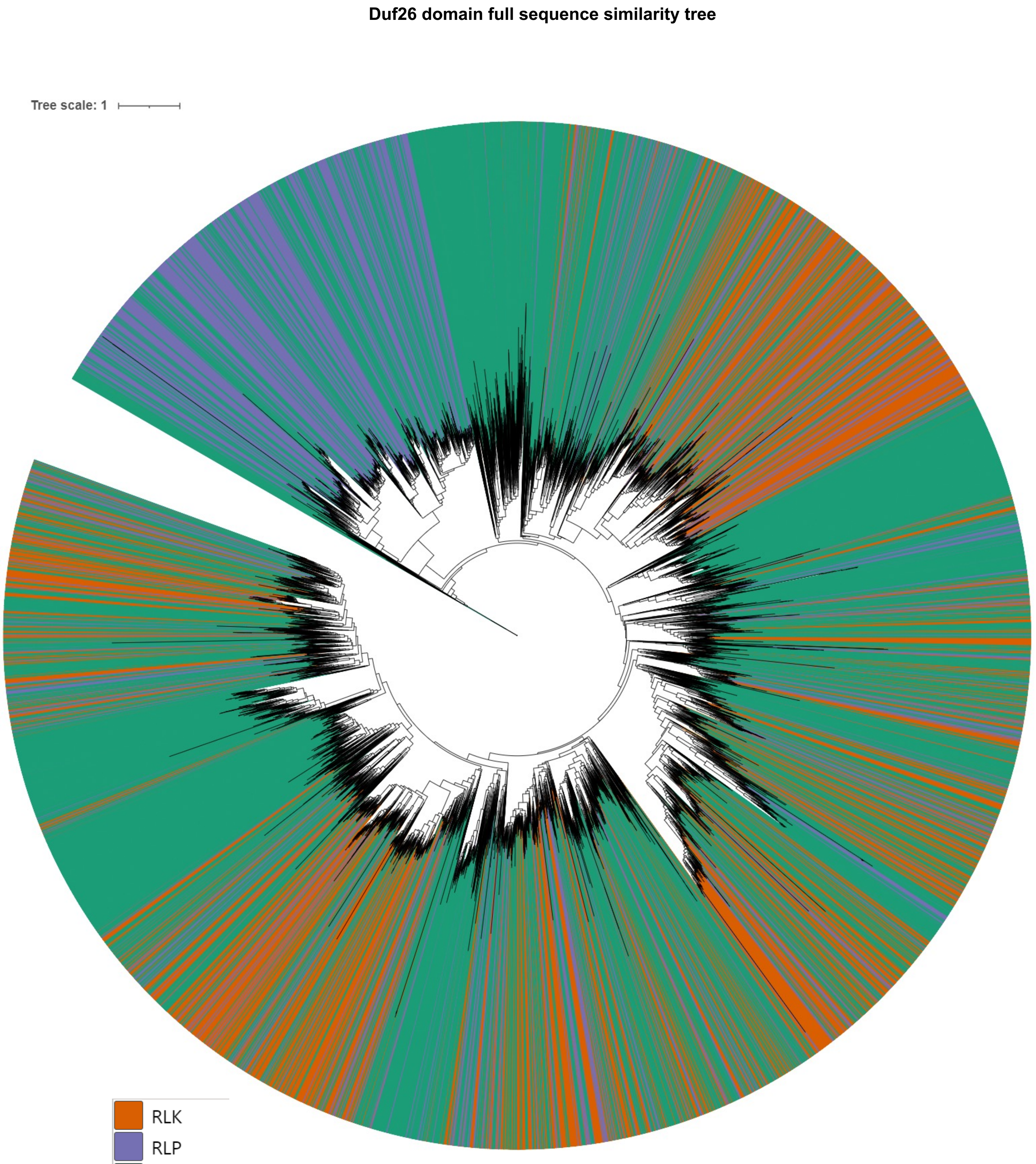
Duf26 domain full sequence similarity tree
Tree scale: 1
RLK
RLP
ECTO

Supplementary figure 4. Phylogenetic analysis of ectodomains from RLKs, RLPs and ectodomain-only proteins in plants. (a), (c), (e), (g), (i), (k), Sequence similarity tree of (a) WAK, (c) LysM, (e) Malectin, (g) G-lectin, (i) L-lectin and (k) Duf26 domains from RLKs (RLK), RLPs (RLP) and ectodomain-only proteins (ECTO) obtained from a subset of 25 species. These 25 species include *Cyanophora paradoxa, Chondrus crispus, Porphyra umbilicalis, Cyanidioschyzon merolae, Galdieria phlegrea , Galdieria sulphuraria , Micromonas commoda, Micromonas pusilla CCMP1545, Ostreococcus tauri, Ostreococcus lucimarinus, Coccomyxa subellipsoidea C-169, Botryococcus braunii, Chromochloris zofingiensis, Dunaliella salina, Volvox carteri, Chlamydomonas schloesseri CCAP 11/173, Chlamydomonas eustigma, Chlamydomonas incerta SAG 7.73, Chlamydomonas reinhardtii, Klebsormidium nitens , Mesostigma viride , Chlorokybus atmophyticus, Chara braunii, Spirogloea muscicola, Mesotaenium endlicherianum, Penium margaritaceum, Marchantia polymorpha, Marchantia paleacea, Anthoceros agrestis, Anthoceros punctatus, Anthoceros angustus, Sphagnum fallax, Sphagnum magellanicum, Fontinalis antipyretica, Ceratodon purpureus, Physcomitrium patens* and *Arabidopsis thaliana*. The outer ring indicates ectodomains from either Glaucophyta, red algae (Rhodophyta), green algae, Bryophytes or Tracheophytes. The branch-extension colour indicates if the ectodomain is obtained from RLKs (orange), RLPs (purple) or ectodomain-only proteins (green). **(b), (d), (f), (h), (j), (l),** Sequence similarity tree of (b) WAK, (d) LysM, (f) Malectin, (h) G-lectin, (j) L-lectin and (l) Duf26 domains from RLKs (RLK), RLPs (RLP) and ectodomain-only proteins (ECTO) obtained from 350 species. The branch-extension colour indicates if the ectodomain is obtained from RLKs (orange), RLPs (purple) or ectodomain-only proteins (green).

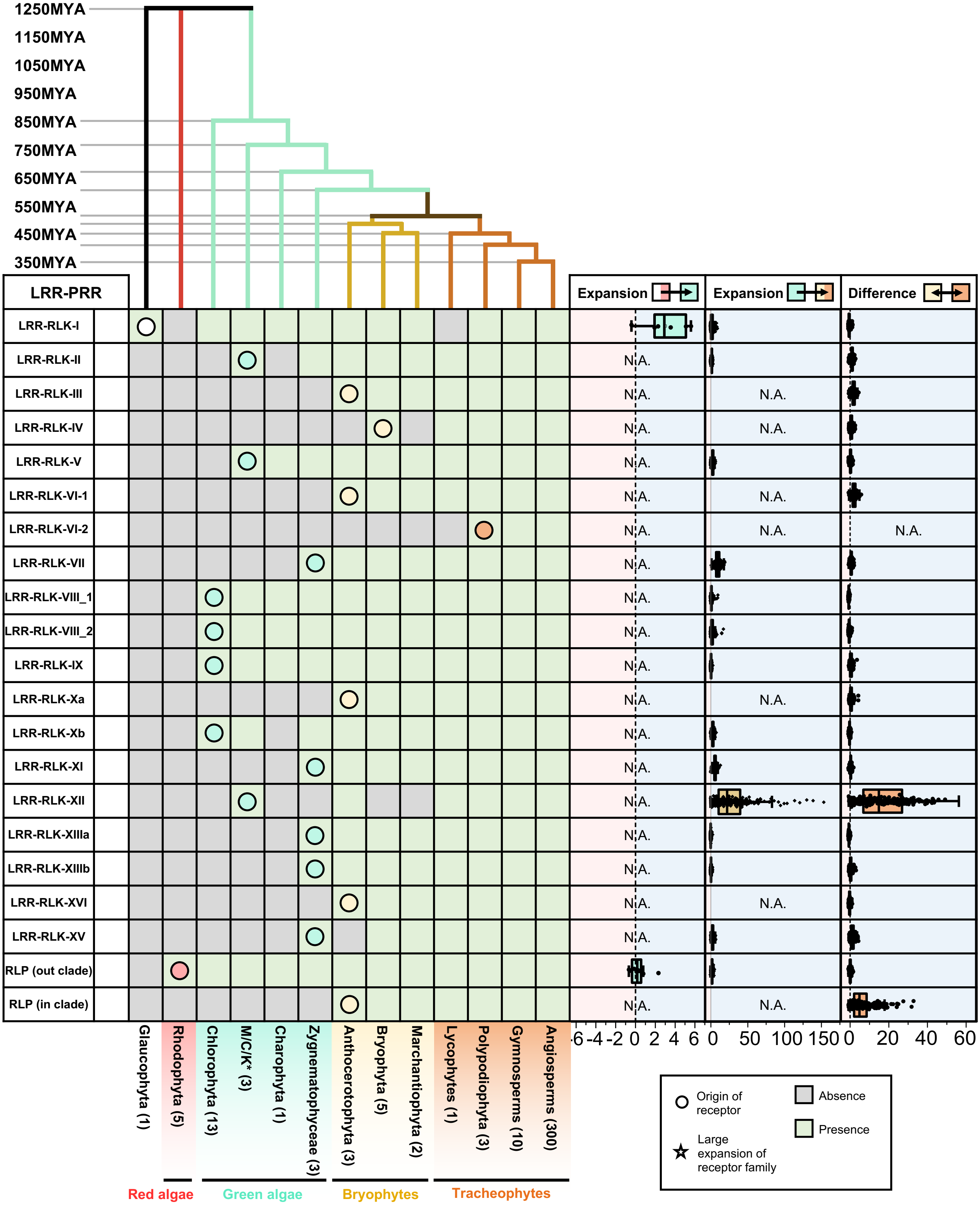

Supplementary figure 5. The origin and expansion of LRR-containing PRRs in plants. Top panel represents the sequence similarity tree of multiple algae and plant lineage. The timescale (in million years; MYA) of the sequence similarity tree is estimated by TIMETREE5[22]. Bottom panel represent the present/absence of different receptor classes in different algae and plant lineages. RLP represenst LRR-RLPs. 'Out clade' refers to LRR-RLPs outside the ID+4LRR clade in figure 4c and 'in clade' refers to LRR-RLPs inside the ID+4LRR clade in figure 4c. *M/C/K represents Mesostigmatophyceae, Chlorokybophyceae, and Klebsormidiophyceae. Number of available species from each algae and plant lineages are indicated by numbers in the boxes. Grey box indicates the absence of receptor and green box indicates the presence of receptors in each lineage. Origin of receptor is indicated with a circle (○). Expansion rate of receptor classes are indicated by boxplots. Cyan boxplot represents the expansion rate from Glaucophtya and Rhodophyta to green algae. Yellow boxplot represents the expansion rate from green algae to embryophytes and orange boxplot represents the expansion rate from early land plants to Tracheophytes. Boxplot elements: centre line, median; bounds of box, 25th and 75th percentiles; whiskers, 1.5 × IQR from 25th and 75th percentiles. n (number of LRR-RLK subclass members analyzed) is provided in the 'Protein counts per species' file on *Zenodo* (see Data availability section).

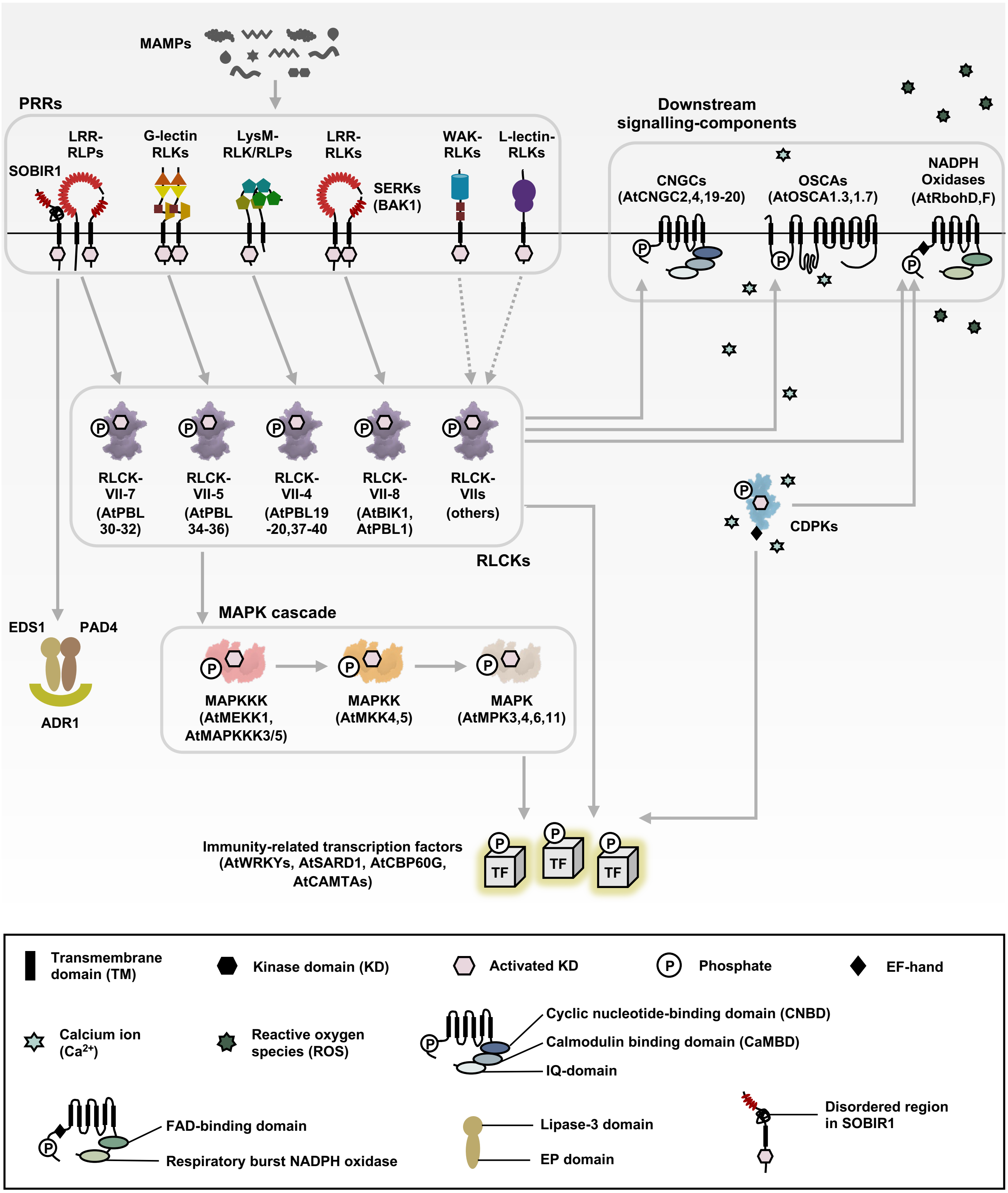

Supplementary figure 6. PTI signalling pathway in plants. Schematic figure represents the PTI signalling pathway in plants. The architecture of different signalling components is defined in the lower box. Grey arrow represents the activation of downstream signalling components by upstream signalling components following the perception of PAMPs/MAMPs by PRRs. Multiple signalling components are activated through phosphorylation by an activated kinase domain (KD). Downstream signalling components are activated to produce ROS and cytosolic calcium influx. Multiple immune-related transcription factors (TF) are also activated by cytoplasmic protein kinases, which then leads to defence-related gene expression.

PTI signalling components			Immunity-related	Reproduction-related	Development-related	Method of identification from genome
Type	Protein	Icon	Example	Example	Example	
PRR co-receptor	SERKs	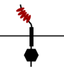	AtBAK1 (AtSERK3), OsBAK1 (OsSERK2)	SERKs	SERKs	Identification of SERK clade from previously published LRR-RLK-II sequence similarity tree[79].
	SOBIR1	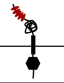	AtSOBIR1, NbSOBIR1, SlSOBIR1	AtSOBIR1	AtSOBIR1	Identification of SOBIR1 clade from LRR-RLK-XI-2 members from HMM search[79]. Confirm through checking SOBIR1 orthologs.
RLCKs	RLCK-VII	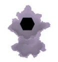	AtBIK1, AtPBL1, AtPBL19-20, 30-32, 34-40	*Other RLCK subgroups are involved	*Other RLCK subgroups are involved	Identification of RLCK-VII clade from RLCK members from HMM search using published HMM profiles[79]. Confirm through checking RLCK-VII orthologs.
CDPKs (CPKs)	-	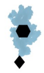	AtCPK1, AtCPK2, AtCPK5, AtCPK6, AtCPK11, AtCPK28	AtCPK2, AtCPK11, AtCPK20, AtCPK24, AtCPK33	AtCPK11, AtCPK12, AtCPK28,AtCPK30, AtCPK32	Identification of CDPK clade from CAMK_CDPK members from HMM search[79]. Confirm through checking CDPK orthologs.
MAPKs	MAPKKK (MEKK)	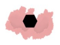	AtMEKK1, AtMAPKKK3/5	MAPK signalling cascade is also involved in floral organ development, inflorescence architecture and embryogenesis.	MAPK signalling cascade is also involved in phytohormone synthesis and signalling, cell division, cell differentiation and other development processes.	Identification of MAPKKK clade from STE11 members from HMM search[79]. Confirm through checking MAPKKK orthologs.
	MAPKK (MKK)	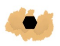	AtMKK4, AtMKK5			Identification of MAPKK clade from STE7 members from HMM search using published HMM profiles[79]. Confirm through checking MAPKK orthologs.
	MAPK	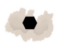	AtMPK3, AtMPK4, AtMPK6			Identification of MAPK clade from CMGC_MAPK members from HMM search[79]. Confirm through checking MAPK orthologs.
Calcium channels	CNGC	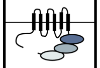	AtCNGC2,4,19,20	AtCNGC8, AtCNGC16, AtCNGC18	AtCNGC5, AtCNGC6, AtCNGC9	Search for proteins with PFAM profiles PF00520 and PF00027. Confirm through checking CNGC orthologs.
	OSCA	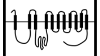	AtOSCA1.3, AtOSCA1.7			Search for proteins with PFAM profiles PF14703, PF02714 and PF13967. Confirm through checking OSCA orthologs.
NADPH oxidases	-	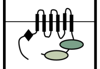	AtRbohD, AtRbohF	AtRbohH and AtRbohJ	AtRbohC, AtRbohD, AtRbohF	Search for proteins with HMM profiles: PF01794, PF0803, PF08414. Confirm through checking NADPH oxidase orthologs.
EP proteins	-	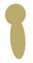	AtEDS1, AtPAD4, AtSAG101			Search for proteins with HMM profiles: PF18117 and PF01764. Cluster candidate genes and assign groups based on the known members from *A.thaliana*.
Helper NLRs	-	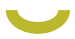	AtADR1, AtNRG1			Search for proteins with HMM profiles: PF00931 and PF05659. Cluster candidate genes and assign groups based on the known members from *A.thaliana*.
Immune-related TFs		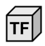	WRKYs, AtSARD1, AtCBP60G, CAMTAs			-

Supplementary figure 7. Roles of signalling components in different biological processes in plants. Table representing the characterized PTI signalling components in plants and the biological processes they are involved in. Characterized examples of each signalling component involved in different biological processes are given. Grey box indicates that signalling component has not been reported to involved in that biological process. The methods of identifying these signalling components from genomes are also stated. Abbreviations for plant species: *A. thaliana*, *At*; *S. lycopersicum*, *Sl*; *N. benthamiana*, *Nb.* References to the genes are included in the supplementary information.

a

SERK sequence similarity tree

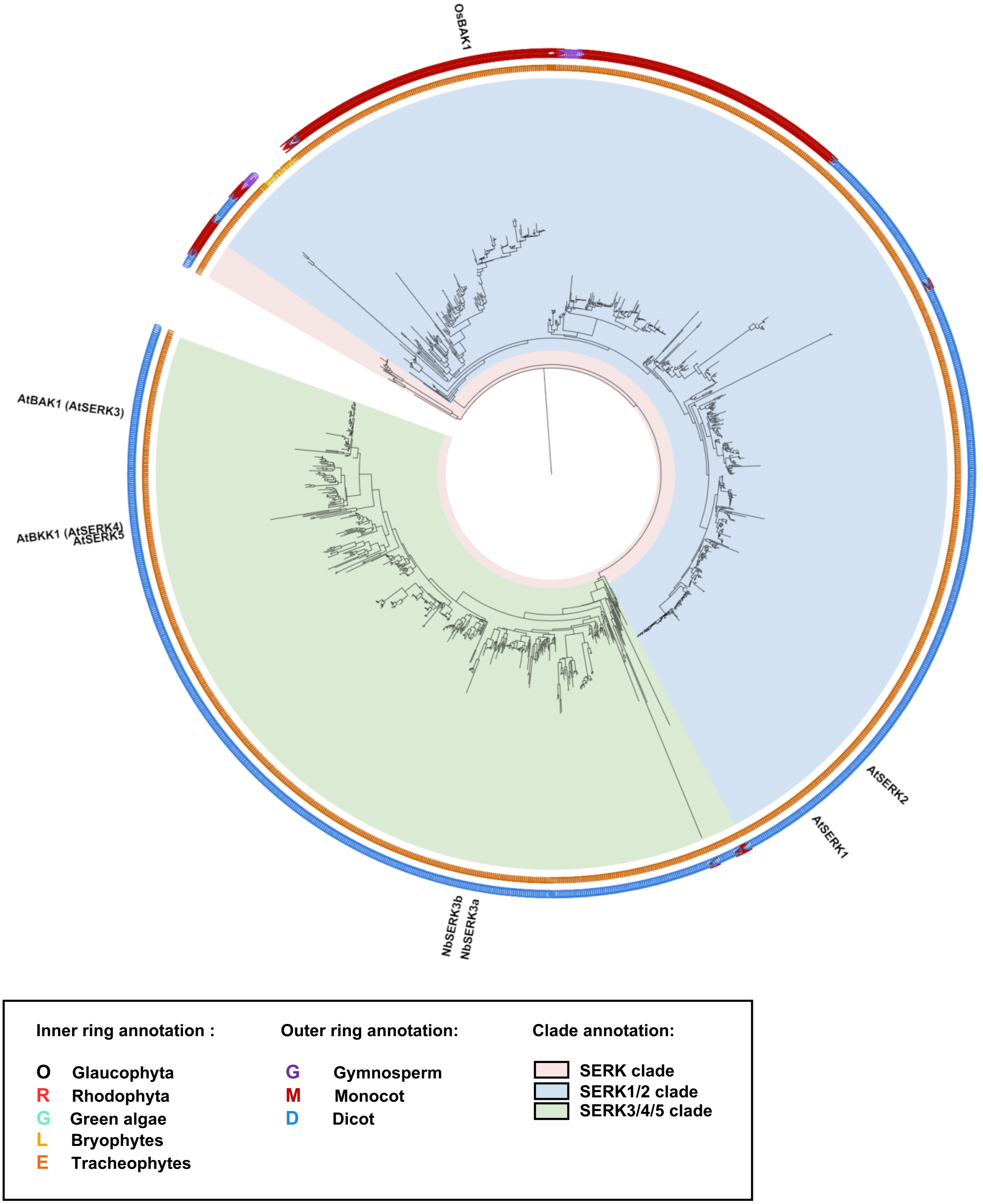

Supplementary figure 8a. Phylogenetic analysis of SERKs in plants. Full-length sequence similarity tree of SERK members identified from the LRR-RLK-II sequence similarity tree. The inner ring indicates SERK members from Glaucophyta, red algae (Rhodophyta), green algae, Bryophytes or Tracheophytes. The outer ring indicates LRR-RLK-II members from gymnosperm, monocots or dicots. The SERK, SERK1/2 and SERK3/4/5 clades are defined. Characterized SERK members are labelled in the tree. Abbreviations for plant species: *A. thaliana*, At; *N. benthamiana*, Nb; *O.sativa*, Os.

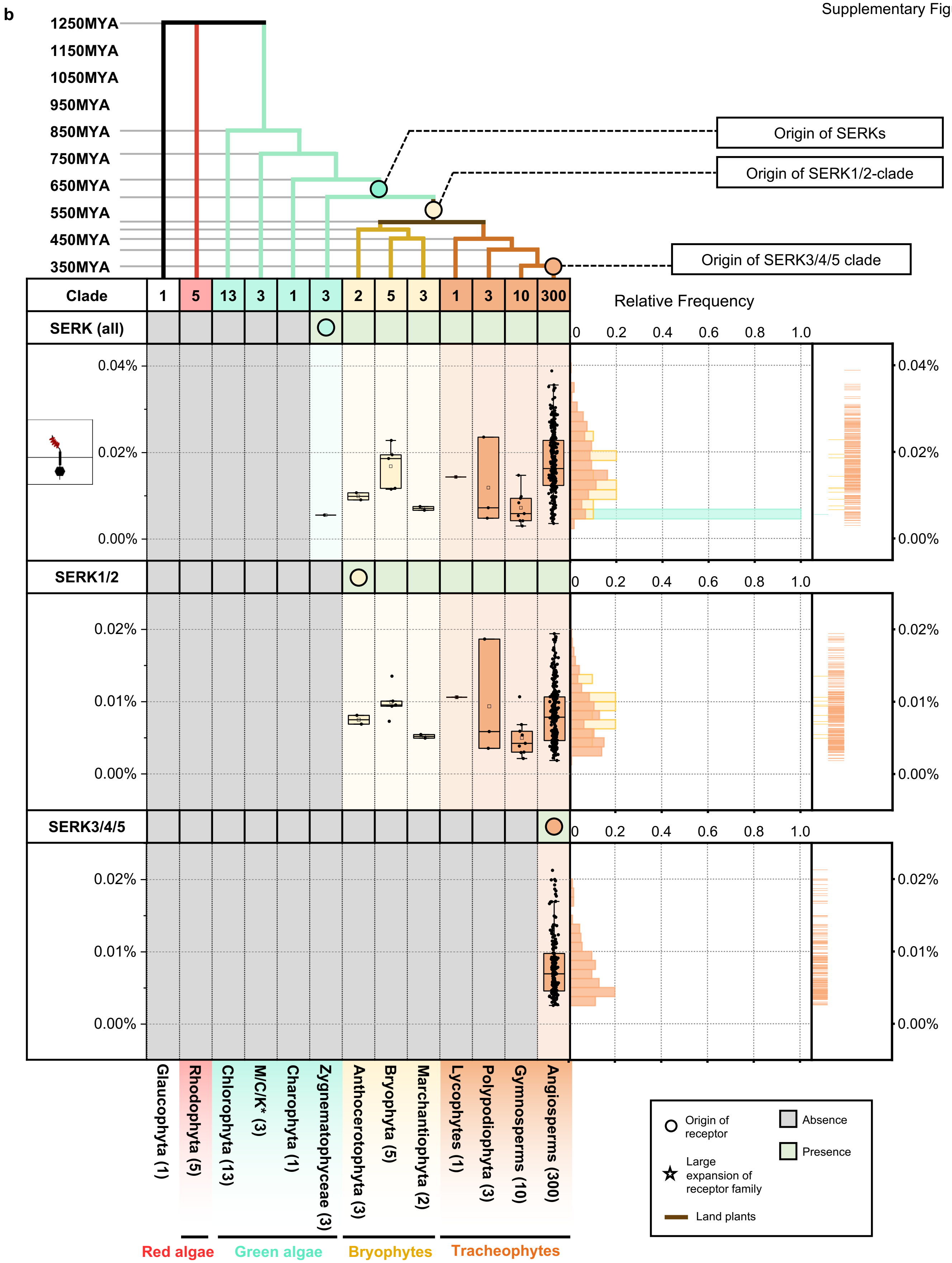

Supplementary figure 8b. The origin and expansion of SERKs in plants. Top panel represents the sequence similarity tree of multiple algae and plant lineage. With circle (○) and star (☆) indicate the origin and expansion of receptor families. The timescale (in million year; MYA) of the sequence similarity tree is estimated by TIMETREE. Bottom panel represent the present/absence of SERKs, SERK1/2-related and SERK3/4/5-related proteins in different algae and plant lineages. Grey box indicates the absence of receptor and green box indicates the presence of receptors in each lineage. The origin of SERKs are marked with a circle. *M/C/K represents Mesostigmatophyceae, Chlorokybophyceae and Klebsormidiophyceae. Number of available species from each algae and plant lineages are indicated by numbers in the boxes. Boxplot below represents the percentage (%) and right plot represents the distribution and rug of the relative frequency of the % of SERKs, SERK1/2-related and SERK3/4/5-related proteins in each lineage. Boxplot elements: centre line, median; bounds of box, 25th and 75th percentiles; whiskers, 1.5 × IQR from 25th and 75th percentiles. n (number of downstream signalling component analyzed) is provided in the 'Protein counts per species' file on *Zenodo* (see Data availability section).

c

SOBIR1 sequence similarity tree

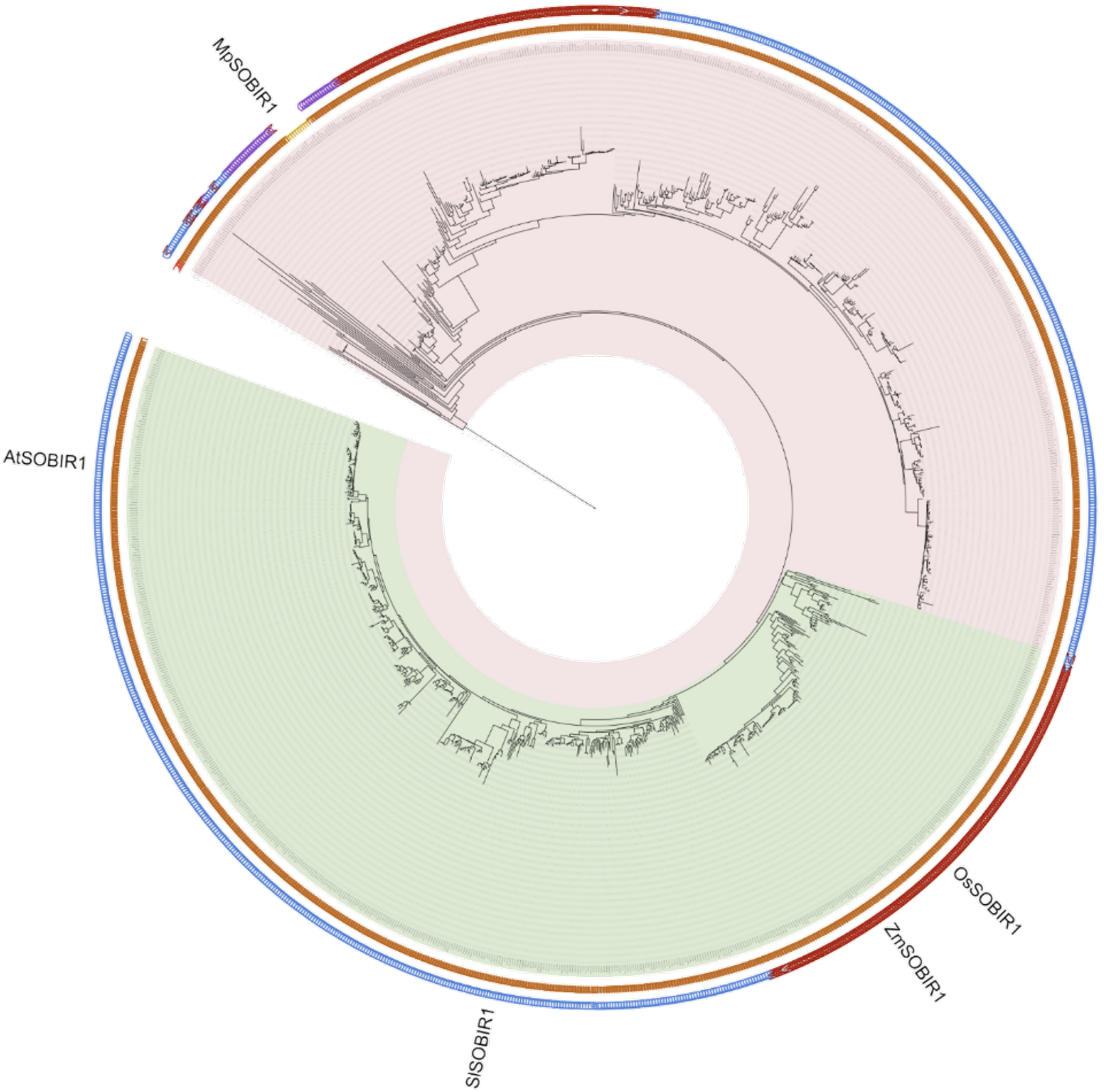

Inner ring annotation :		Outer ring annotation:		Clade annotation:
O	Glaucophyta	G	Gymnosperm	SOBIR1/SOBIR1-related clade
R	Rhodophyta	M	Monocot	Angiosperm SOBIR1 clade
G	Green algae	D	Dicot	
L	Bryophytes			
E	Tracheophytes			

Supplementary figure 8c. Phylogenetic analysis of SOBIR1 in plants. Sequence similarity tree of full length SOBIR1 members identified from 350 species. The inner ring indicates SOBIR1 members from Glaucophyta, red algae (Rhodophyta), green algae, Bryophytes or Tracheophytes. The outer ring indicates SOBIR1 members from gymnosperm, monocots or dicots. The SOBIR1/SOBIR1-related and angiosperm SOBIR1 clades are defined. Characterized SOBIR1 members are labelled in the tree. Abbreviations for plant species: *M. polymorpha*, Mp; *A. thaliana*, At; *S. lycopersicum*, Sl; *O. sativa,* Os; *Z. mays*, ZM.

d

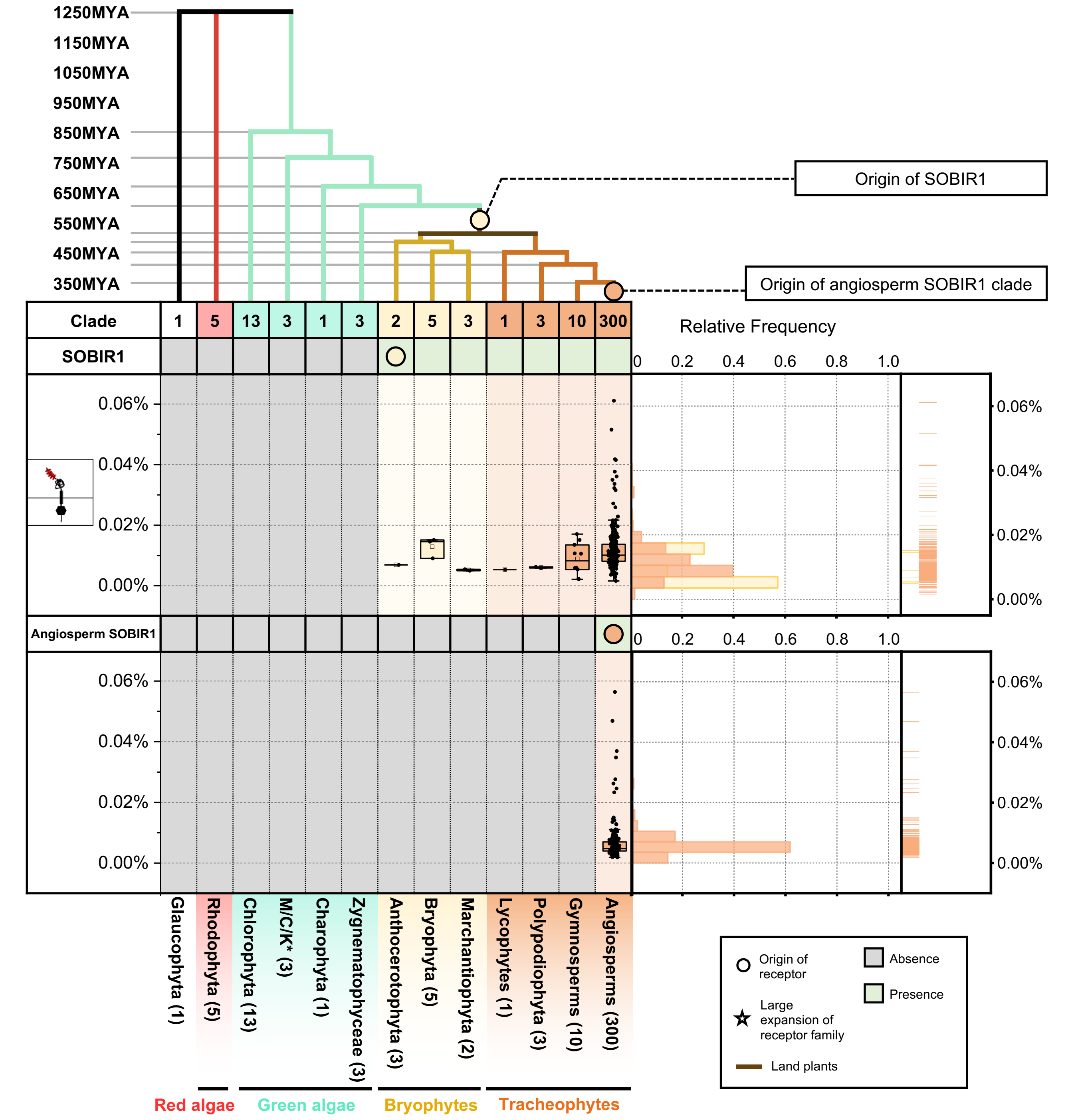

Supplementary figure 8d. The origin and expansion of SOBIR1 in plants. Top panel represents a sequence similarity tree of multiple alga and plant lineages. Circles (○) and stars (☆) indicate the origin and expansion of receptor families. The timescale (in million years; MYA) of the sequence similarity tree is estimated by TIMETREE. Bottom panel represents the presence/absence of SOBIR1 and angiosperm SOBIR1 in different algae and plant lineages. Grey box indicates the absence of receptor and green box indicates the presence of receptors in each lineage. The origin of SOBIR1 is marked with a circle. *M/C/K represents Mesostigmatophyceae, Chlorokybophyceae and Klebsormidiophyceae. Number of available species from each algal and plant lineage are indicated by numbers in the boxes. Boxplot below represents the percentage (%) and right plot represents the distribution and rug of the relative frequency of the % of SOBIR1 and angiosperm SOBIR1 in each lineage. Boxplot elements: centre line, median; bounds of box, 25th and 75th percentiles; whiskers, 1.5 × IQR from 25th and 75th percentiles.

e

RLCK-VII sequence similarity tree

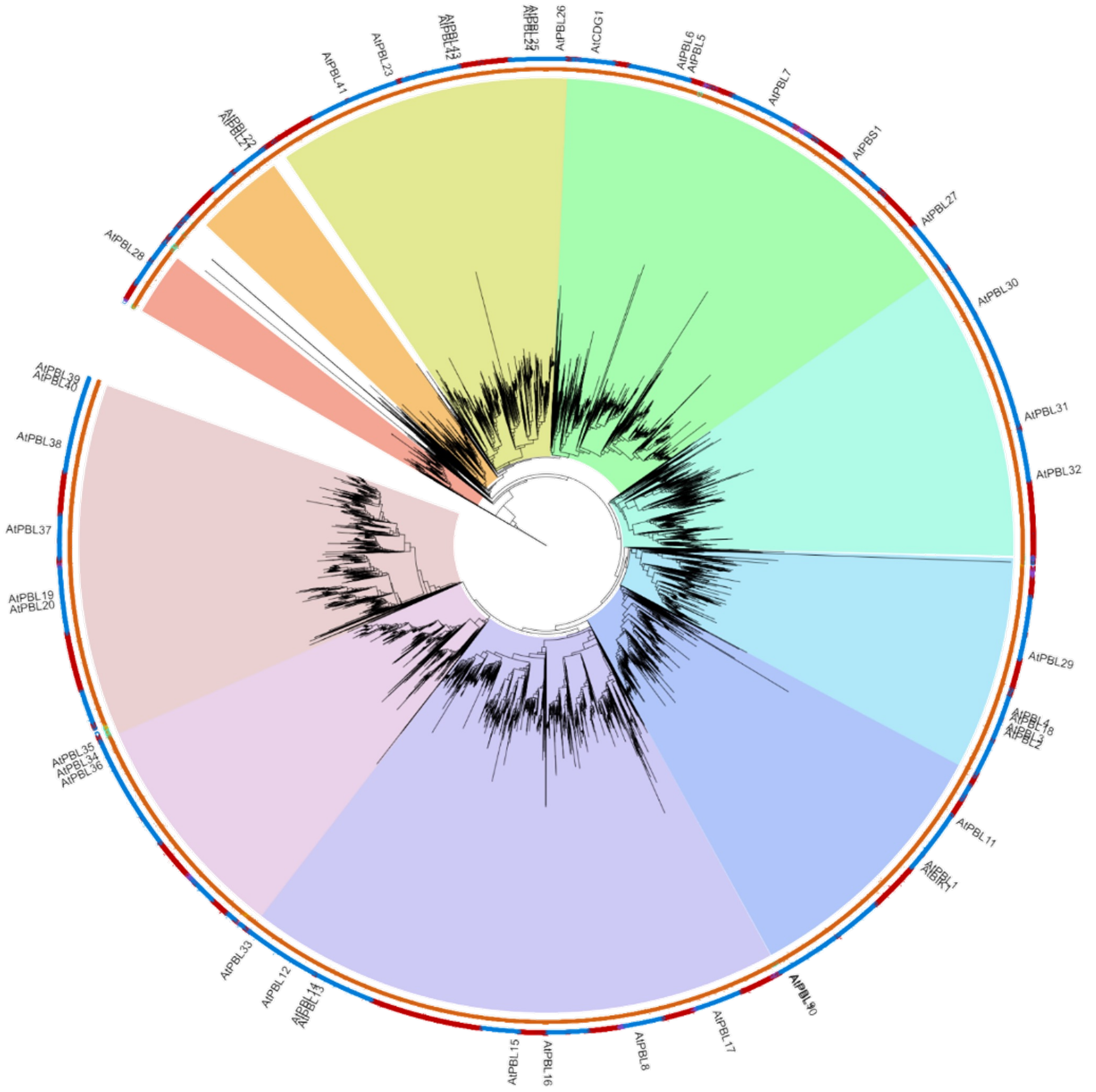

Inner ring annotation :

- **O** Glaucophyta
- **R** Rhodophyta
- **G** Green algae
- **L** Bryophytes
- **E** Tracheophytes

Outer ring annotation:

- **G** Gymnosperm
- **M** Monocot
- **D** Dicot

Clade annotation:

- RLCK-VII clade
- RLCK-VII-10 clade (AtPBL28)
- RLCK-VII-3 clade (AtPBL21,22)
- RLCK-VII-2 clade (AtPBL23-26,41-43)
- RLCK-VII-1 clade (AtPBL5-7,27, AtPBS1, AtCDG1)
- RLCK-VII-7 clade (AtPBL30-32)
- RLCK-VII-9 clade (AtPBL2-4,18,29)
- RLCK-VII-8 clade (AtPBL9-11, AtBIK1, AtPBL1)
- RLCK-VII-6 clade (AtPBL8,12-17,33)
- RLCK-VII-5 clade (AtPBL34-36)
- RLCK-VII-4 clade (AtPBL19,20,37-40)

Supplementary figure 8e. Phylogenetic analysis of RLCK-VII in plants. Sequence similarity tree of full length RLCK-VII members identified from 350 species. The inner ring indicates RLCK-VII members from either Glaucophyta, red algae (Rhodophyta), green algae, Bryophytes or Tracheophytes. The outer ring indicates RLCK-VII members from either gymnosperm, monocots or dicots. The 10 RLCK-VII clades are defined based on previous annotation[101]. *Arabidopsis thaliana* RLCK-VIIs members are labelled in the tree. Abbreviations for plant species: *A. thaliana*, At.

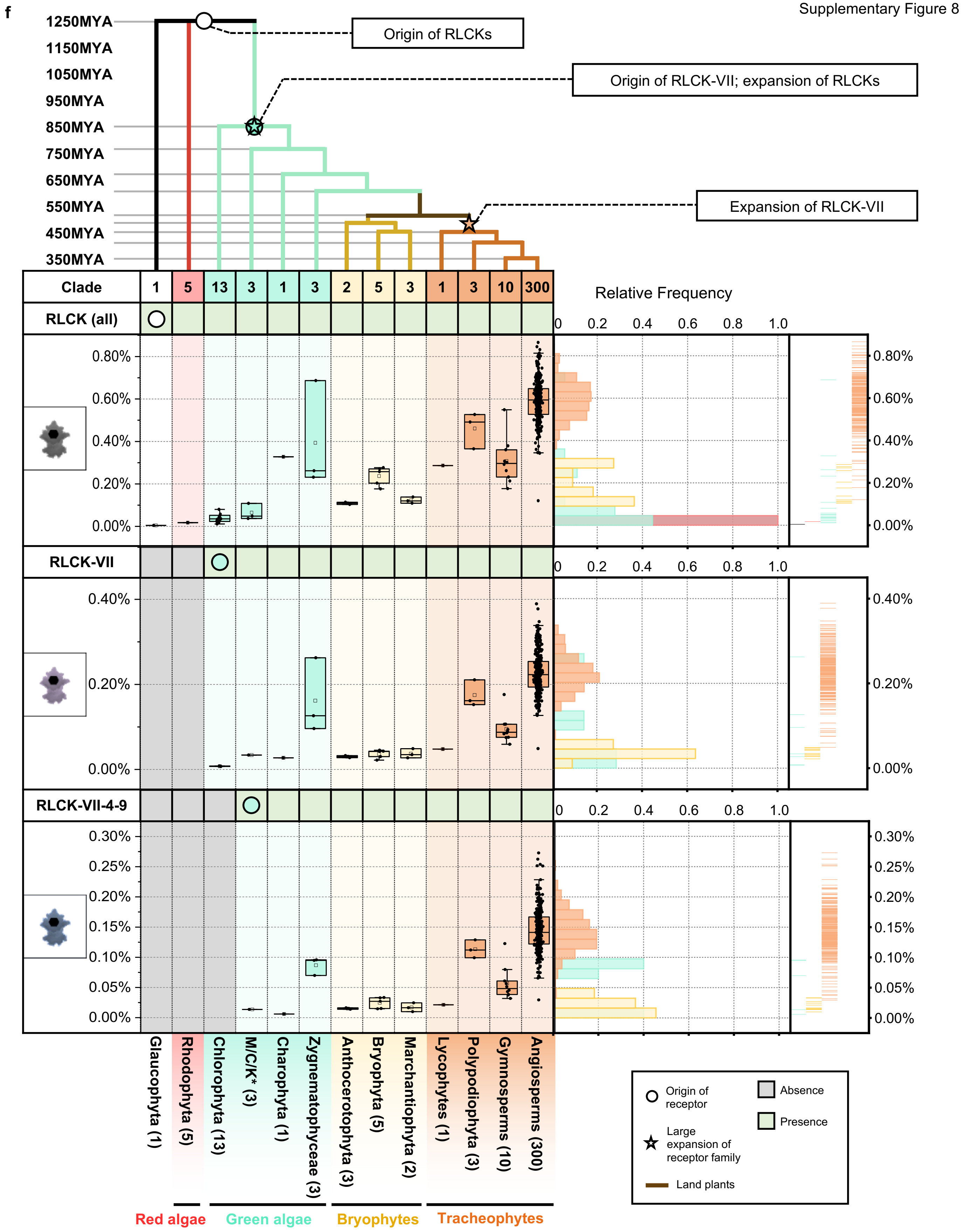

Supplementary figure 8f. The origin and expansion of RLCKs in plants. Top panel represents the sequence similarity tree of multiple algae and plant lineage. With circle (○) and star (☆) indicate the origin and expansion of receptor families. The timescale (in million year; MYA) of the sequence similarity tree is estimated by TIMETREE. Bottom panel represent the present/absence of RLCKs, RLCK-VII and RLCK-VII-4,5,6,7,8,9 in different algae and plant lineages. Grey box indicates the absence of receptor and green box indicates the presence of receptors in each lineage. The origin of RLCKs are marked with a circle. *M/C/K represents Mesostigmatophyceae, Chlorokybophyceae and Klebsormidiophyceae. Number of available species from each algae and plant lineages are indicated by numbers in the boxes. Boxplot below represents the percentage (%) and right plot represents the distribution and rug of the relative frequency of the % of RLCKs, RLCK-VII and RLCK-VII-4,5,6,7,8,9 in each lineage. Boxplot elements: centre line, median; bounds of box, 25th and 75th percentiles; whiskers, 1.5 × IQR from 25th and 75th percentiles. n (number of downstream signalling component analyzed) is provided in the 'Protein counts per species' file on *Zenodo* (see Data availability section).

g

CDPK sequence similarity tree

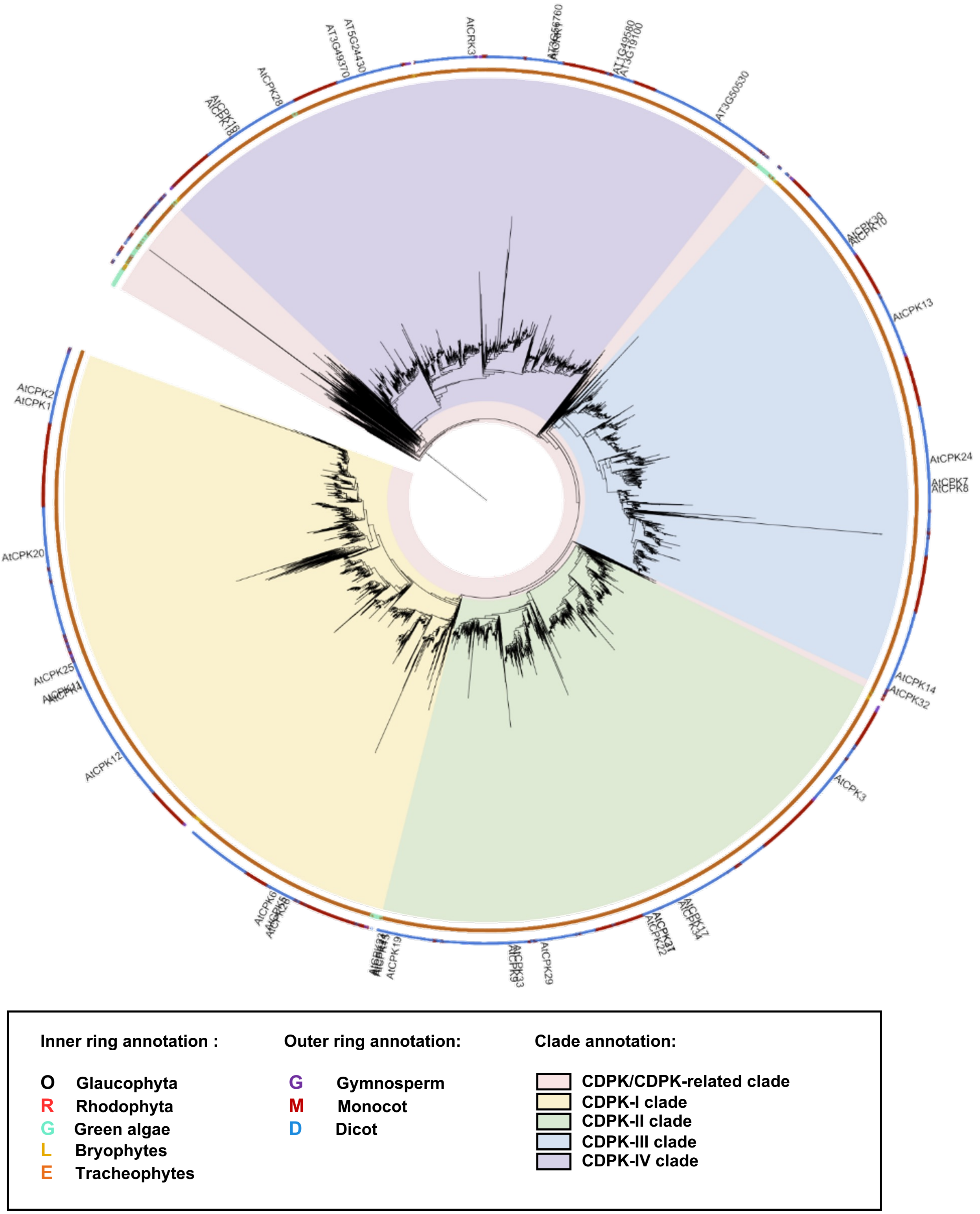

Supplementary figure 8g. Phylogenetic analysis of CDPK in plants. Sequence similarity tree of full length CDPK members identified from 350 species. The inner ring indicates CDPK members from either Glaucophyta, red algae (Rhodophyta), green algae, Bryophytes or Tracheophytes. The outer ring indicates CDPK members from either gymnosperm, monocots or dicots. The CDPK-I/II/III/IV clades are defined based on previous annotation[102,103]. *Arabidopsis thaliana* CDPK members are labelled in the tree. Abbreviations for plant species: *A. thaliana*, At.

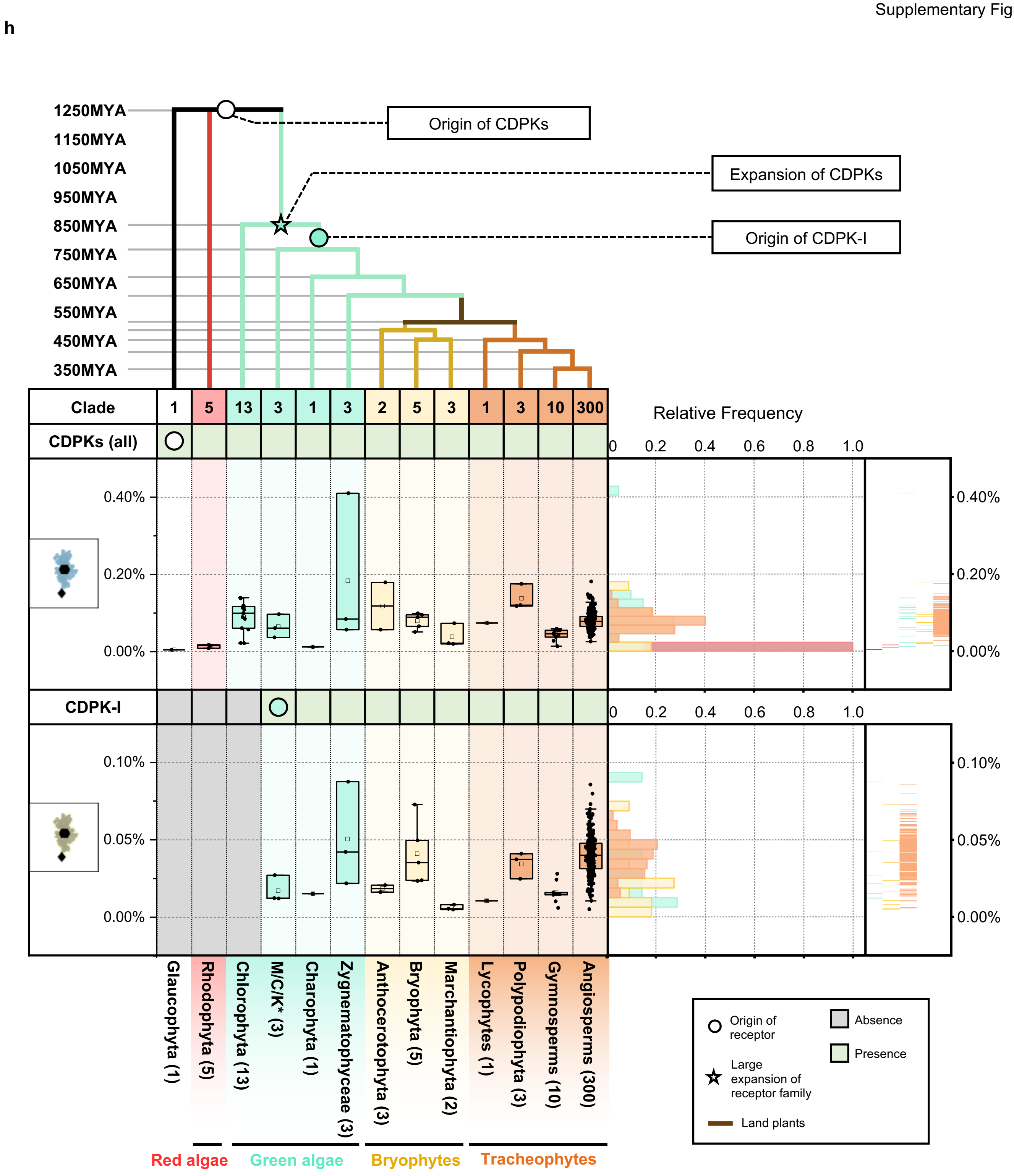

Supplementary figure 8h. The origin and expansion of CDPK in plants. Top panel represents the sequence similarity tree of multiple algae and plant lineage. With circle (○) and star (☆) indicate the origin and expansion of receptor families. The timescale (in million year; MYA) of the sequence similarity tree is estimated by TIMETREE. Bottom panel represent the present/absence of CDPK (all) and CDPK-I in different algae and plant lineages. Grey box indicates the absence of receptor and green box indicates the presence of receptors in each lineage. The origin of CDPKs are marked with a circle. *M/C/K represents Mesostigmatophyceae, Chlorokybophyceae and Klebsormidiophyceae. Number of available species from each algae and plant lineages are indicated by numbers in the boxes. Boxplot below represents the percentage (%) and right plot represents the distribution and rug of the relative frequency of the % of CDPK (all) and CDPK-I in each lineage. Boxplot elements: centre line, median; bounds of box, 25th and 75th percentiles; whiskers, 1.5 × IQR from 25th and 75th percentiles. n (number of downstream signalling component analyzed) is provided in the ‘Protein counts per species’ file on *Zenodo* (see Data availability section).

i

MAPKKK sequence similarity tree

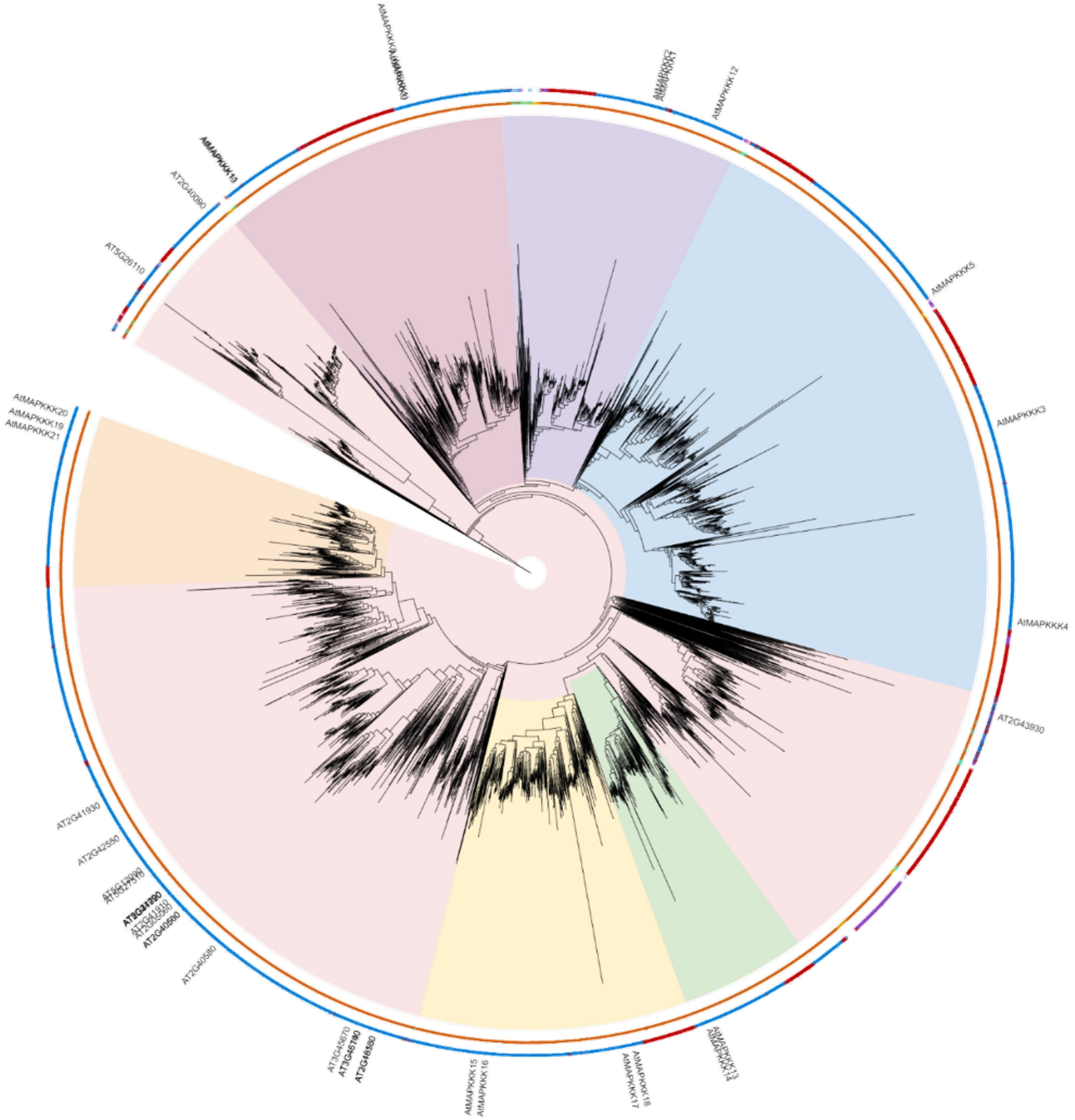

Inner ring annotation :		Outer ring annotation:		Clade annotation:
O	Glaucophyta	G	Gymnosperm	MAPKKK/MAPKKK-related clade
R	Rhodophyta	M	Monocot	MAPKKK clade I (AtMAPKKK8-11)
G	Green algae	D	Dicot	MAPKKK clade II (AtMAPKKK1,2, 12)
L	Bryophytes			MAPKKK clade III (AtMAPKKK3-5)
E	Tracheophytes			MAPKKK clade IV (AtMAPKKK13,14)
				MAPKKK clade V (AtMAPKKK15-16)
				MAPKKK clade VI (AtMAPKKK19-21)

Supplementary figure 8i. Phylogenetic analysis of MAPKKK in plants. Sequence similarity tree of MAPKKK members identified from 350 species. The inner ring indicates MAPKKK members from either Glaucophyta, red algae (Rhodophyta), green algae, Bryophytes or Tracheophytes. The outer ring indicates MAPKKK members from either gymnosperm, monocots or dicots. The MAPKKK-I/II/III/IV/V/VI clades are defined. *Arabidopsis thaliana* MAPKKKs members are labelled in the tree. Abbreviations for plant species: *A. thaliana*, At.

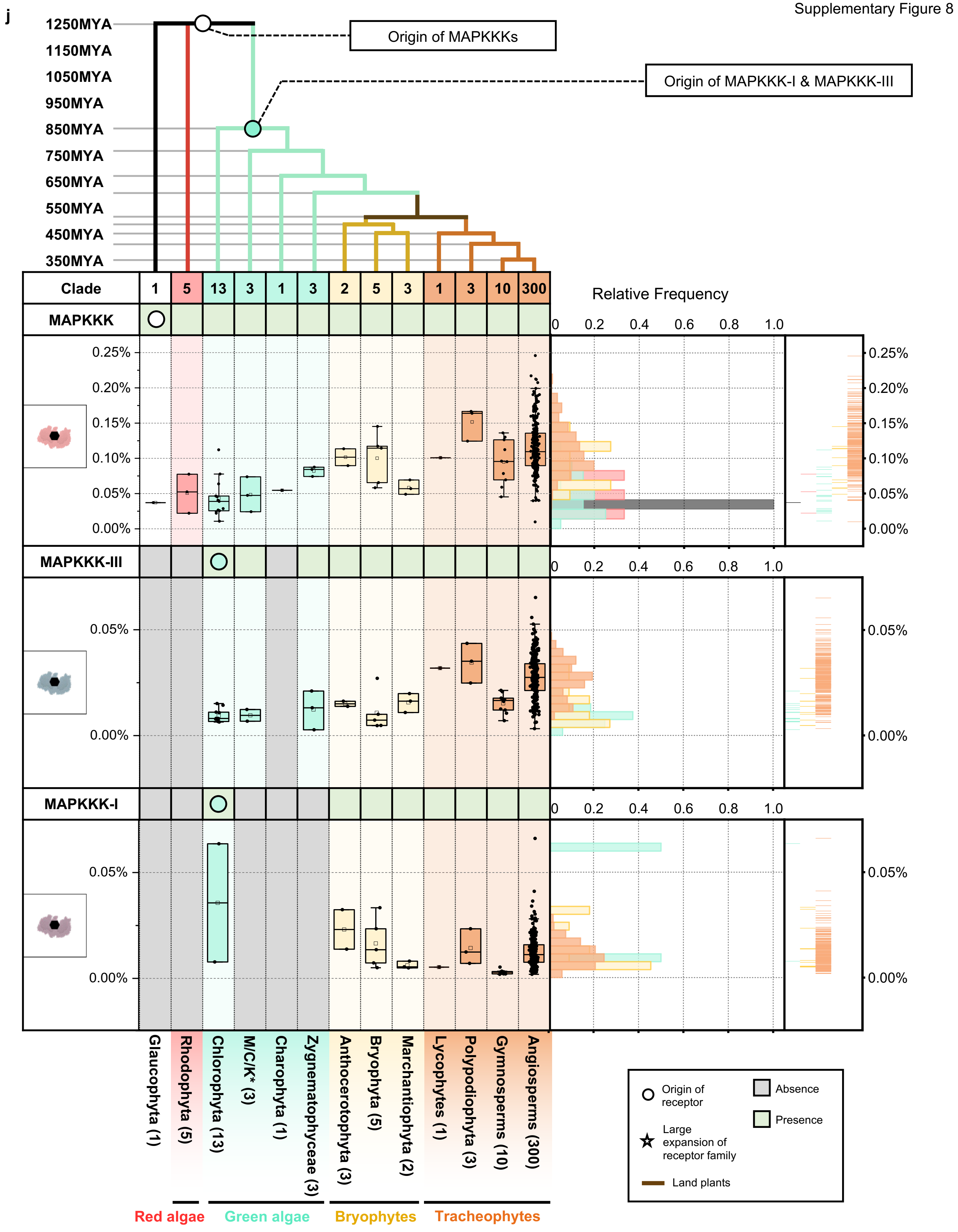

Supplementary figure 8j. The origin and expansion of MAPKKK in plants. Top panel represents the sequence similarity tree of multiple algae and plant lineage. With circle (○) and star (☆) indicate the origin and expansion of receptor families. The timescale (in million year; MYA) of the sequence similarity tree is estimated by TIMETREE. Bottom panel represent the present/absence of MAPKKK, MAPKKK-III and MAPKKK-I members in different algae and plant lineages. Grey box indicates the absence of receptor and green box indicates the presence of receptors in each lineage. The origin of MAPKKKs are marked with a circle. *M/C/K represents Mesostigmatophyceae, Chlorokybophyceae and Klebsormidiophyceae. Number of available species from each algae and plant lineages are indicated by numbers in the boxes. Boxplot below represents the percentage (%) and right plot represents the distribution and rug of the relative frequency of the % of MAPKKK, MAPKKK-III and MAPKKK-I members in each lineage. Boxplot elements: centre line, median; bounds of box, 25th and 75th percentiles; whiskers, 1.5 × IQR from 25th and 75th percentiles. n (number of downstream signalling component analyzed) is provided in the 'Protein counts per species' file on *Zenodo* (see Data availability section).

k

MAPKK sequence similarity tree

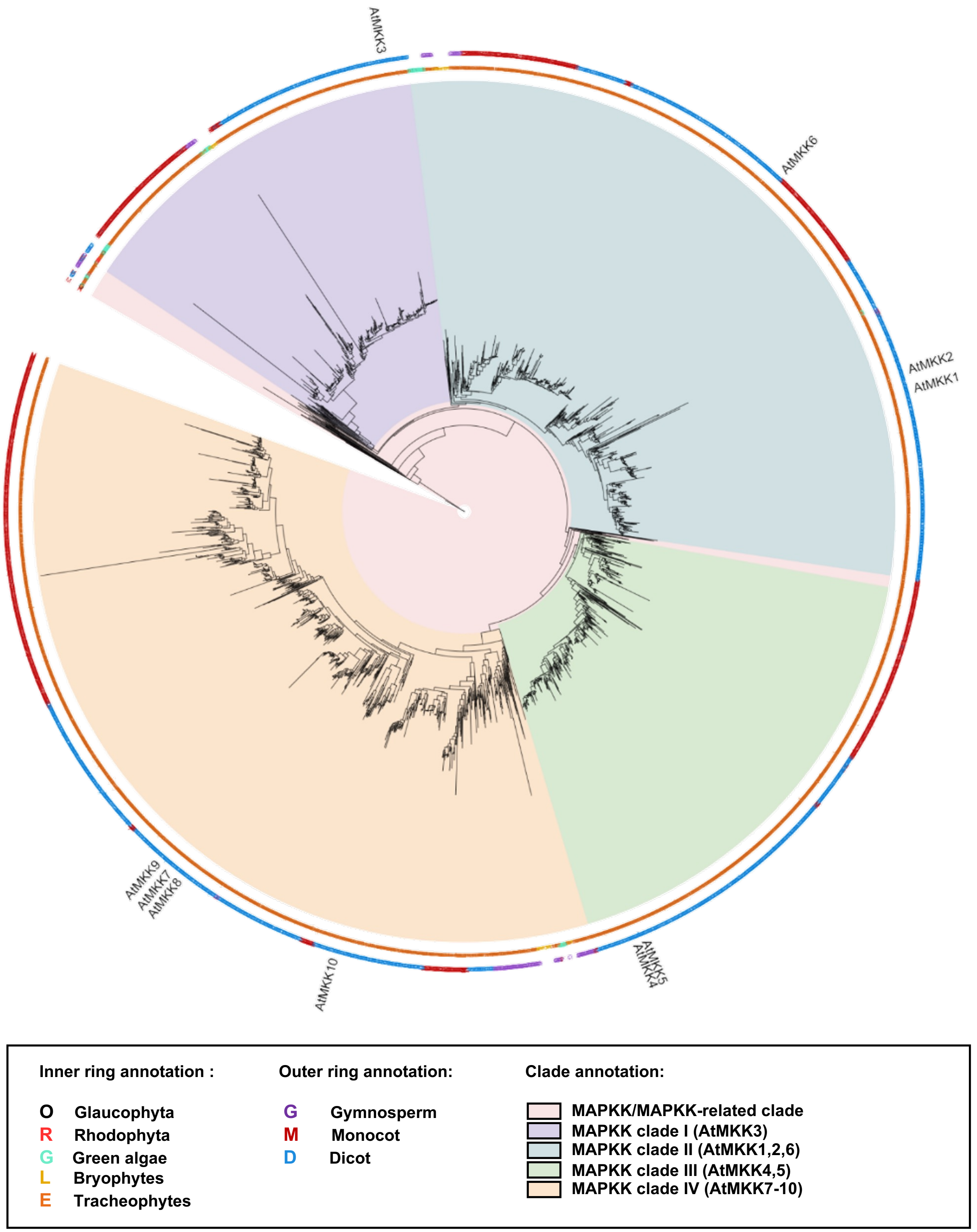

Supplementary figure 8k. Phylogenetic analysis of MAPKK in plants. Sequence similarity tree of MAPKK members identified from 350 species. The inner ring indicates MAPKK members from either Glaucophyta, red algae (Rhodophyta), green algae, Bryophytes or Tracheophytes. The outer ring indicates MAPKK members from either gymnosperm, monocots or dicots. The MAPKK-I/II/III/IV clades are defined based on previous annotation[104]. *Arabidopsis thaliana* MAPKKs members are labelled in the tree. Abbreviations for plant species: *A. thaliana*, At.

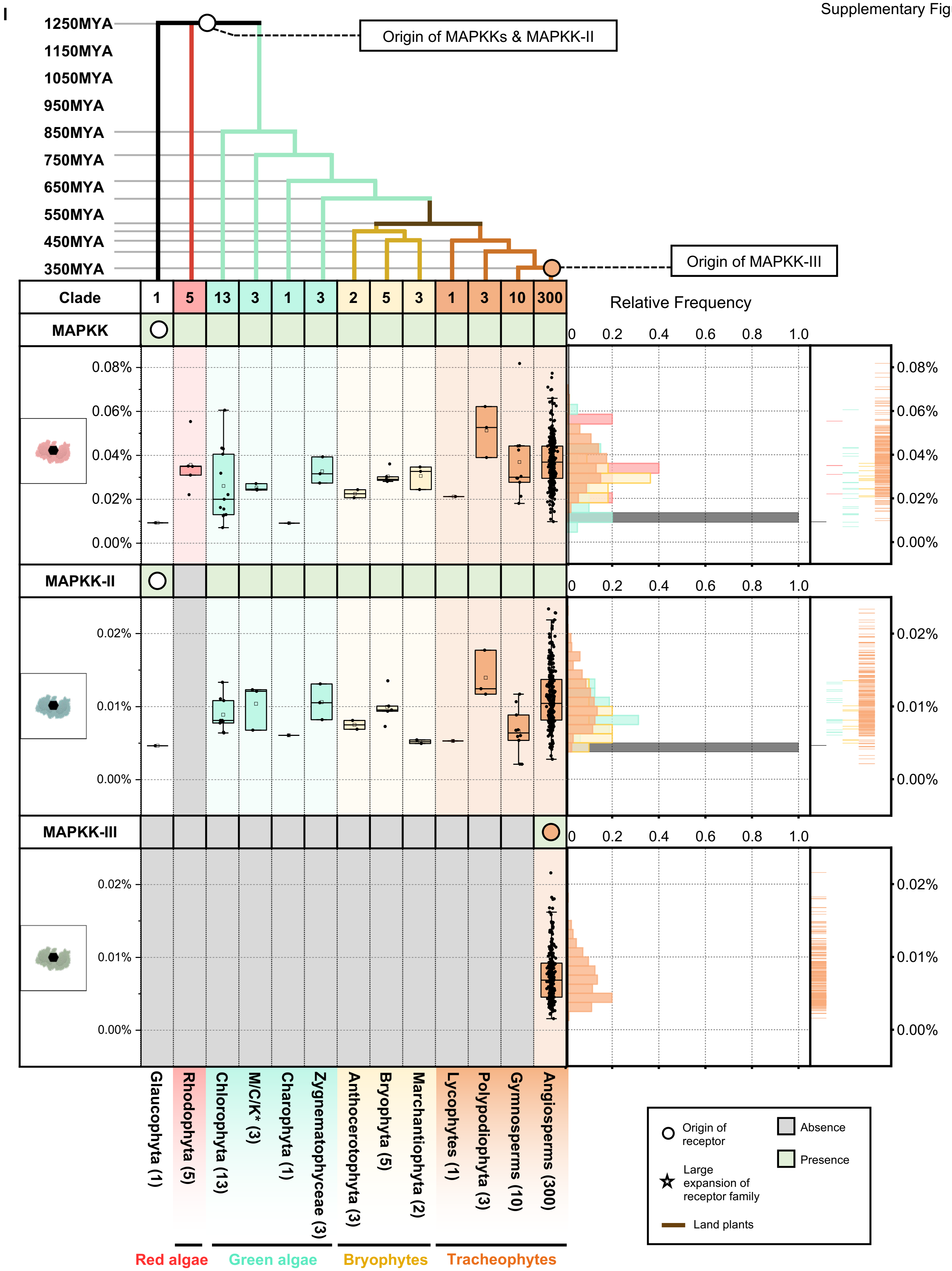

Supplementary figure 8I. The origin and expansion of MAPKK in plants. Top panel represents the sequence similarity tree of multiple algae and plant lineage. With circle (○) and star (☆) indicate the origin and expansion of receptor families. The timescale (in million year; MYA) of the sequence similarity tree is estimated by TIMETREE. Bottom panel represent the present/absence of MAPKK, MAPKK-II and MAPKK-III members in different algae and plant lineages. Grey box indicates the absence of receptor and green box indicates the presence of receptors in each lineage. The origin of MAPKKs are marked with a circle. *M/C/K represents Mesostigmatophyceae, Chlorokybophyceae and Klebsormidiophyceae. Number of available species from each algae and plant lineages are indicated by numbers in the boxes. Boxplot below represents the percentage (%) and right plot represents the distribution and rug of the relative frequency of the % of MAPKK, MAPKK-II and MAPKK-III members in each lineage. Boxplot elements: centre line, median; bounds of box, 25th and 75th percentiles; whiskers, 1.5 × IQR from 25th and 75th percentiles. n (number of downstream signalling component analyzed) is provided in the 'Protein counts per species' file on *Zenodo* (see Data availability section).

m

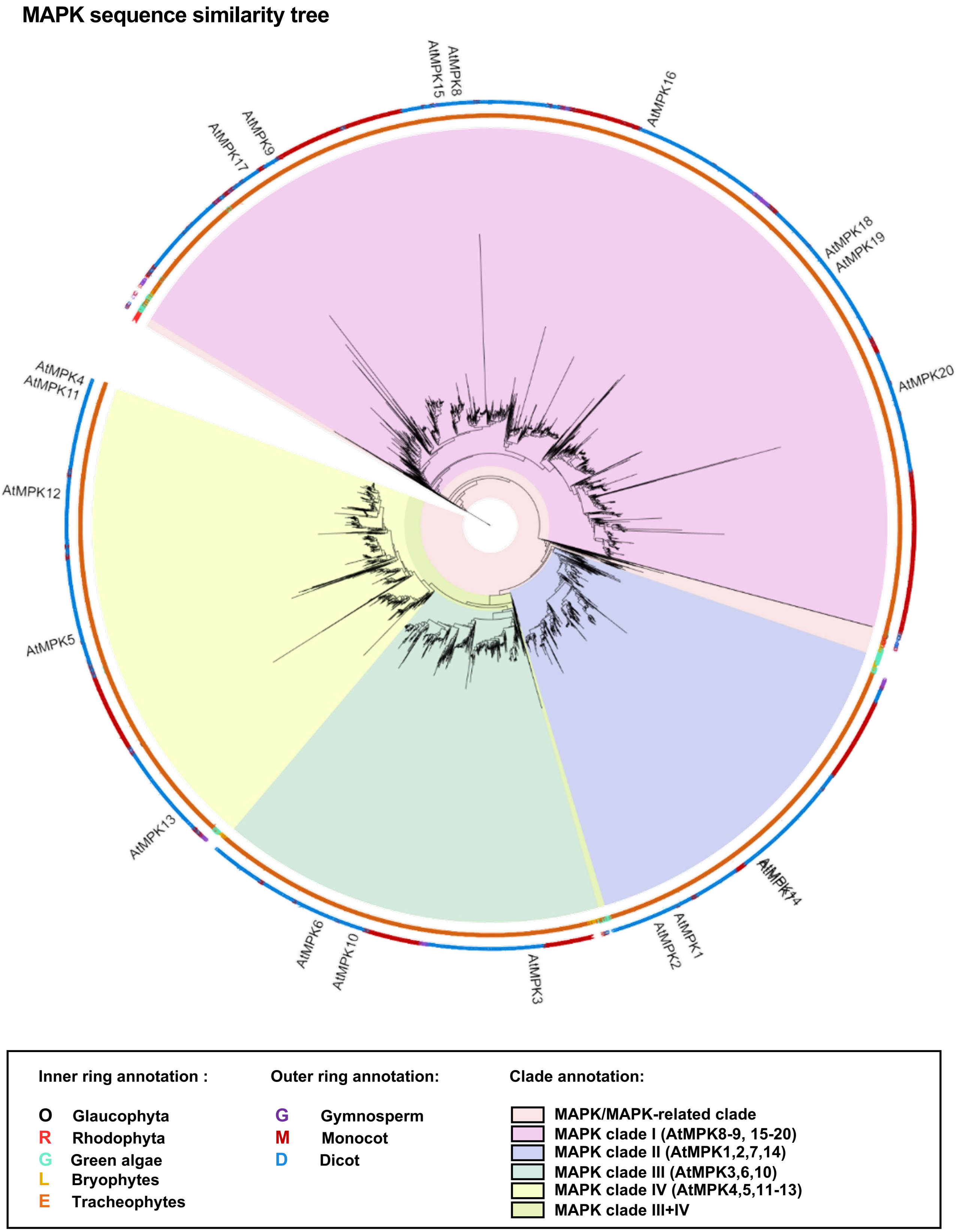

Supplementary figure 8m. Phylogenetic analysis of MAPK in plants. Sequence similarity tree of MAPK members identified from 350 species. The inner ring indicates MAPK members from either Glaucophyta, red algae (Rhodophyta), green algae, Bryophytes or Tracheophytes. The outer ring indicates MAPK members from either gymnosperm, monocots or dicots. The MAPK-I/II/III/IV clades are defined based on previous annotation[104,105]. *Arabidopsis thaliana* MAPKs members are labelled in the tree. Abbreviations for plant species: *A. thaliana*, At.

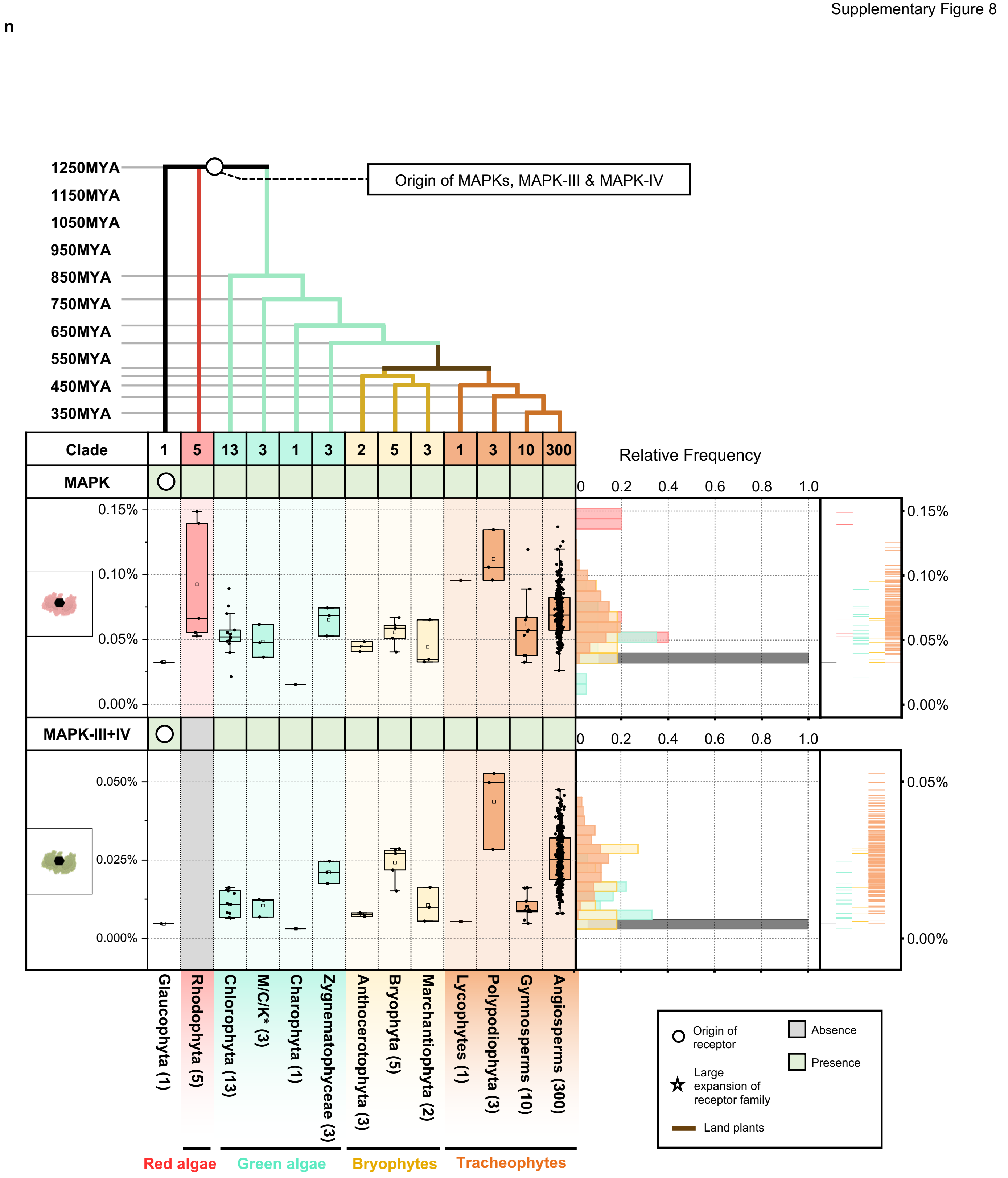

Supplementary figure 8n. The origin and expansion of MAPK in plants. Top panel represents the sequence similarity tree of multiple algae and plant lineage. With circle (○) and star (☆) indicate the origin and expansion of receptor families. The timescale (in million year; MYA) of the sequence similarity tree is estimated by TIMETREE. Bottom panel represent the present/absence of MAPK, MAPK-III+IV members in different algae and plant lineages. Grey box indicates the absence of receptor and green box indicates the presence of receptors in each lineage. The origin of MAPKs are marked with a circle. *M/C/K represents Mesostigmatophyceae, Chlorokybophyceae and Klebsormidiophyceae. Number of available species from each algae and plant lineages are indicated by numbers in the boxes. Boxplot below represents the percentage (%) and right plot represents the distribution and rug of the relative frequency of the % of MAPK, MAPK-III+IV members in each lineage. Boxplot elements: centre line, median; bounds of box, 25th and 75th percentiles; whiskers, 1.5 × IQR from 25th and 75th percentiles. n (number of downstream signalling component analyzed) is provided in the ‘Protein counts per species’ file on *Zenodo* (see Data availability section).

o

CNGC sequence similarity tree

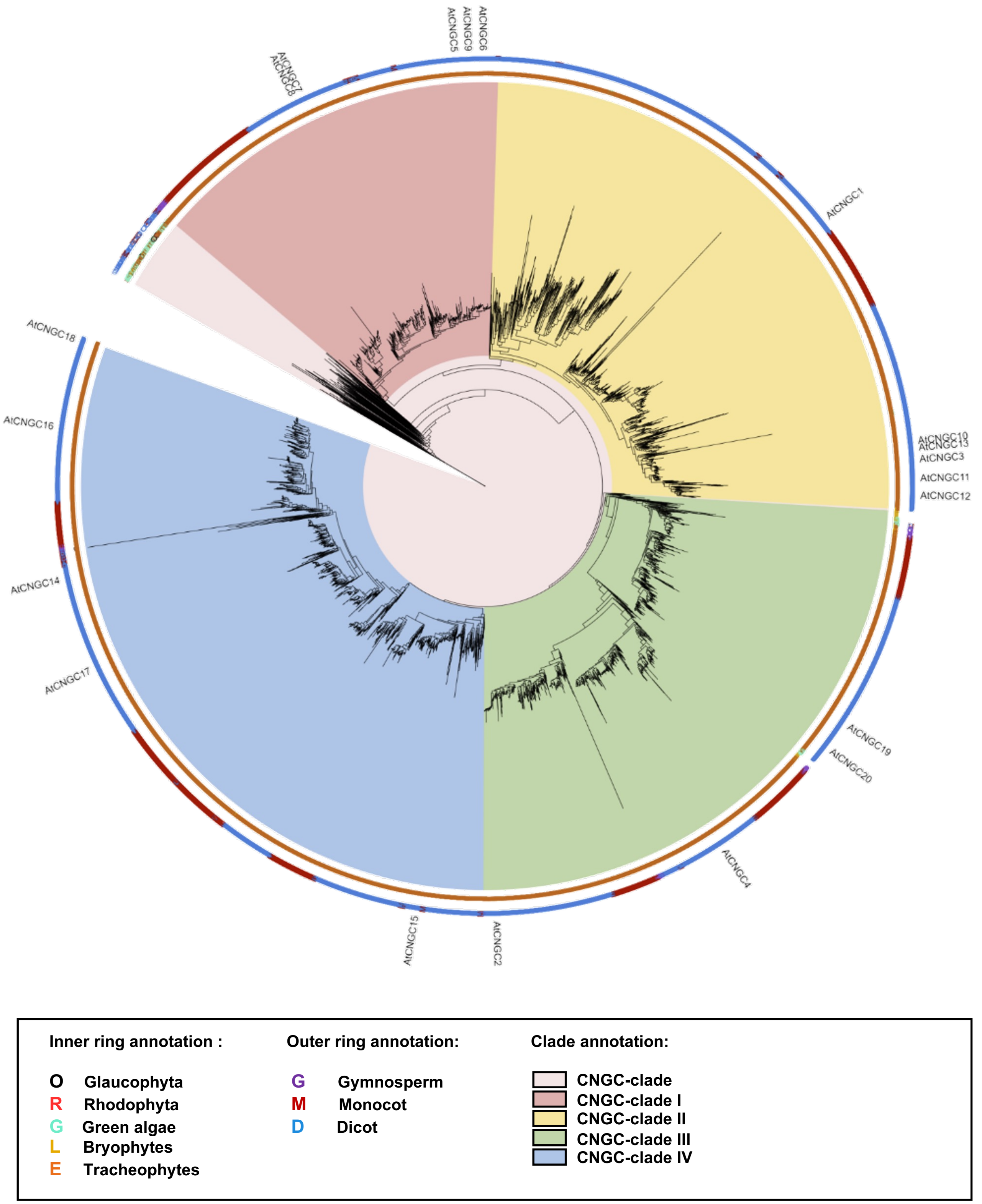

Supplementary figure 8o. Phylogenetic analysis of CNGCs in plants. Sequence similarity tree of CNGCs identified from 350 species. The inner ring indicates CNGC members from either Glaucophyta, red algae (Rhodophyta), green algae, Bryophytes or Tracheophytes. The outer ring indicates CNGC members from either gymnosperm, monocots or dicots. The CNGC-I/II/III/IV clades are defined based on previous annotation[106]. *Arabidopsis thaliana* CNGC members are labelled in the tree. Abbreviations for plant species: *A. thaliana*, At.

p

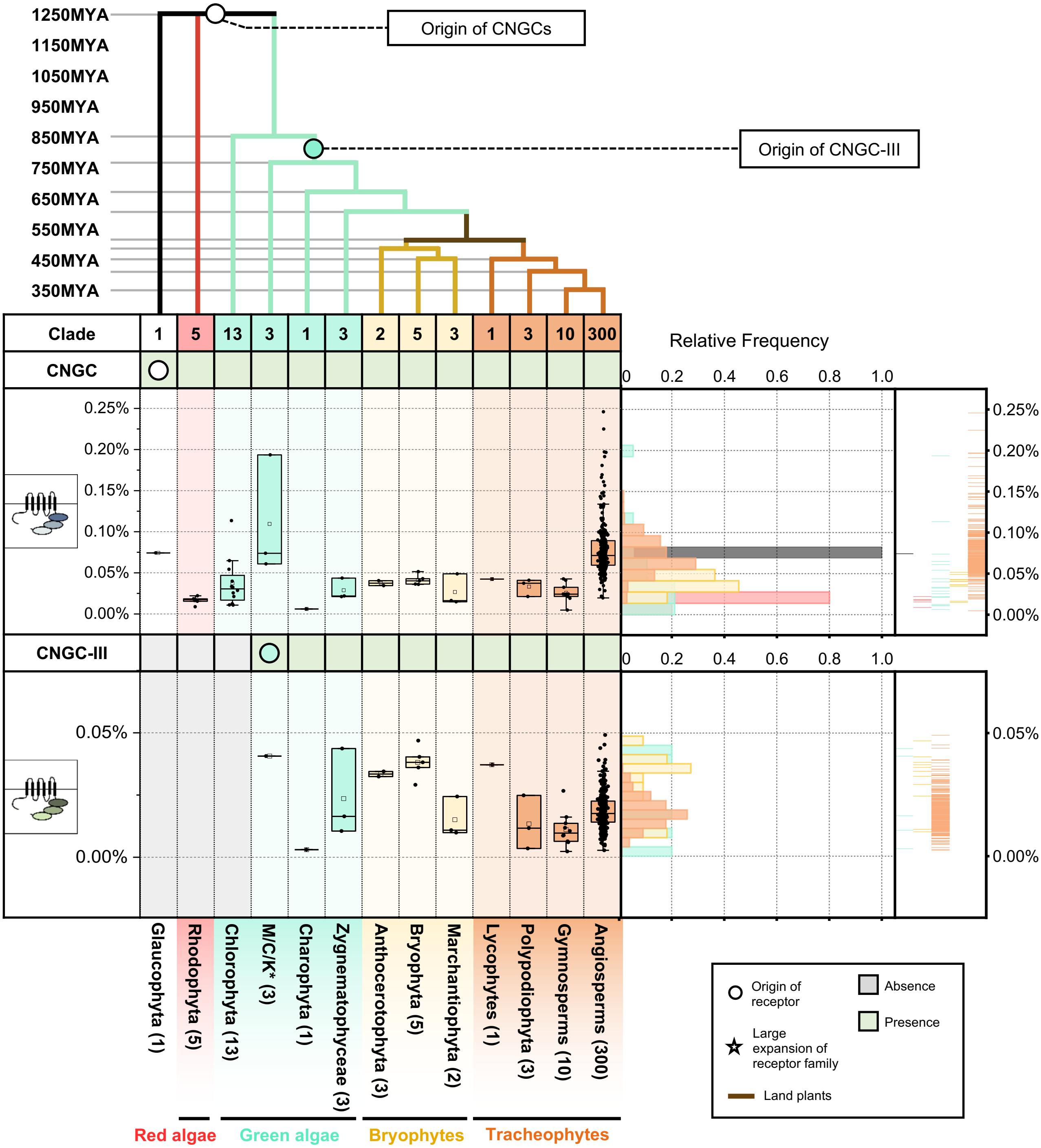

Supplementary figure 8p. The origin and expansion of CNGC in plants. Top panel represents the sequence similarity tree of multiple algae and plant lineage. With circle (○) and star (☆) indicate the origin and expansion of receptor families. The timescale (in million year; MYA) of the sequence similarity tree is estimated by TIMETREE. Bottom panel represent the present/absence of CNGC and CNGC-I members in different algae and plant lineages. Grey box indicates the absence of receptor and green box indicates the presence of receptors in each lineage. The origin of CNGCs are marked with a circle. *M/C/K represents Mesostigmatophyceae, Chlorokybophyceae and Klebsormidiophyceae. Number of available species from each algae and plant lineages are indicated by numbers in the boxes. Boxplot below represents the percentage (%) and right plot represents the distribution and rug of the relative frequency of the % of CNGC and CNGC-I members in each lineage. Boxplot elements: centre line, median; bounds of box, 25th and 75th percentiles; whiskers, 1.5 × IQR from 25th and 75th percentiles. n (number of downstream signalling component analyzed) is provided in the 'Protein counts per species' file on *Zenodo* (see Data availability section).

q

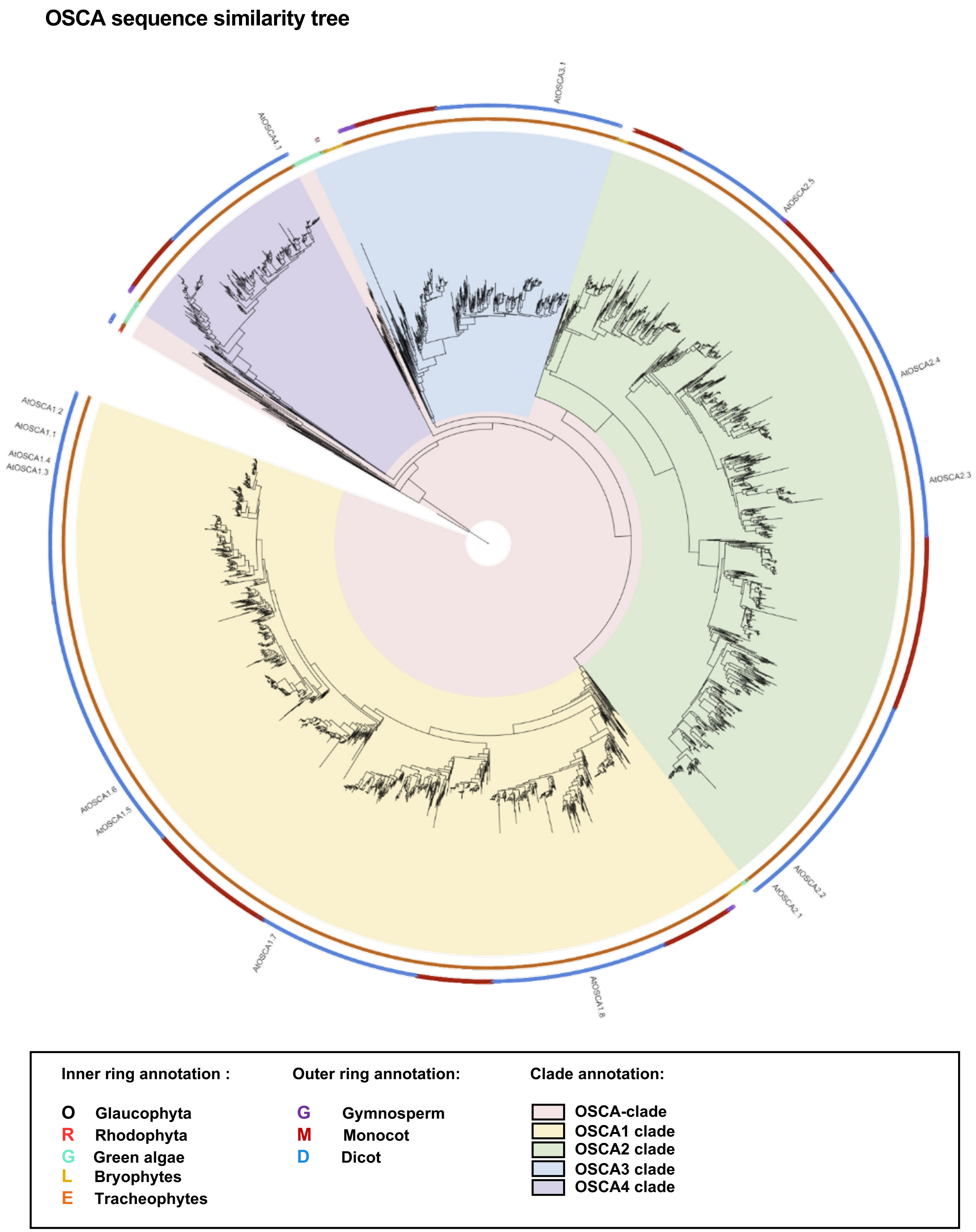

Supplementary figure 8q. Phylogenetic analysis of OSCA in plants. Sequence similarity tree of OSCA members identified from 350 species. The inner ring indicates OSCA members from either Glaucophyta, red algae (Rhodophyta), green algae, Bryophytes or Tracheophytes. The outer ring indicates OSCA members from either gymnosperm, monocots or dicots. The OSCA1/2/3/4 clades are defined based on previous annotation[107]. *Arabidopsis thaliana* OSCAs members are labelled in the tree. Abbreviations for plant species: *A. thaliana*, At.

r

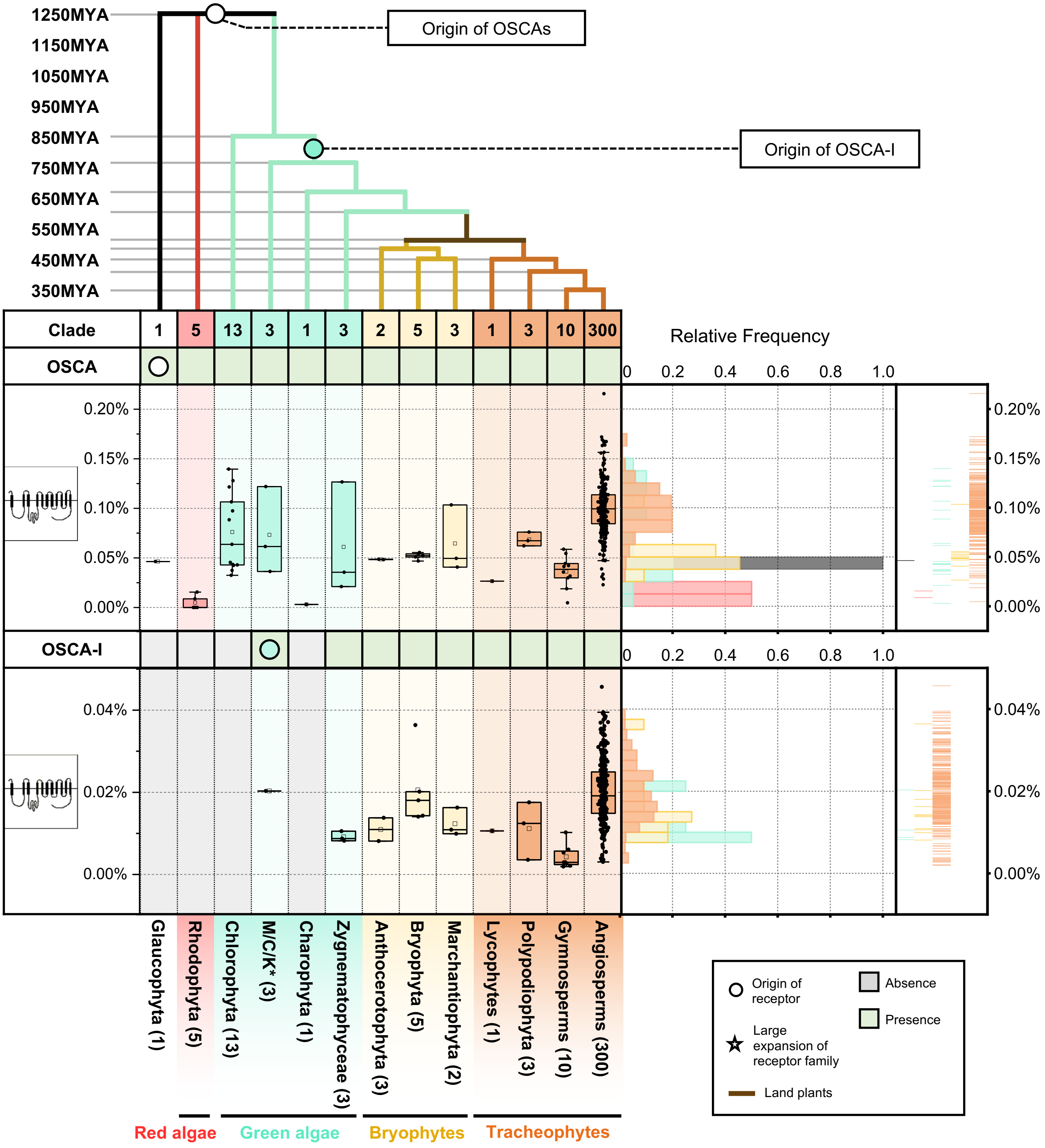

Supplementary figure 8r. The origin and expansion of OSCA in plants. Top panel represents the sequence similarity tree of multiple algae and plant lineage. With circle (○) and star (☆) indicate the origin and expansion of receptor families. The timescale (in million year; MYA) of the sequence similarity tree is estimated by TIMETREE. Bottom panel represent the present/absence of OSCA and OSCA-I members in different algae and plant lineages. Grey box indicates the absence of receptor and green box indicates the presence of receptors in each lineage. The origin of OSCAs are marked with a circle. *M/C/K represents Mesostigmatophyceae, Chlorokybophyceae and Klebsormidiophyceae. Number of available species from each algae and plant lineages are indicated by numbers in the boxes. Boxplot below represents the percentage (%) and right plot represents the distribution and rug of the relative frequency of the % of OSCA and OSCA-I members in each lineage. Boxplot elements: centre line, median; bounds of box, 25th and 75th percentiles; whiskers, 1.5 × IQR from 25th and 75th percentiles. n (number of downstream signalling component analyzed) is provided in the 'Protein counts per species' file on *Zenodo* (see Data availability section).

s

NADPH oxidases sequence similarity tree

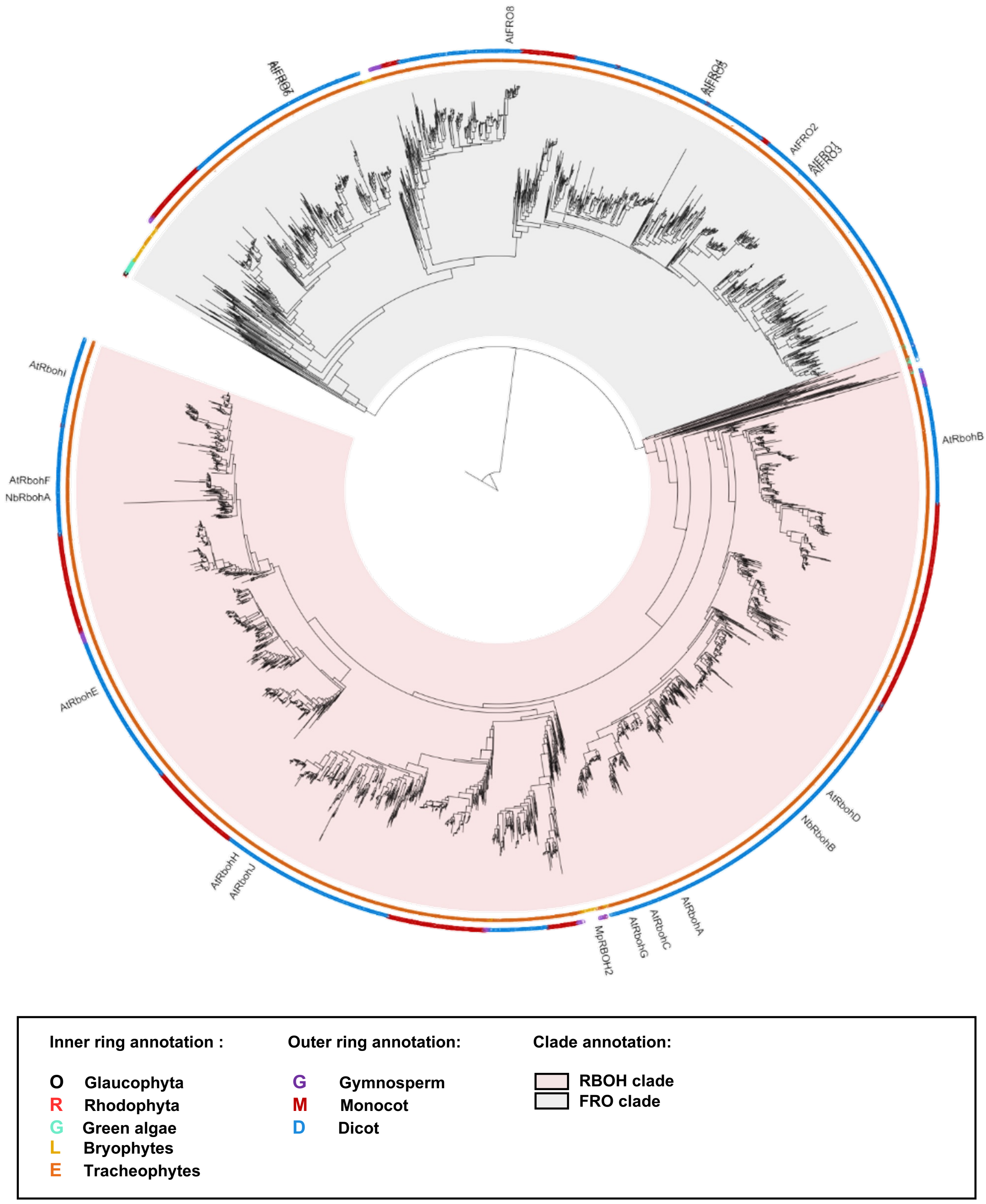

Supplementary figure 8s. Phylogenetic analysis of NADPH oxidases in plants. Sequence similarity tree of NADPH oxidase members identified from 350 species. The inner ring indicates NADPH oxidase members from either Glaucophyta, red algae (Rhodophyta), green algae, Bryophytes or Tracheophytes. The outer ring indicates NADPH oxidase members from either gymnosperm, monocots or dicots. The FRO and RBOH clades are defined. Characterized RBOH members and *Arabidopsis thaliana* NADPH oxidases are labelled in the tree. Abbreviations for plant species: *M. polymorpha*, Mp; *A. thaliana*, At; *N. benthamiana*, Nb.

t

RBOH sequence similarity tree

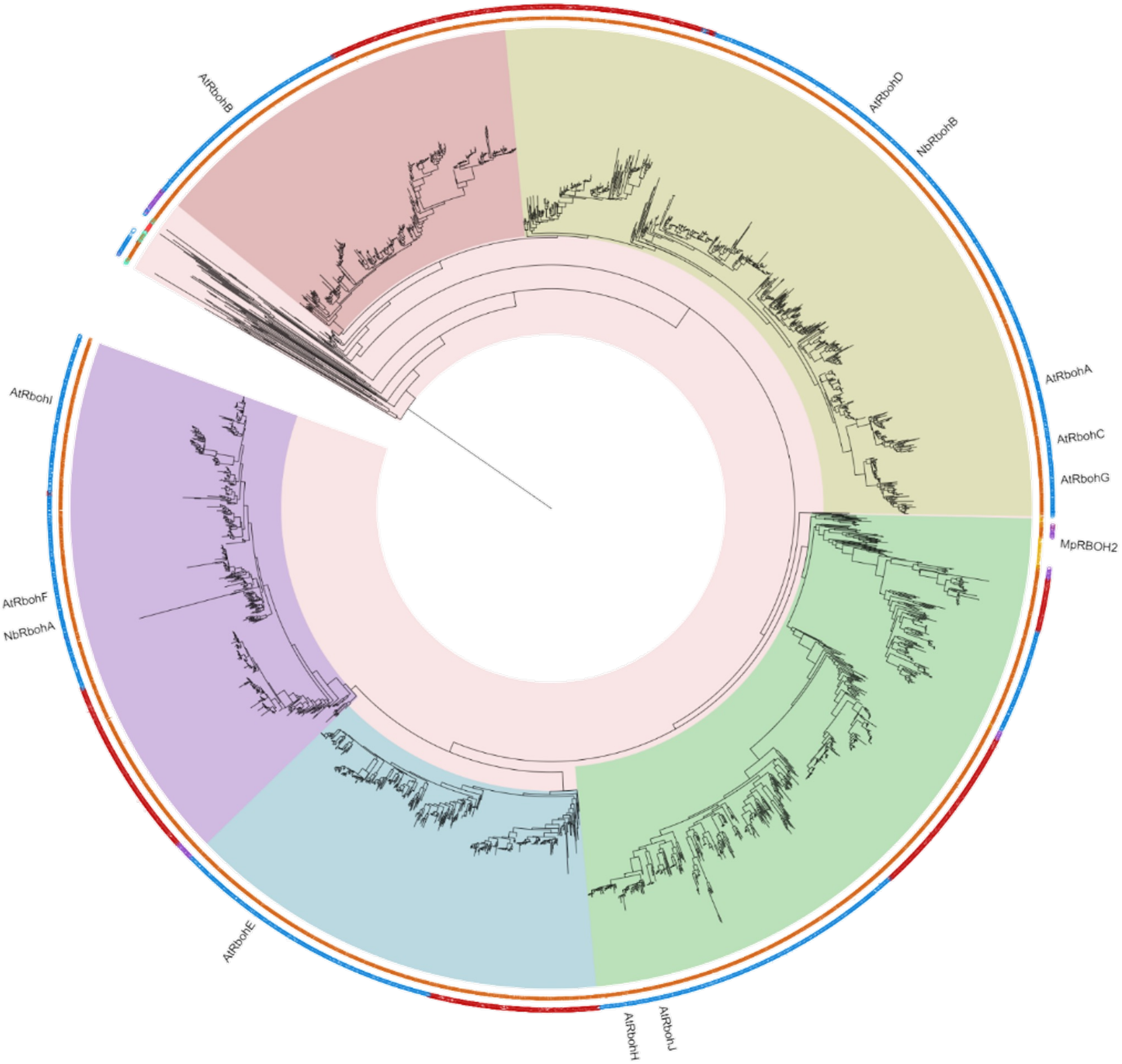

Inner ring annotation :		Outer ring annotation:		Clade annotation:
O	Glaucophyta	G	Gymnosperm	RBOH clade
R	Rhodophyta	M	Monocot	RBOH clade-I
G	Green algae	D	Dicot	RBOH clade-II
L	Bryophytes			RBOH clade-III
E	Tracheophytes			RBOH clade-IV
				RBOH clade-V

Supplementary figure 8t. Phylogenetic analysis of RBOHs in plants. Sequence similarity tree of RBOH members identified from 350 species. The inner ring indicates RBOH members from either Glaucophyta, red algae (Rhodophyta), green algae, Bryophytes or Tracheophytes. The outer ring indicates RBOH members from either gymnosperm, monocots or dicots. The RBOH-I/II/III/IV/V clades are defined based on previous annotation[108,109]. *Arabidopsis thaliana* RBOH members are labelled in the tree. Abbreviations for plant species: *M. polymorpha*, Mp; *A. thaliana*, At; *N. benthamiana*, Nb.

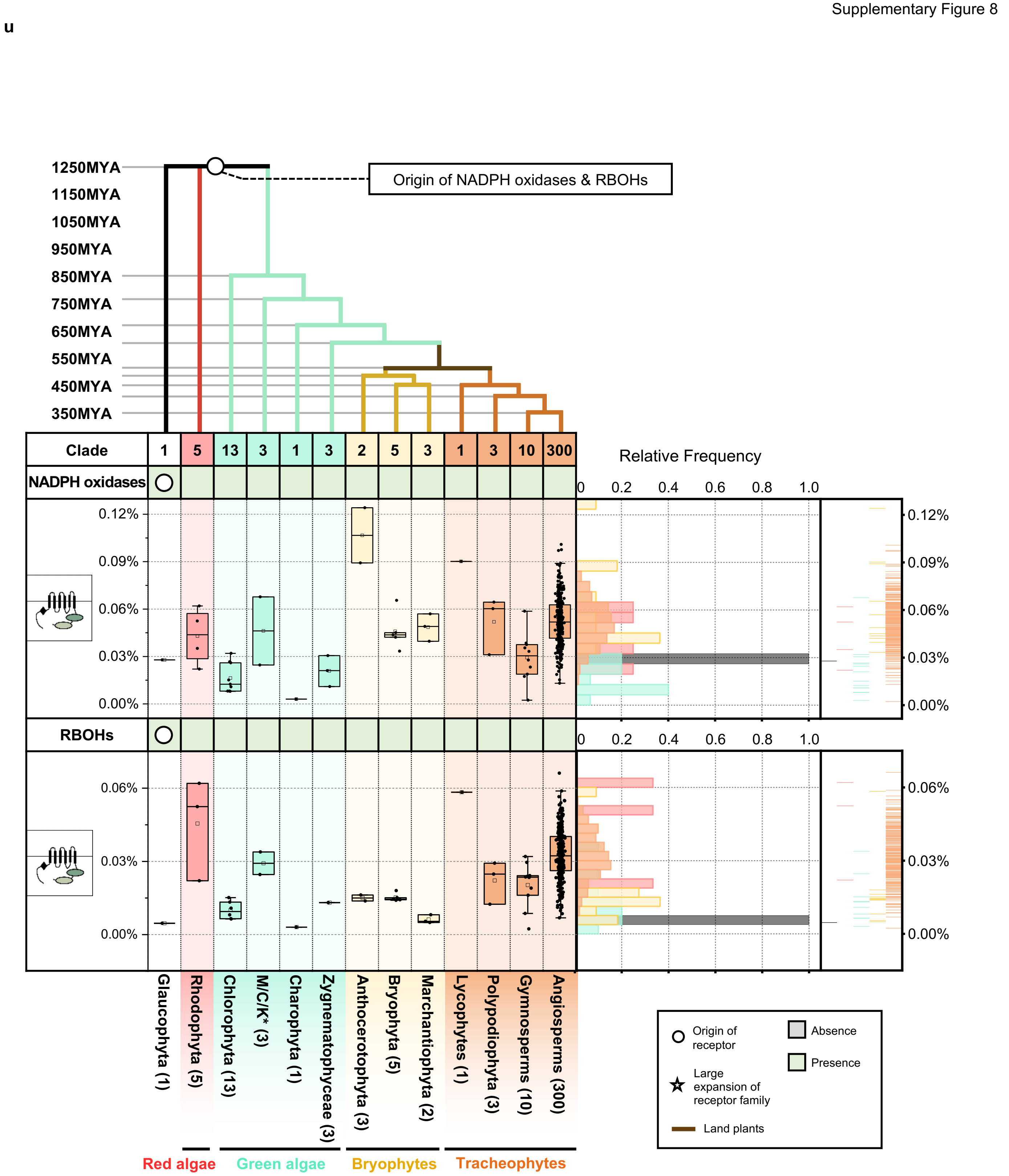

Supplementary figure 8u. The origin and expansion of NADPH oxidases in plants. Top panel represents the sequence similarity tree of multiple algae and plant lineage. With circle (○) and star (☆) indicate the origin and expansion of receptor families. The timescale (in million year; MYA) of the sequence similarity tree is estimated by TIMETREE. Bottom panel represent the present/absence of NADPH oxidase and RBOHs in different algae and plant lineages. Grey box indicates the absence of receptor and green box indicates the presence of receptors in each lineage. The origin of NADPH oxidases are marked with a circle. *M/C/K represents Mesostigmatophyceae, Chlorokybophyceae and Klebsormidiophyceae. Number of available species from each algae and plant lineages are indicated by numbers in the boxes. Boxplot below represents the percentage (%) and right plot represents the distribution and rug of the relative frequency of the % of NADPH oxidase and RBOHs in each lineage. Boxplot elements: centre line, median; bounds of box, 25th and 75th percentiles; whiskers, 1.5 × IQR from 25th and 75th percentiles. n (number of downstream signalling component analyzed) is provided in the 'Protein counts per species' file on *Zenodo* (see Data availability section).

v

EP proteins sequence similarity tree

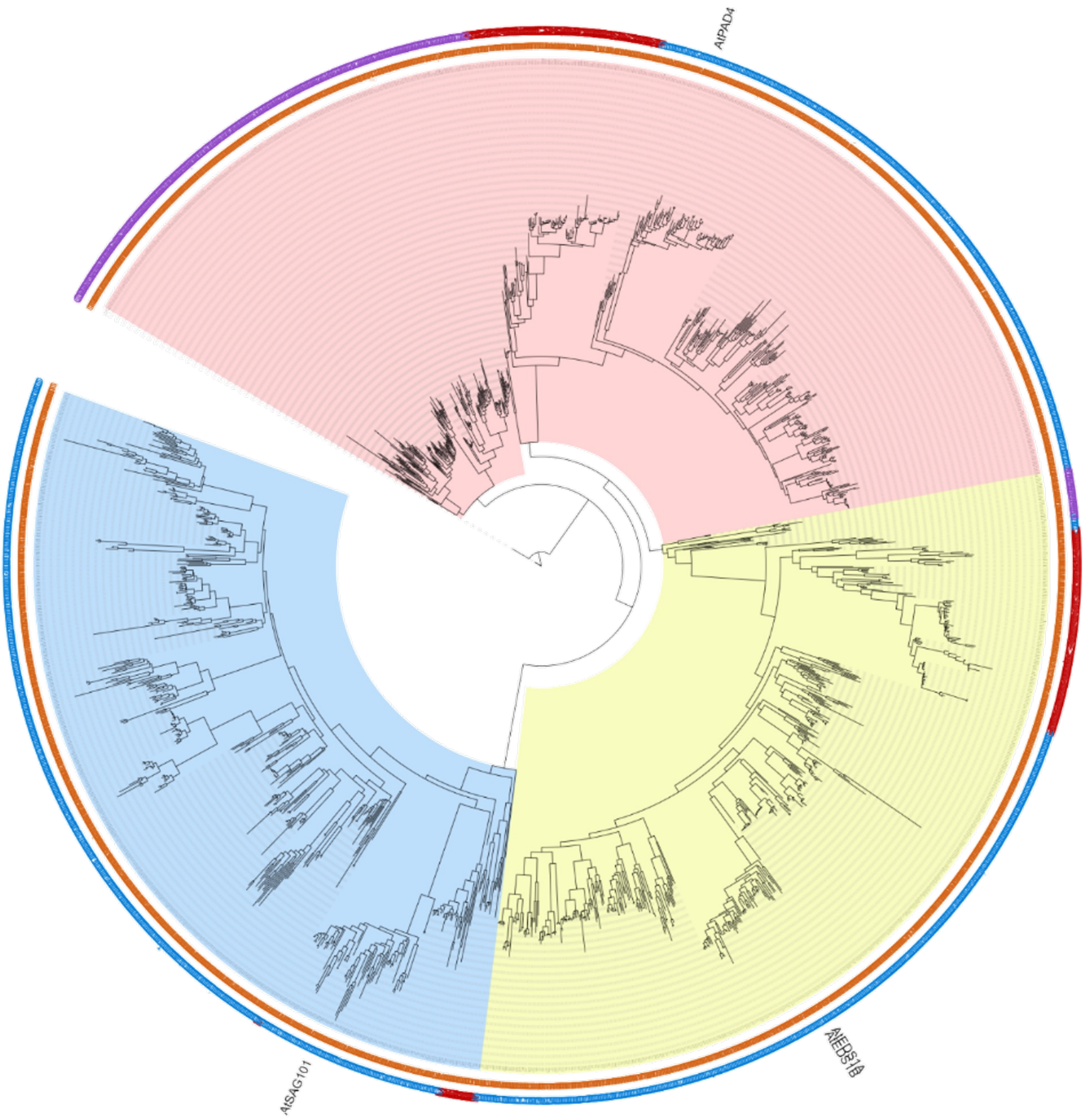

Inner ring annotation :

O Glaucophyta
R Rhodophyta
G Green algae
L Bryophytes
E Tracheophytes

Outer ring annotation:

G Gymnosperm
M Monocot
D Dicot

Clade annotation:

PAD4 clade
EDS1 clade
SAG101 clade

Supplementary figure 8v. Phylogenetic analysis of EP proteins in plants. Sequence similarity tree of EP proteins identified from 350 species. The inner ring indicates EP proteins from either Glaucophyta, red algae (Rhodophyta), green algae, Bryophytes or Tracheophytes. The outer ring indicates EP proteins from either gymnosperm, monocots or dicots. The PAD4, EDS1 and SAG101 clades are defined based on previous annotation[110]. *Arabidopsis thaliana* EP protein members are labelled in the tree. Abbreviations for plant species: *A. thaliana*, At.

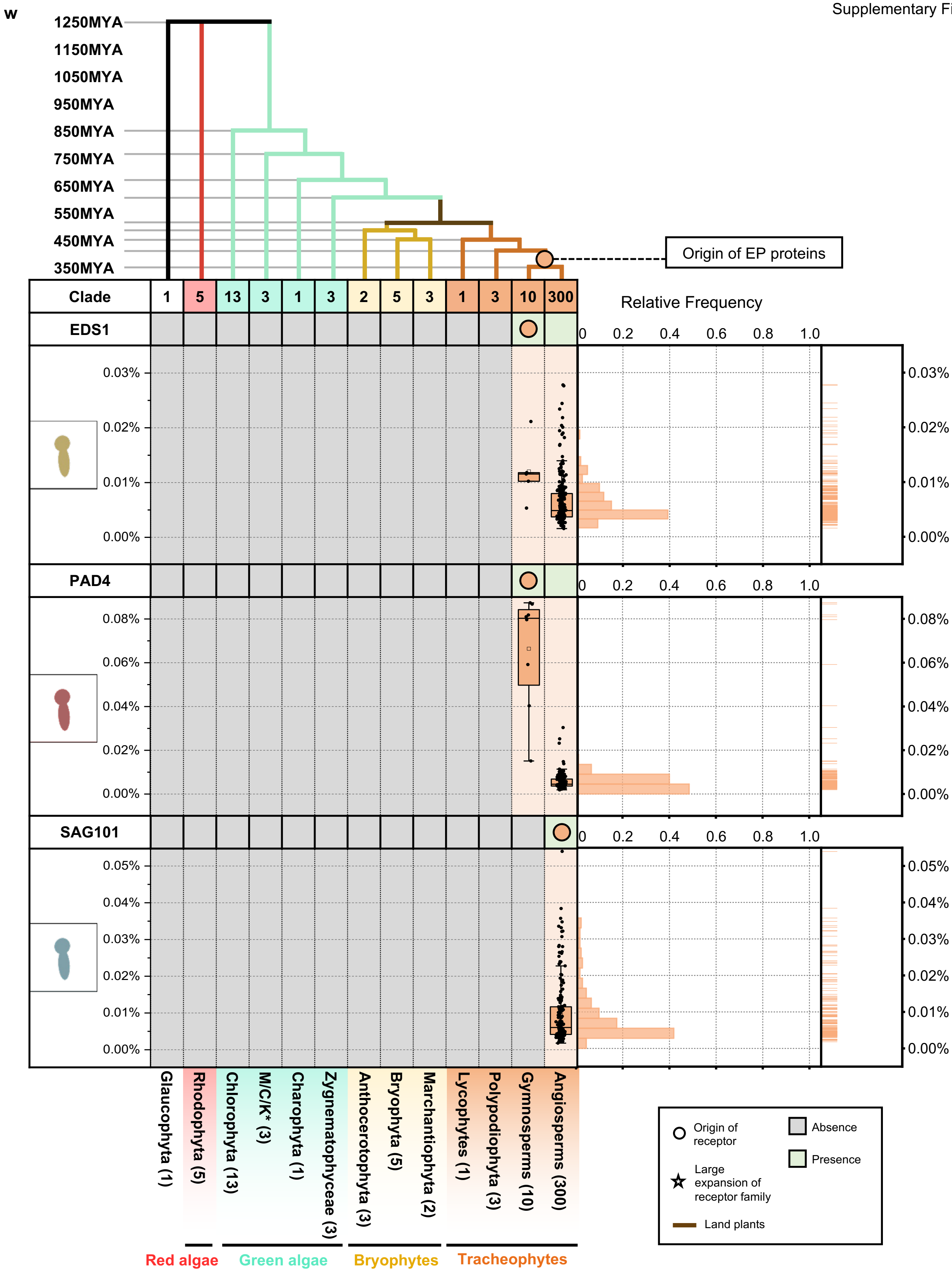

Supplementary figure 8w. The origin and expansion of EP proteins in plants. Top panel represents a sequence similarity tree of multiple algae and plant lineages. Circles (○) and stars (☆) indicate the origin and expansion of receptor families. The timescale (in million years; MYA) of the sequence similarity tree is estimated by TIMETREE. Bottom panel represent the present/absence of EDS1, PAD4 and SAG101 in different algae and plant lineages. Grey box indicates the absence of receptor and green box indicates the presence of EDS1, PAD4 and SAG101 in each lineage. The origin of EP proteins are marked with a circle. *M/C/K represents Mesostigmatophyceae, Chlorokybophyceae and Klebsormidiophyceae. Number of available species from each algae and plant lineages are indicated by numbers in the boxes. Boxplot below represents the percentage (%) and right plot represents the distribution and rug of the relative frequency of the % of EDS1, PAD4 and SAG101 in each lineage. Boxplot elements: centre line, median; bounds of box, 25th and 75th percentiles; whiskers, 1.5 × IQR from 25th and 75th percentiles. n (number of downstream signalling component analyzed) is provided in the 'Protein counts per species' file on *Zenodo* (see Data availability section).

x

RWP8-NLR sequence similarity tree

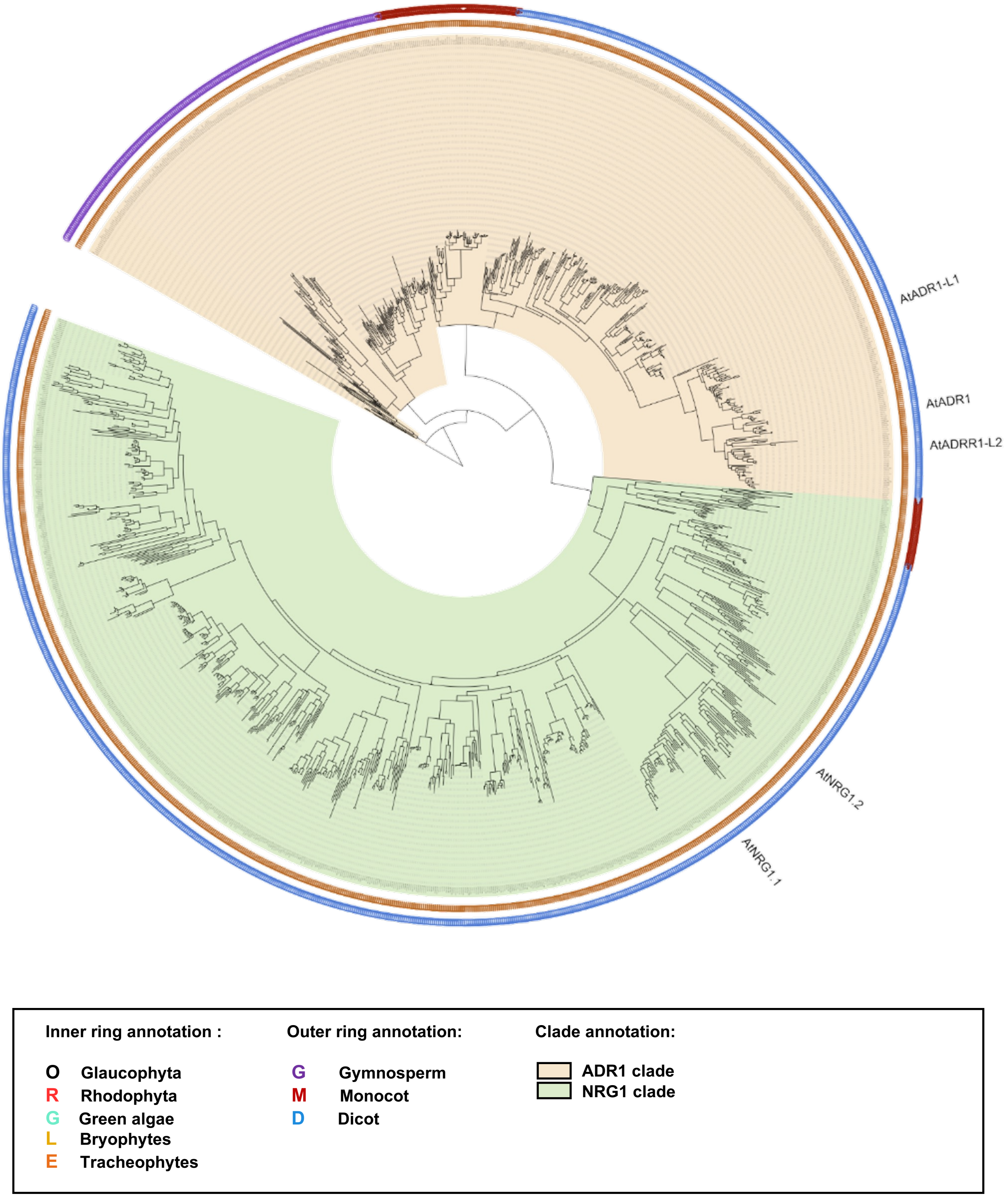

Supplementary figure 8x. Phylogenetic analysis of RPW8-NLRs (RNLs/helper NLRs) in plants. Sequence similarity tree of helper NLRs identified from 350 species. The inner ring indicates helper NLRs from either Glaucophyta, red algae (Rhodophyta), green algae, Bryophytes or Tracheophytes. The outer ring indicates helper NLRs from either gymnosperm, monocots or dicots. The ADR1 and NRG1 clades are defined based on previous annotations[111]. *Arabidopsis thaliana* helper NLRs are labelled in the tree. Abbreviations for plant species: *A. thaliana*, At.

y

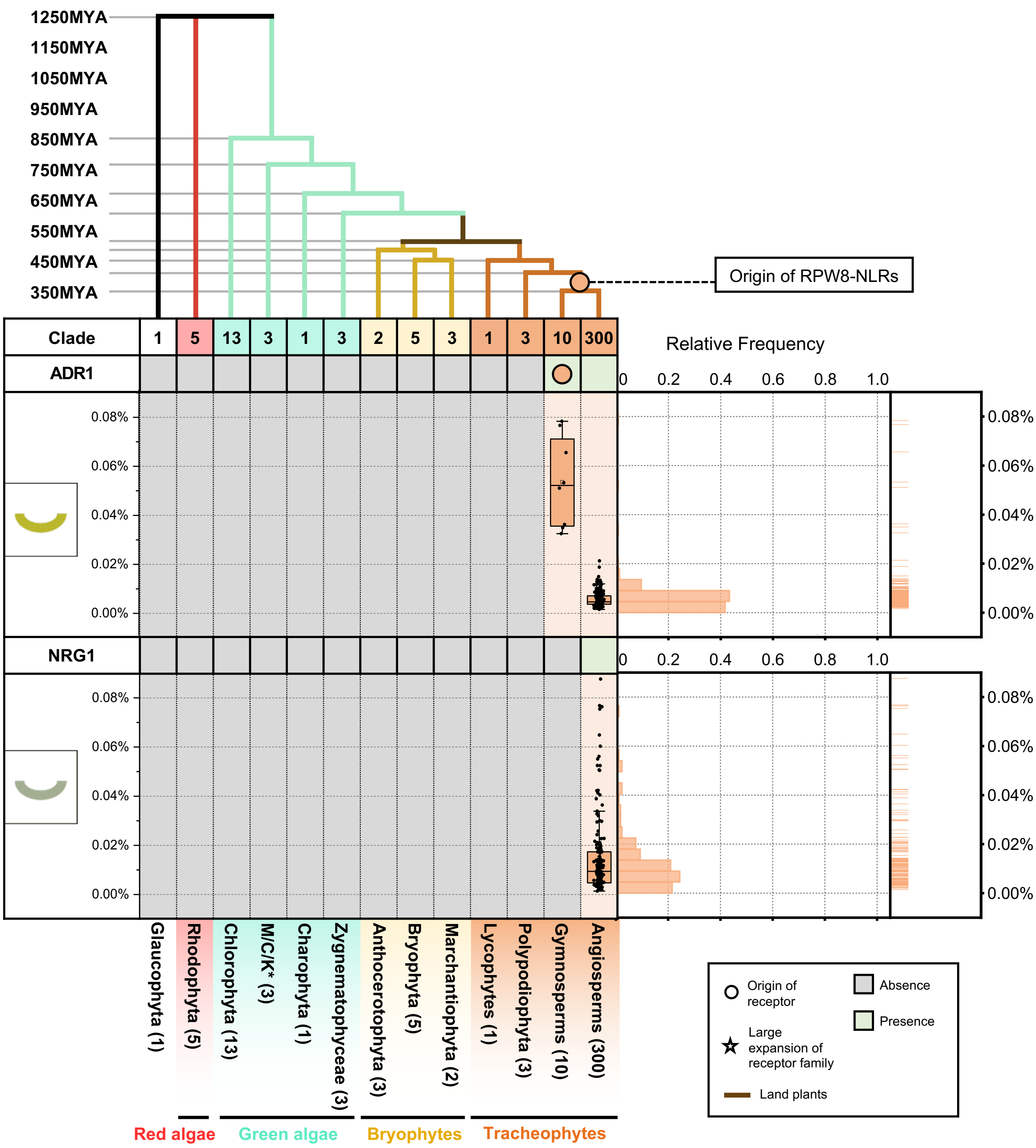

Supplementary figure 8y. The origin and expansion of RWP8-NLRs in plants. Top panel represents a sequence similarity tree of multiple algae and plant lineages. Circles (○) and stars (☆) indicate the origin and expansion of receptor families, respectively. The timescale (in million years; MYA) of the sequence similarity tree was estimated by TIMETREE. Bottom panel represents the presence or absence of RPW8-NLRs in different algal and plant lineages. Grey box indicates the absence of receptors and green box indicates the presence of receptors in each lineage. The origin of RWP8-NLRs is marked with a circle. *M/C/K represents Mesostigmatophyceae, Chlorokybophyceae and Klebsormidiophyceae. Number of available species from algae and plant lineages are indicated by numbers in the boxes. Boxplot below represents the percentage (%) and right plot represents the distribution and rug of the relative frequency of the % RPW8-NLRs in each lineage. Boxplot elements: centre line, median; bounds of box, 25th and 75th percentiles; whiskers, 1.5 × IQR from 25th and 75th percentiles. n (number of downstream signalling component analyzed) is provided in the 'Protein counts per species' file on *Zenodo* (see Data availability section).

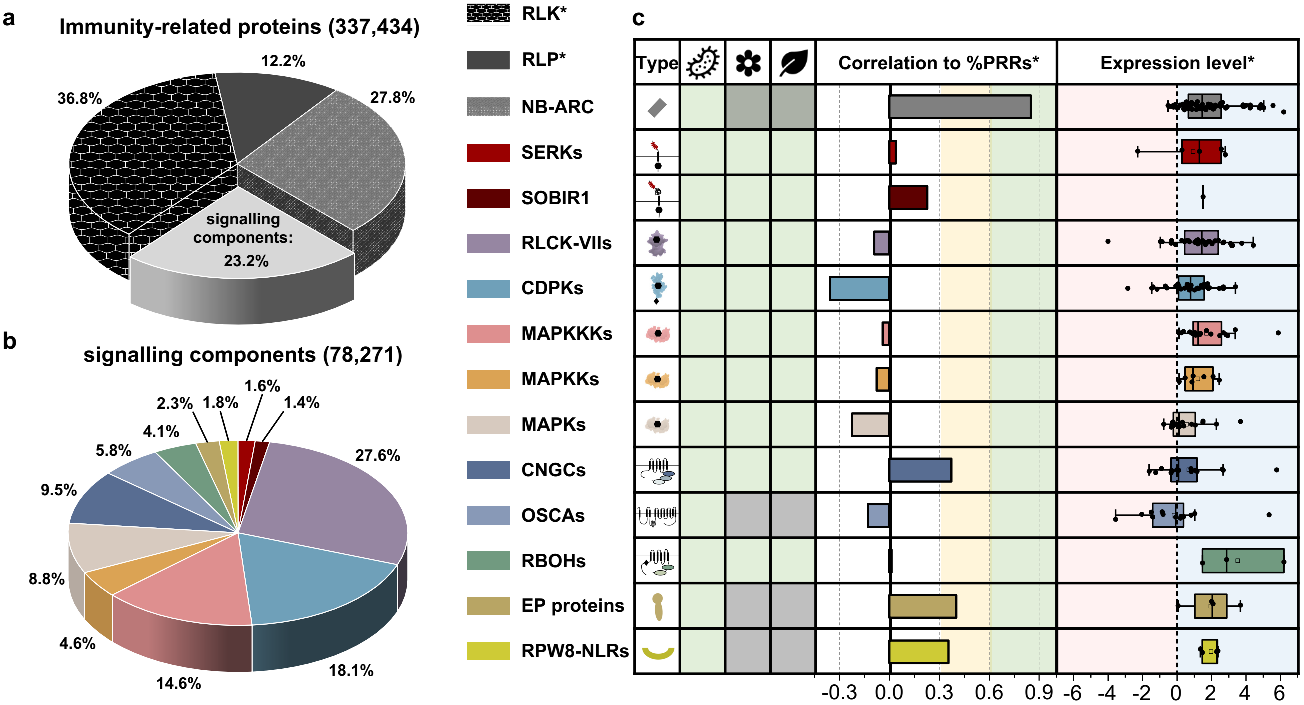
a
Immunity-related proteins (337,434)
12.2%
36.8%
27.8%
signalling components:
23.2%
b
signalling components (78,271)
1.6%
2.3%
1.8%
1.4%
4.1%
5.8%
27.6%
9.5%
8.8%
4.6%
14.6%
18.1%
RLK*
RLP*
NB-ARC
SERKs
SOBIR1
RLCK-VIIs
CDPKs
MAPKKKs
MAPKKs
MAPKs
CNGCs
OSCAs
RBOHs
EP proteins
RPW8-NLRs
c
Type
Correlation to %PRRs*
Expression level*
-0.3 0.0 0.3 0.6 0.9
-6 -4 -2 0 2 4 6

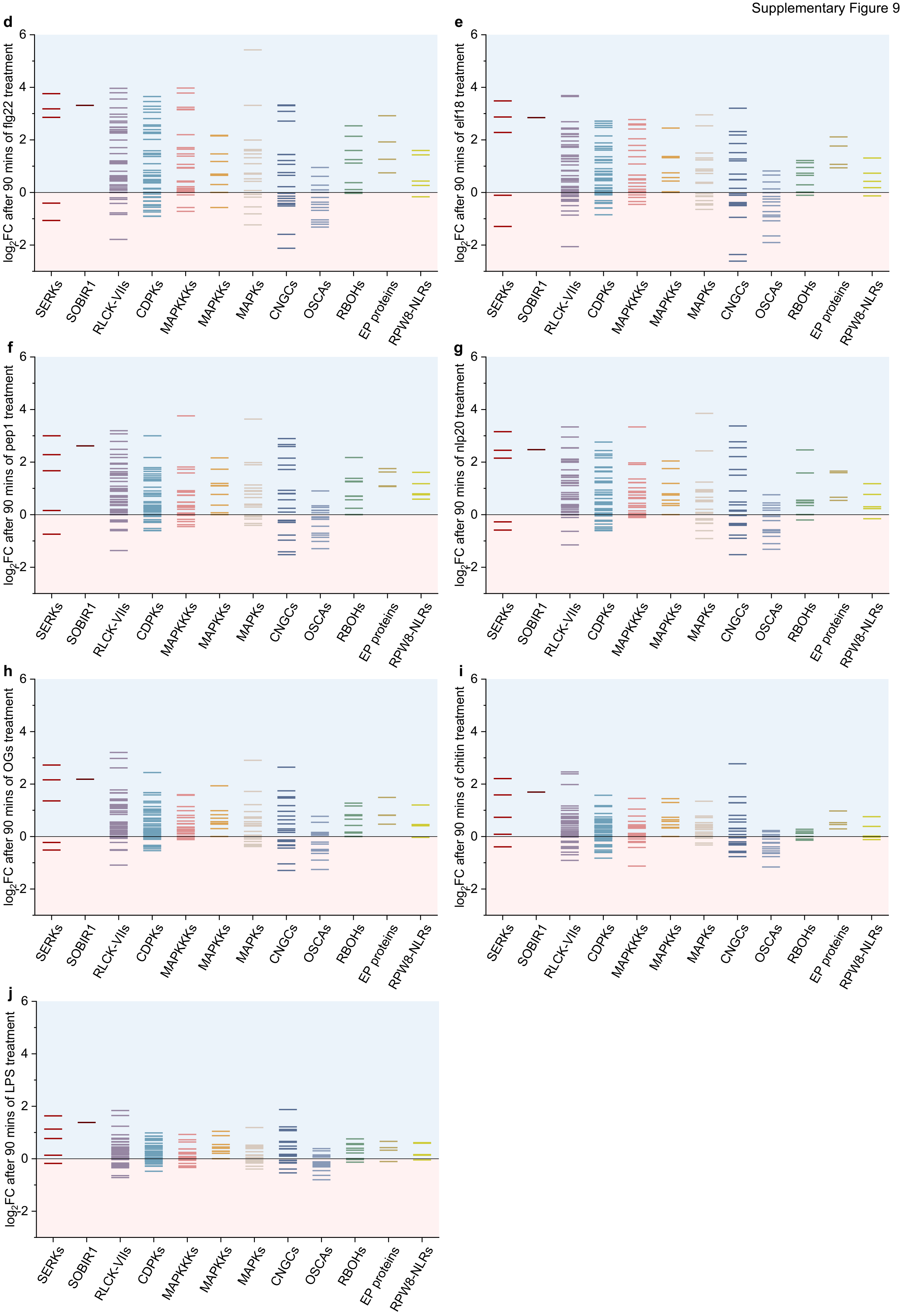
d
log2FC after 90 mins of flg22 treatment
e
log2FC after 90 mins of elf18 treatment
f
log2FC after 90 mins of pep1 treatment
g
log2FC after 90 mins of nlp20 treatment
h
log2FC after 90 mins of OGs treatment
i
log2FC after 90 mins of chitin treatment
j
log2FC after 90 mins of LPS treatment
6
4
2
0
-2
SERKs
SOBIR1
RLCK-VIIs
CDPKs
MAPKKKs
MAPKKs
MAPKs
CNGCs
OSCAs
RBOHs
EP proteins
RPW8-NLRs

Supplementary figure 9. Properties and expressions of cell-surface receptor downstream signalling components (a) Distribution of RLKs, RLPs, NB-ARCs, and PTI-signalling components in plants. Each fraction represents the percentage (%) of ectodomain out of all four protein classes from 350 species (337,434). *Note that RLKs and RLPs here represent RLKs and RLPs with LRR-, G-lectin-, L-lectin-, LysM-, Malectin-, WAK- and Duf26- ectodomains. **(b)** Distribution of PTI-signalling components in plants. Each fraction represents the percentage (%) of each protein family out of all the families combined (78,271). The colour codes for **(a)** and **(b)** are indicated on the right. **(c)** Characterised PTI-signalling components involved in microbial interaction (bacteria icon), reproduction (flower icon), and development (leaf icon) are indicated with green boxes. A grey box indicates that receptor class has not been reported to be involved in that particular biological process (see Supplementary figure 7). The sizes of each signalling component family are given as a percent of all the proteins in the genomes of the plant species. Correlations between different classes of signalling components and PRR* in 300 angiosperms are indicated by the lengths of bars. *Note that PRRs here represent the combined sum of LRR-RLK-XII and LRR-RLPs, since many characterised members of these two classes are involved in PAMP recognition. A moderate positive correlation is represented in yellow (Pearson's r between 0.3-0.6). Expression level* refers to the expression of each class of PTI-signalling component during NTI in *A. thaliana*. The blue shade represents increased expression, and the red shade represents decreased expression during NTI. The X-axis represents $\log_2$ (fold change during NTI relative to untreated samples). The boxplot elements: centre line, median; bounds of box, 25th and 75th percentiles; whiskers, 1.5 × IQR from 25th and 75th percentiles. RNA-seq data analysed here were reported previously in reference 16 in main text, where NTI was activated by estradiol-induced expression of *AvrRps4* in *A. thaliana* for 4 hours. **(d-j)** The expression of PTI signalling components during PTI in *Arabidopsis thaliana*. *Arabidopsis thaliana* seedlings were treated with (d) flg22, (e) elf18, (f) pep1, (g) nlp20, (h) OGs, (i) chitin, or (j) LPS to activate PTI. Light blue represents increased expression and light pink represents decreased expression during PTI. X-axis values represent log2 (fold change during PTI relative to samples at 0 min after treatment). RNA-seq data analyzed here were reported previously, where PTI was activated by different PAMPs/DAMPs in *A. thaliana* for 90mins. RNA-seq data were obtained from Bjornson et al, *Nature Plants* 2021 (reference 15 in main text). For **c-j**, n (number of downstream signalling component analyzed in the RNAseq data) is provided in the Source Data file.

a

LRR-RLP	Biological process			Number of LRR motifs	N-loopout domain (NL)				Island domain (ID)			
					NL	Size of NL (a.a.)	Position (N to C)	Position (C to N)	ID	Size of ID (a.a.)	Position (N to C)	Position (C to N)
AtRLP1				33	1	10	1	32	1	71	29	4
AtRLP23				28	0	-	-	-	1	49	24	4
AtRLP30				23	1	6	-1	-	1	53	19	4
AtRLP32				26	1	11	-1	-	1	47	22	4
AtRLP42				28	0	-	-	-	1	50	24	4
NbCSPR				32	1	7	1	31	1	52	28	4
NbRXEG1				32	1	15	1	31	1	53	28	4
SlCf-2				31	0	-	-	-	1	42	27	4
SlCf-4				24	1	13	1	23	1	47	20	4
SlCf-5				31	0	-	-	-	1	41	27	4
SlCf-9				26	1	22	-1	-	1	46	22	4
SlEIX2				32	1	15	1	31	1	51	28	4
SlVe1				34	1	10	4	30	1	52	30	4
SlI				32	1	24	1	31	1	73	28	4
SmELR				35	1	14	-1	-	1	51	31	4
BnRLM2				29	1	7	-1	-	1	46	25	4
SlCuRe1				35	2	8 & 13	1 & 4	34 & 31	1	74	31	4
VnINR				27	1	22	1	26	1	46	23	4
NbREL				30	2	30 & 11	1 & 4	29 & 26	1	41	26	4
AtCLV2				22	0	-	-	-	1	43	18	4
AtTMM				13	0	-	-	-	0	-	-	-

b

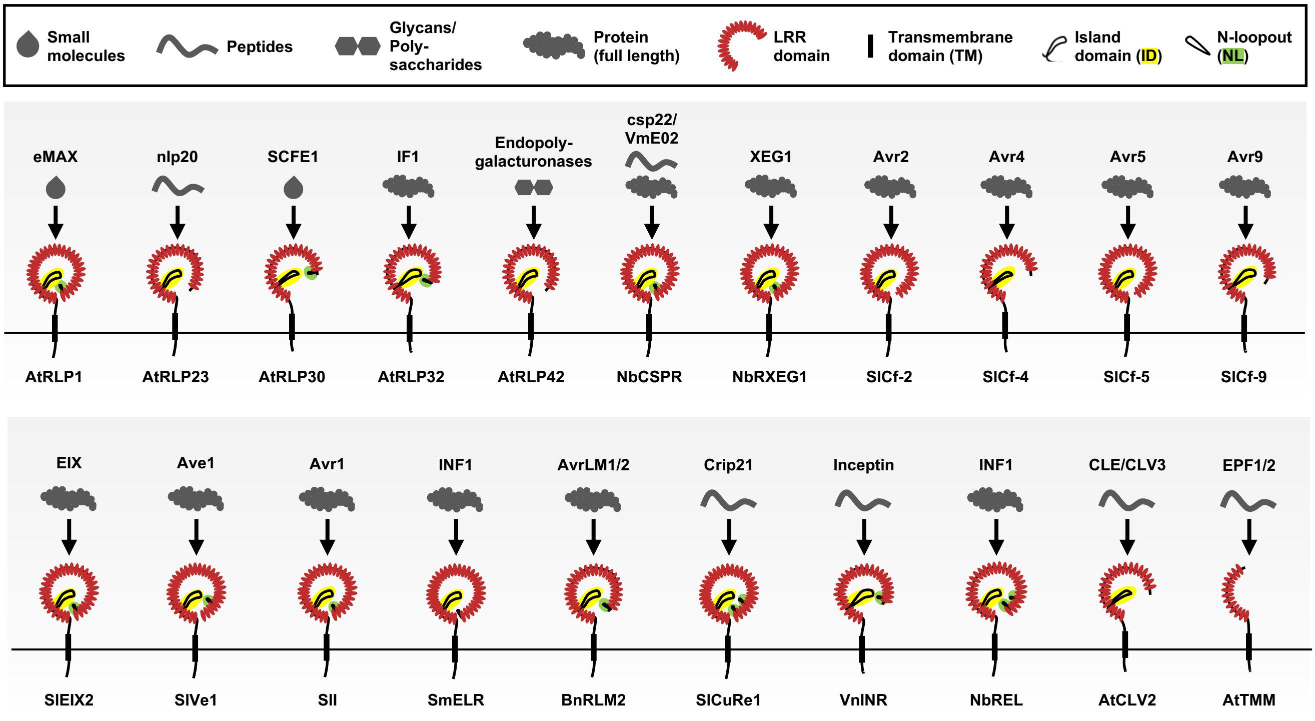

Supplementary figure 10. Domain architecture of the characterized LRR-RLP in plants. (a) Table represents the domain architecture of the characterized LRR-RLPs in plants. Characterized receptors involved in microbial interaction (bacteria icon), reproduction (flower icon) and development (leaf icon) are indicated with green boxes. Grey box indicates that receptor class has not been reported to involved in that biological process. Number of LRR motifs, N-loopouts (NLs) and island domains (IDs), and the position of NL/ID (from N-to-C terminal or C-to-N terminal) are obtained from structures shown in supplementary figure 11. (b) Schematic figure represents the domain architecture of the characterized LRR-RLPs in plants. Arrow represents the ligands of which these receptor classes have been reported to perceive or recognize. Yellow highlights represent IDs and green highlights represent NLs. Upper box defines the ligands recognized by different LRR-RLPs and the domains in the LRR-RLPs. Number of LRRs and position of ID/NL are obtained from structures shown in supplementary figure 11. Note that these receptors might be able to recognise other unidentified ligands. References to the genes are included in the Supplementary Reference.

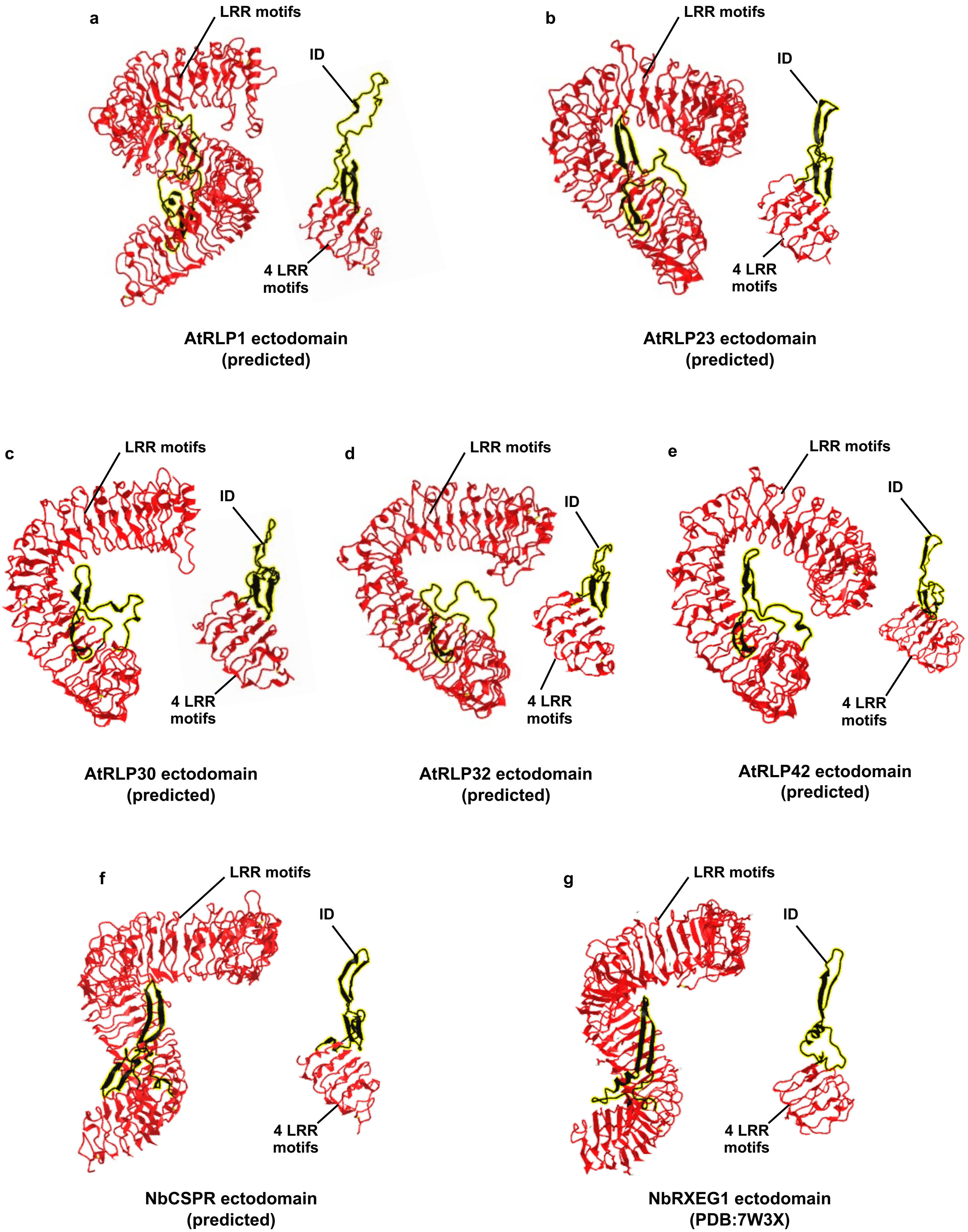
a
LRR motifs
ID
4 LRR motifs
AtRLP1 ectodomain (predicted)
b
LRR motifs
ID
4 LRR motifs
AtRLP23 ectodomain (predicted)
c
LRR motifs
ID
4 LRR motifs
AtRLP30 ectodomain (predicted)
d
LRR motifs
ID
4 LRR motifs
AtRLP32 ectodomain (predicted)
e
LRR motifs
ID
4 LRR motifs
AtRLP42 ectodomain (predicted)
f
LRR motifs
ID
4 LRR motifs
NbCSPR ectodomain (predicted)
g
LRR motifs
ID
4 LRR motifs
NbRXEG1 ectodomain (PDB:7W3X)

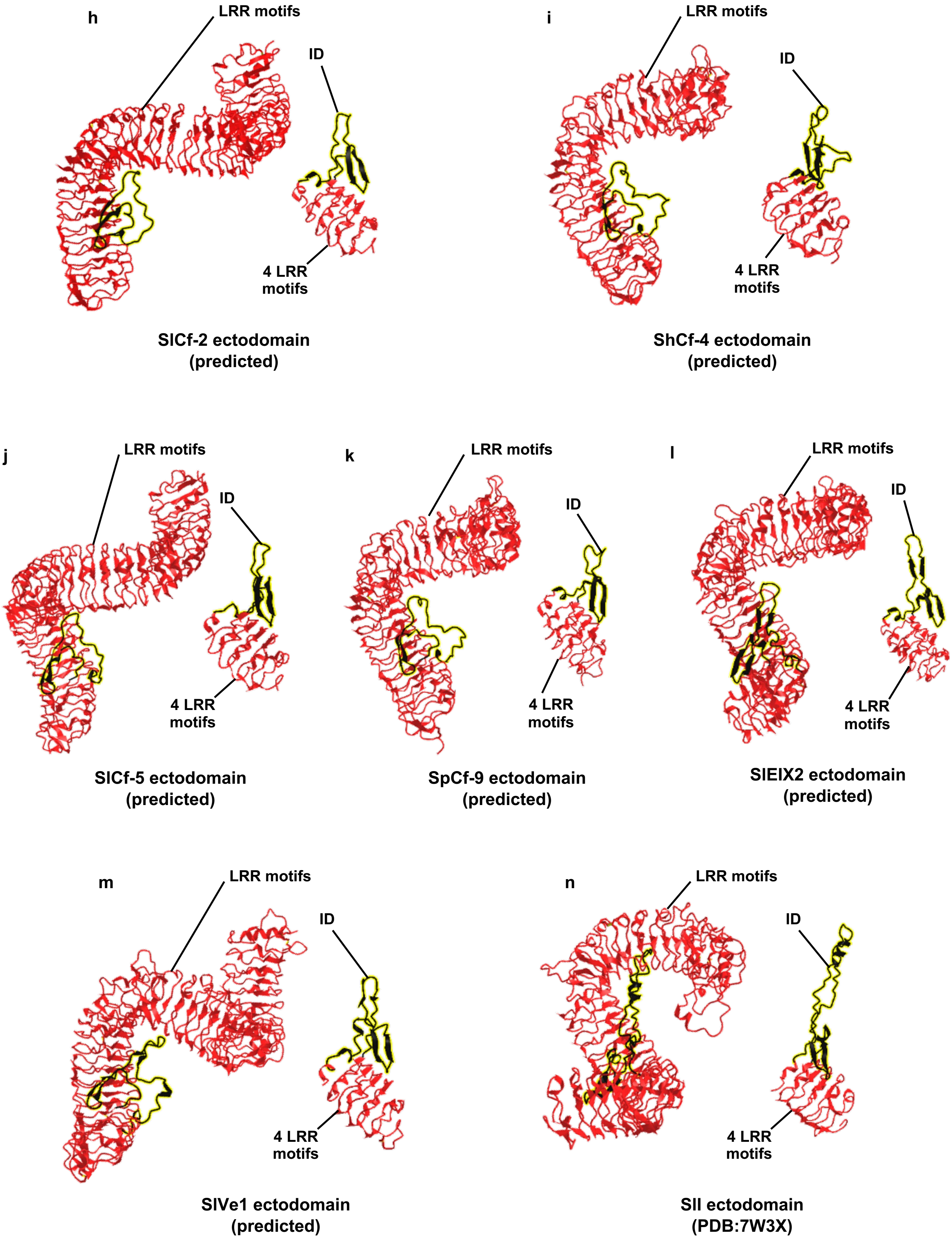
h
LRR motifs
ID
4 LRR motifs
SlCf-2 ectodomain (predicted)
i
LRR motifs
ID
4 LRR motifs
ShCf-4 ectodomain (predicted)
j
LRR motifs
ID
4 LRR motifs
SlCf-5 ectodomain (predicted)
k
LRR motifs
ID
4 LRR motifs
SpCf-9 ectodomain (predicted)
l
LRR motifs
ID
4 LRR motifs
SlEIX2 ectodomain (predicted)
m
LRR motifs
ID
4 LRR motifs
SlVe1 ectodomain (predicted)
n
LRR motifs
ID
4 LRR motifs
SlI ectodomain (PDB:7W3X)

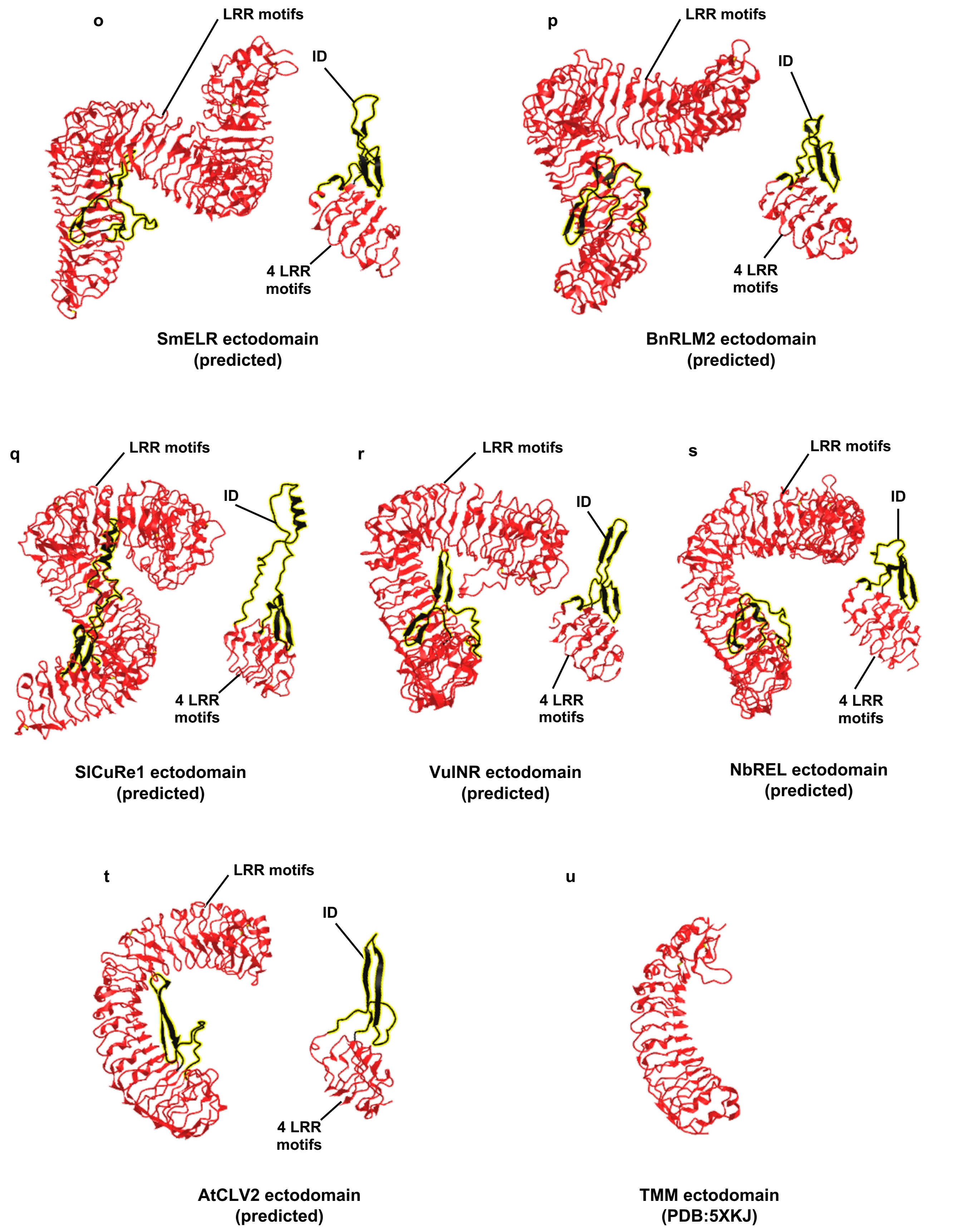

Supplementary figure 11. Published or predicted structures of ectodomains of LRR-RLP in plants. Ectodomain structures of characterized LRR-RLPs in plants. Structure of RXEG1 and TMM were obtained from PDB[112.113]. Structures of the other LRR-RLPs were predicted by Alphafold2[*,114.] Ectodomains were trimmed by PDBeditor and visualized in iCn3D[115]. Island domains (ID) are highlighted in black with yellow background.

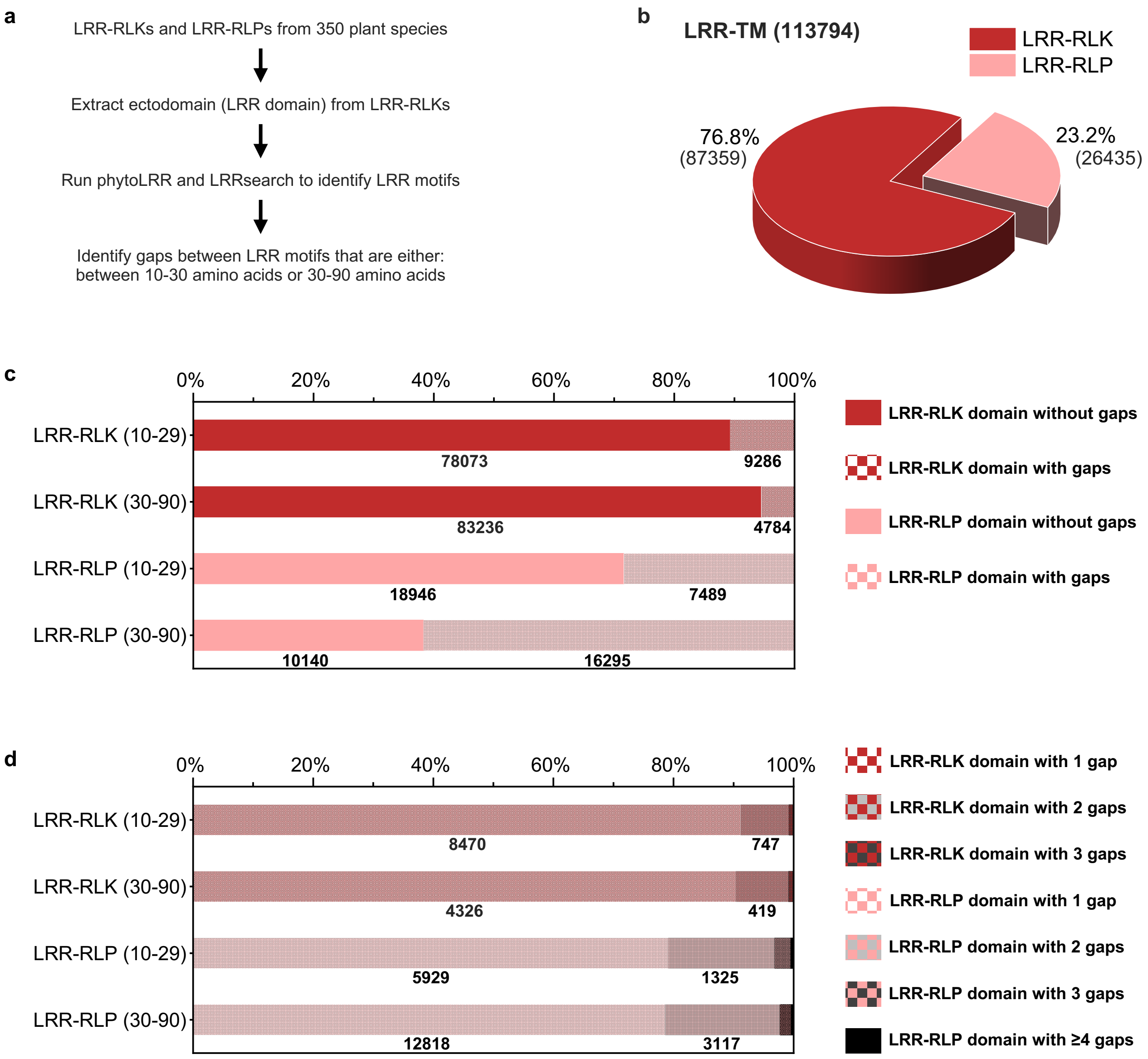

Supplementary figure 12. Distribution of gaps within LRR motifs in LRR-containing cell-surface receptors. (a) Method of gap identification from LRR-containing PRRs. For details, please refer to methods. **(b)** Distribution of LRR-RLKs and LRR-RLPs in LRR-containing cell surface receptors (LRR-TM) from 350 species. **(c)** Distribution of LRR-RLPs and LRR-RLKs with or without gaps of 10-29 amino acids (10-29) or 30-90 (30-90) amino acids. Labels are indicated on the right. **(d)** Distribution of LRR-RLPs and LRR-RLKs with one, two, three, or more than 3 gaps of 10-29 amino acids (10-29) or 30-90 (30-90) amino acids. Labels are indicated on the right.

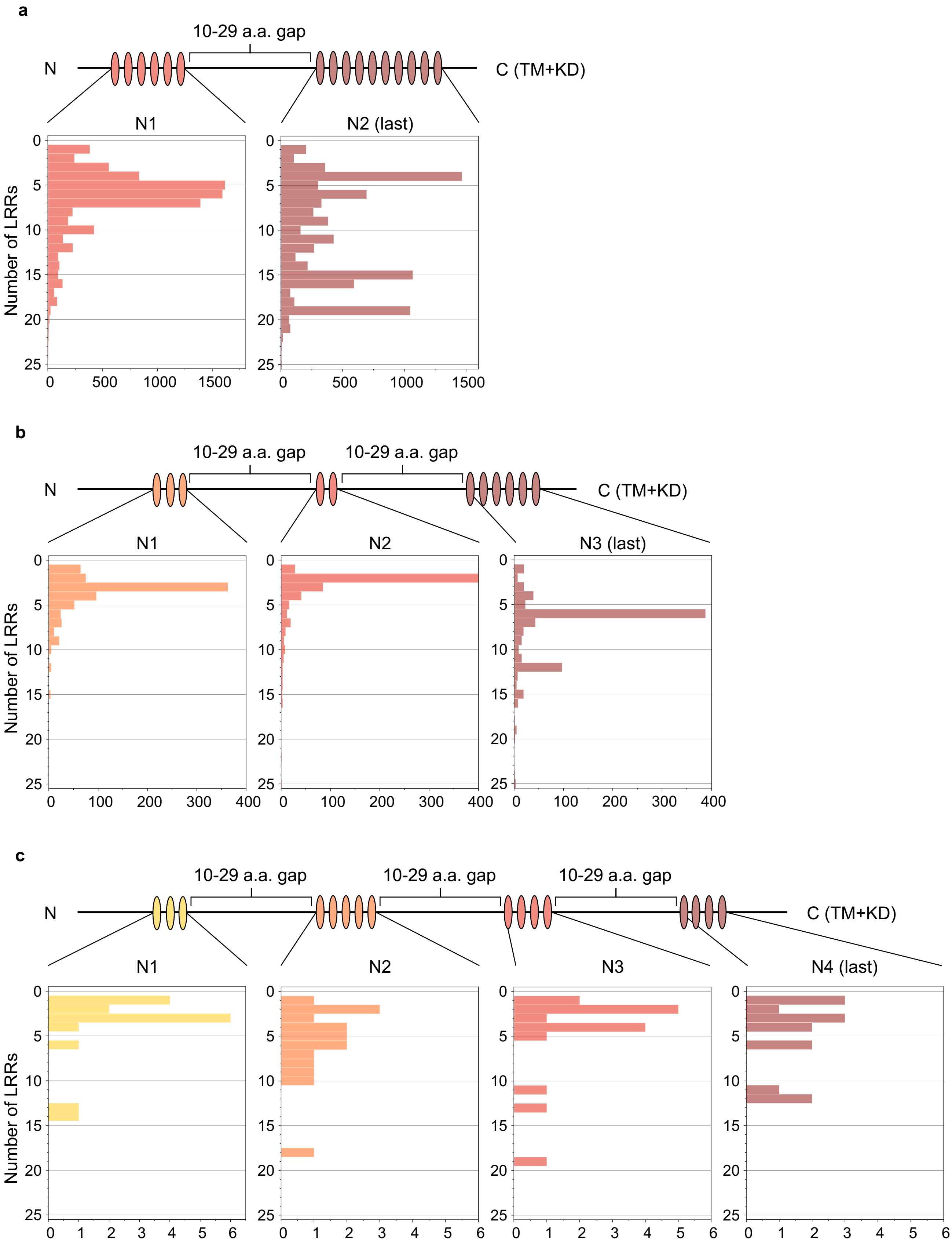

Supplementary figure 13a-c. Distribution of small gaps within LRR motifs in LRR-RLKs. Position of small gaps (NLs; 10-29 amino acids) in LRR-RLKs with **(a)** one, **(b)** two and **(c)** three small gaps. N1, N2, N3, and N4 represents the number of LRR motifs between the small gaps. X-axis presents number (n) of proteins.

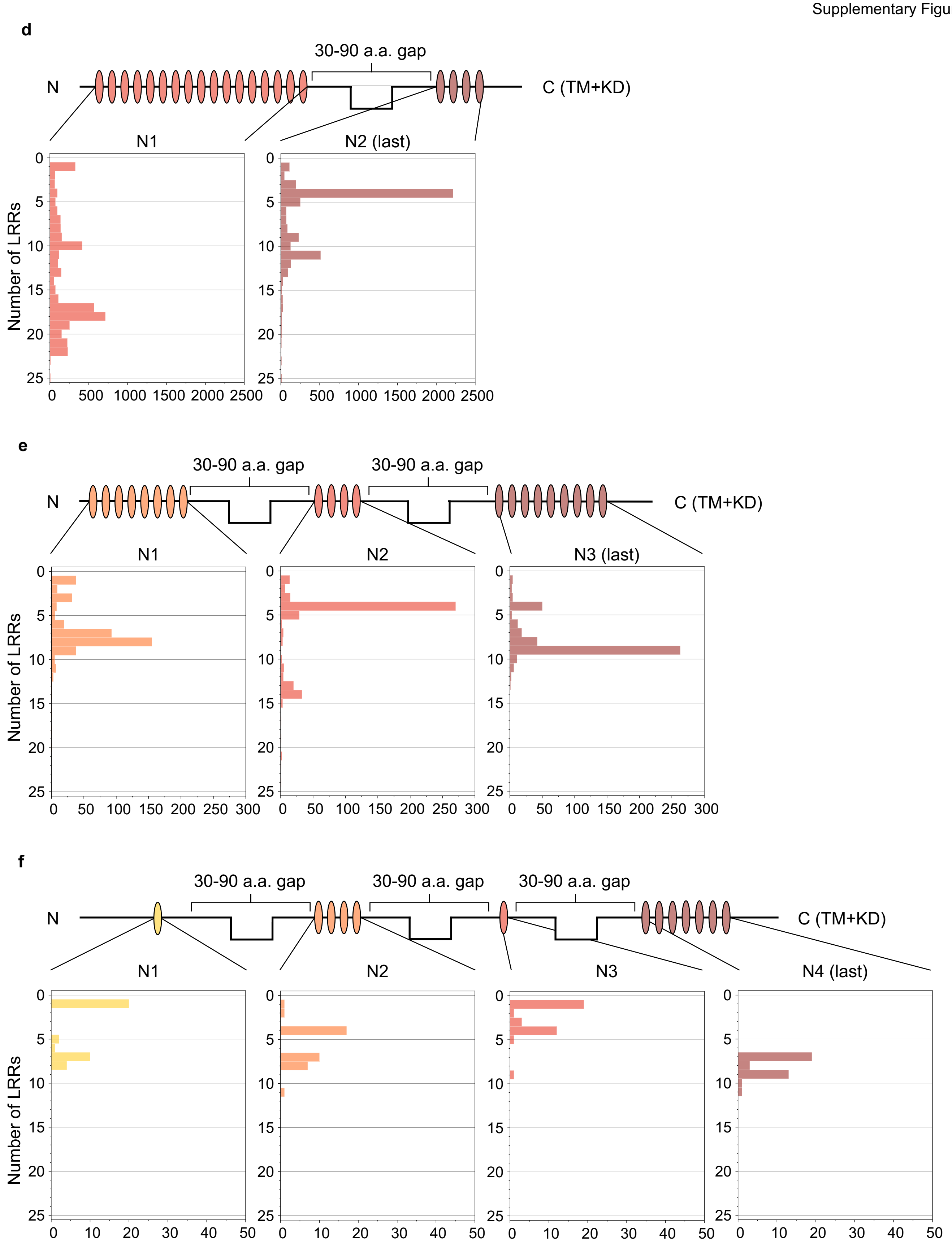

Supplementary figure 13d-f. Distribution of large gaps within LRR motifs in LRR-RLKs. Position of large gaps (IDs; 30-90 amino acids) in LRR-RLKs with **(d)** one, **(e)** two and **(f)** three large gaps. N1, N2, N3, and N4 represents the number of LRR motifs between the large gaps. X-axis presents number (n) of proteins.

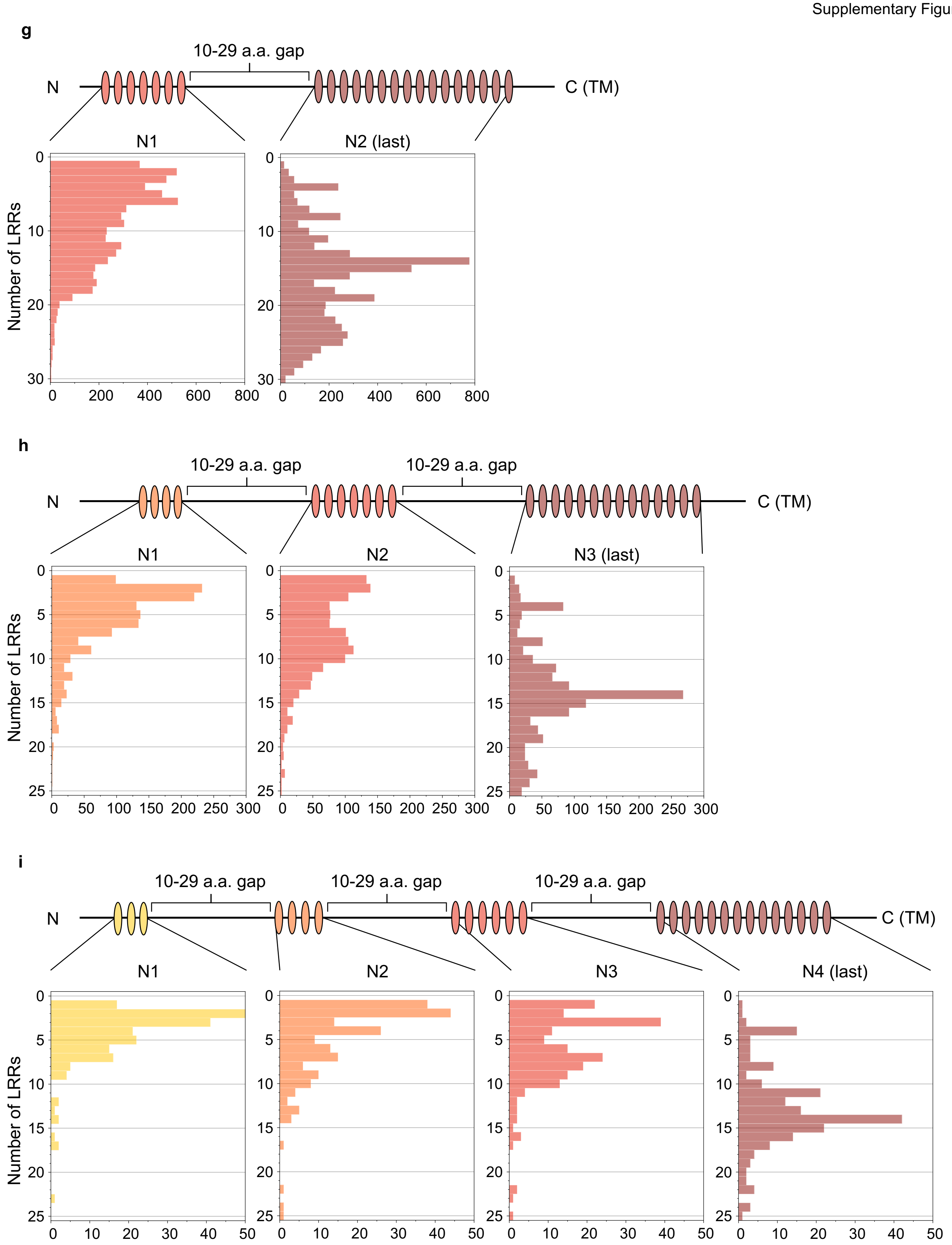

Supplementary figure 13g-i. Distribution of small gaps within LRR motifs in LRR-RLPs. Position of small gaps (NLs; 10-29 amino acids) in LRR-RLPs with **(g)** one, **(h)** two and **(i)** three small gaps. N1, N2, N3, and N4 represents the number of LRR motifs between the small gaps. X-axis presents number (n) of proteins.

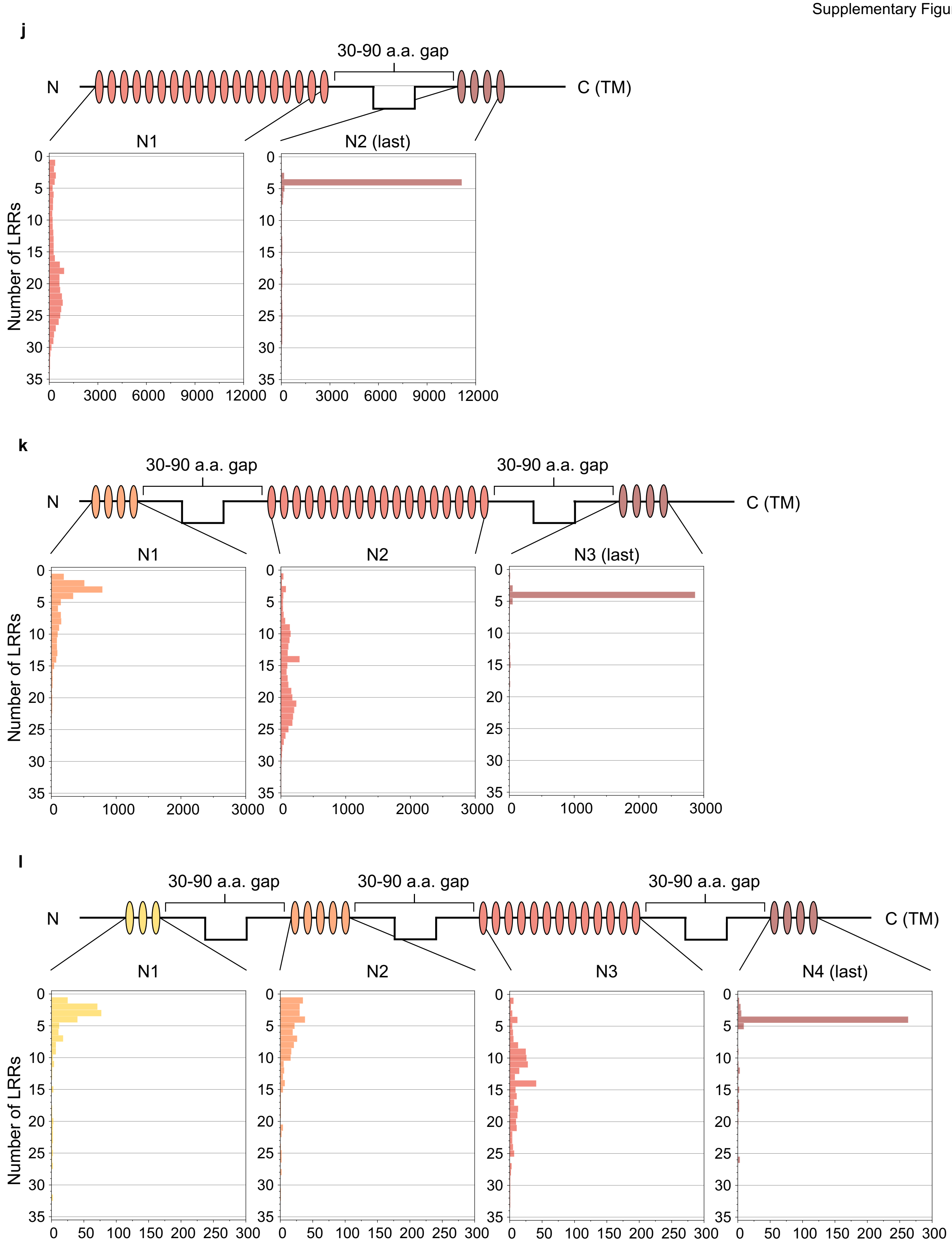

Supplementary figure 13j-l. Distribution of large gaps within LRR motifs in LRR-RLPs. (a-c) Position of large gaps (IDs; 30-90 amino acids) in LRR-RLPs with **(j)** one, **(k)** two and **(l)** three large gaps. N1, N2, N3, and N4 represents the number of LRR motifs between the large gaps. X-axis presents number (n) of proteins.

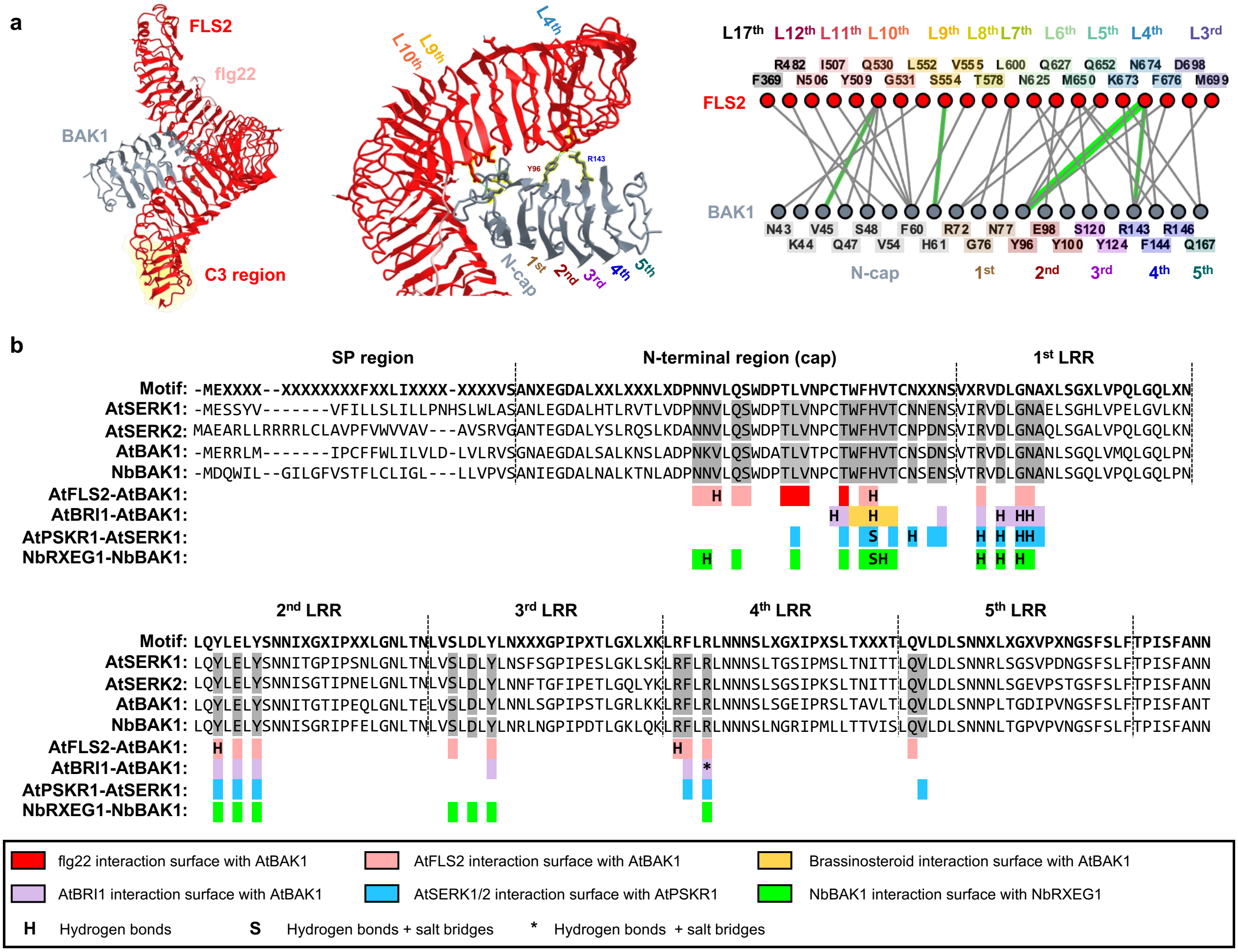

Extended figure 14. Interaction surfaces between SERKs, LRR-RLKs and LRR-RLP. Published structures of **(a)** AtFLS2-AtBAK1, The left panels show the full structure, and the middle panels show the interaction sites between LRR-RLKs or LRR-RLP and SERKs. Hydrogen bonds are indicated by green dotted lines, and salt bridges are shown as cyan dotted lines. The positions of LRR residues (counting from N to C for SERKs and counting from C to N for LRR-RLKs and LRR-RLP) are shown. The right panel represents the 2D interaction network between SERKs and its receptors. Contacts/interactions are shown in grey lines, hydrogen bonds are shown in green lines, and salt bridges are shown in cyan lines. Amino acids are labelled in colours according to their positions in the LRR motifs (counting from N to C for SERKs and counting from C to N for LRR-RLKs and LRR-RLP (L)). Structures were visualized in iCn3D. For **(a)**, the interaction sites are calculated by iCn3D with the following thresholds: hydrogen bonds: 4.2Å; salt bridges/ionic bonds: 6Å; contacts/interactions: 4Å. **(b)** Alignment of the ectodomains of SERKs from *A. thaliana* (At) and *N. benthamiana* (Nb). The amino acid residues between the interaction interfaces of AtFLS2-AtBAK1, AtBRI1-AtBAK1, AtPSKR1-AtSERK1, and NbRXEG1-NbBAK1 are highlighted in different colours as indicated in the boxes below. Hydrogen bonds and salt bridges/ionic bonds are also indicated.

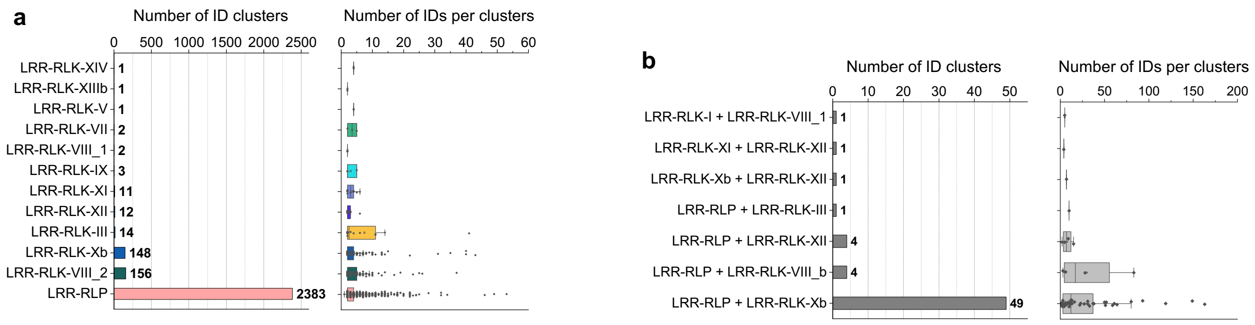

Supplementary figure 15. Motif enrichment analysis and clustering of IDs (a) Number of ID clusters with LRR-receptors from a single subgroup/family. The left graph shows the number of clusters containing LRR-receptors from each subgroup/family. The right graph shows the distribution of the sizes of each cluster (number of IDs in each cluster). **(b)** Number of ID clusters with LRR-receptors from two different subgroups/families. The graph on the left shows the number of clusters containing LRR-receptors with the combination of subgroups/families. The graph on the right shows the distribution of the sizes of each cluster (number of IDs in each cluster). Boxplot elements: center line, median; bounds of box, 25th and 75th percentiles; whiskers, 1.5 × IQR from 25th and 75th percentiles. For **a** and **b**, n (number of receptors in each classes) in the right graph is stated on the left graph.

a

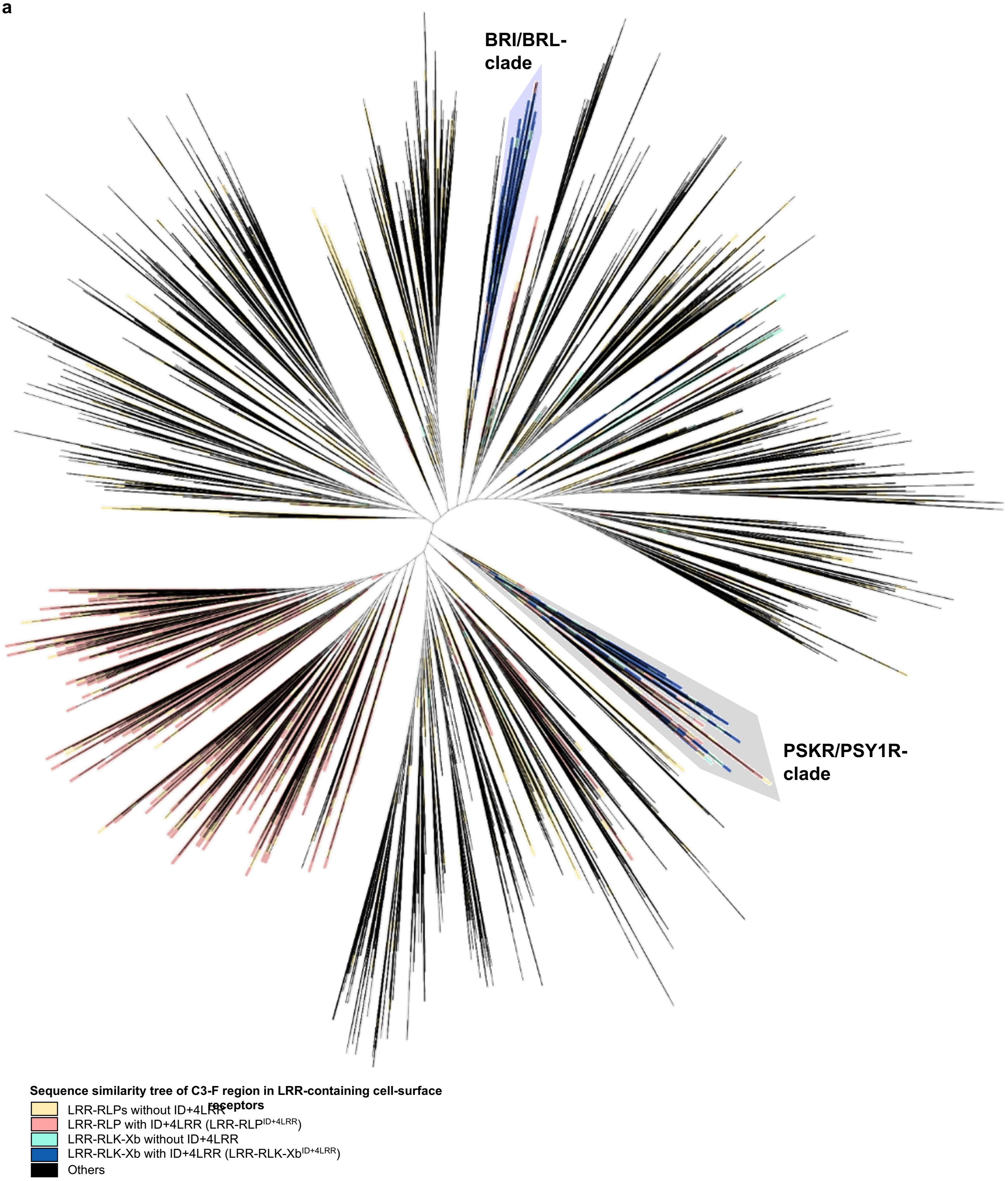

Supplementary figure 16a. Sequence similarity tree of the ectodomain region of all LRR-containing PRRs from 350 species. Clades and branches are labelled as indicated.

b

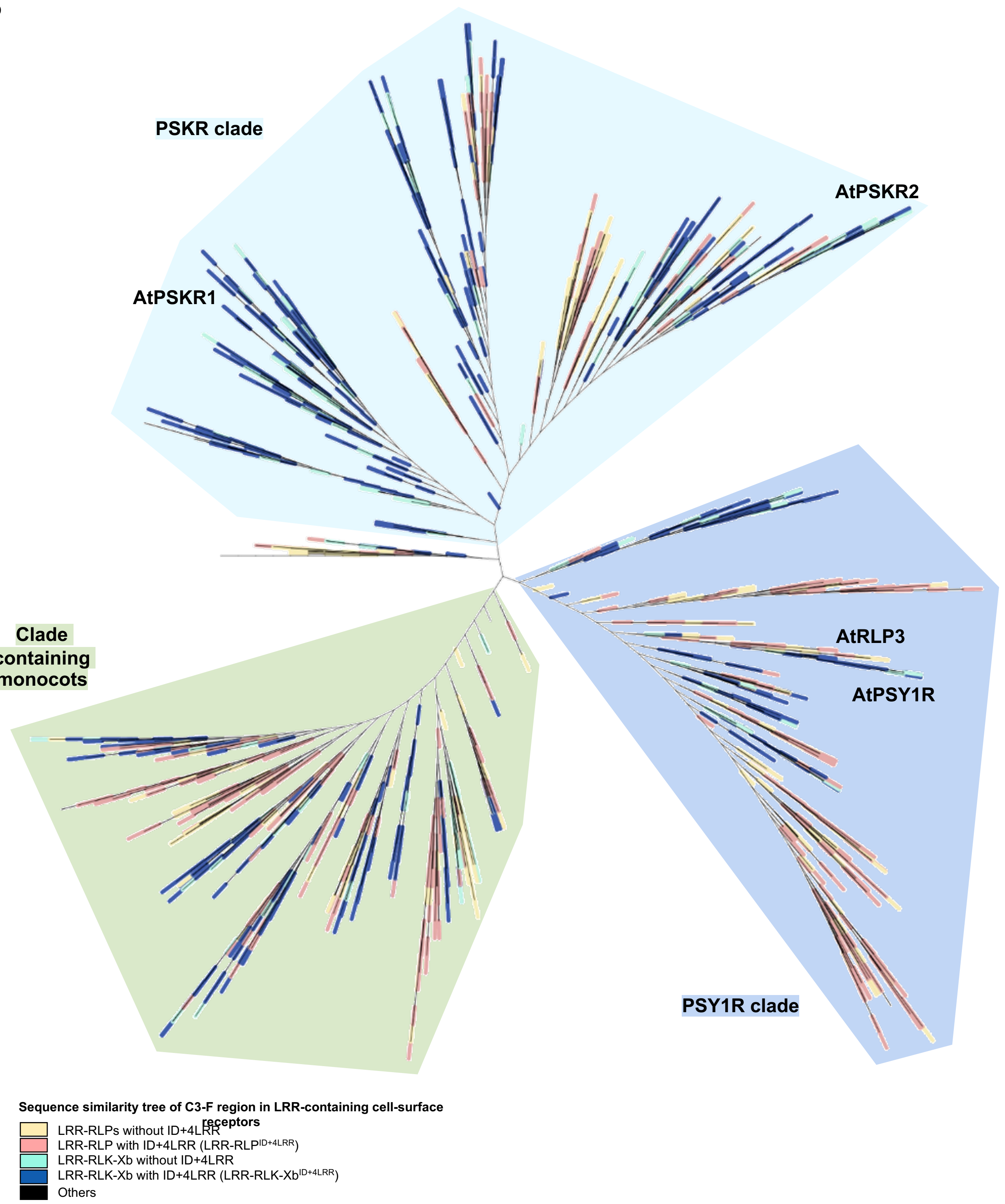

Supplementary figure 16b. Sequence similarity tree of the ectodomain region of all LRR-containing PRRs from 350 species within the PSKR/PSY1R-clade from (a). Clades and branches are labelled as indicated.

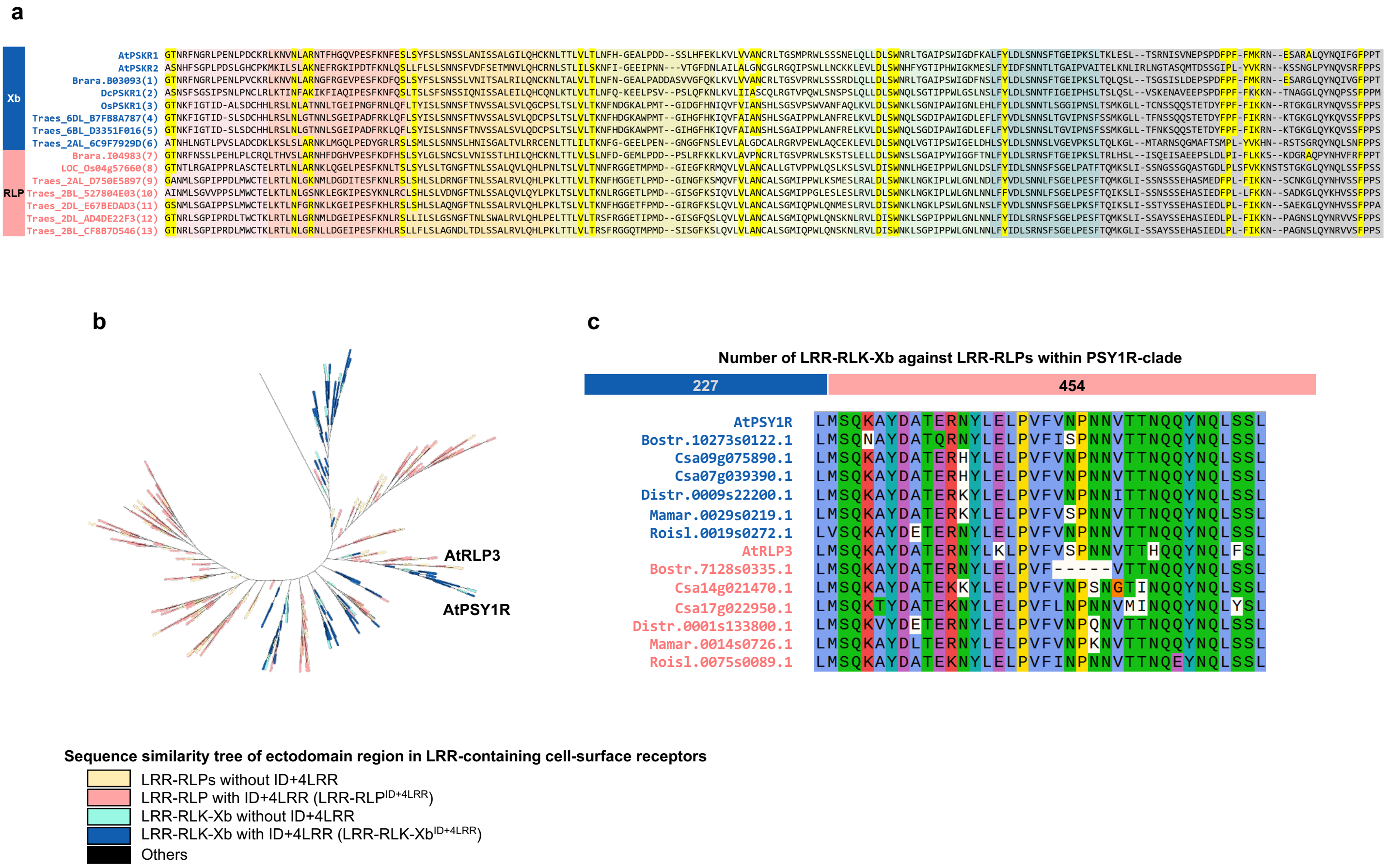

Supplementary figure 17. Ectodomains shared by LRR-RLK-Xbs and LRR-RLPs. (a) The full alignment of ectodomain from LRR-RLK-Xb (blue) and LRR-RLP (pink) members taken from the sequence similarity tree in Figure **5h**. The green highlights indicate the amino acids residues required from PSK binding in AtPSKR1. The green highlights are corresponding to the amino acids highlighted in the structure in Figure **5g**. The positions of the LRR motifs and ID in the alignment are indicated in colors shown in Figure **5g**. (the interaction network; right panel). **b** Sequence similarity tree of ectodomains of PSY1R members from 350 species extracted from the PSY1R clade from Supplementary Fig. 16b. **c** Right top bar represents the distribution of LRR-RLK-Xb (blue) against LRR-RLP (pink) within the sequence similarity tree. Bottom alignment represents the alignment of the IDs from LRR-RLK-Xb (blue) and LRR-RLP (pink) from members taken from the sequence similarity tree. Amino acid residues that are conserved are highlighted by their properties by Clustal X.

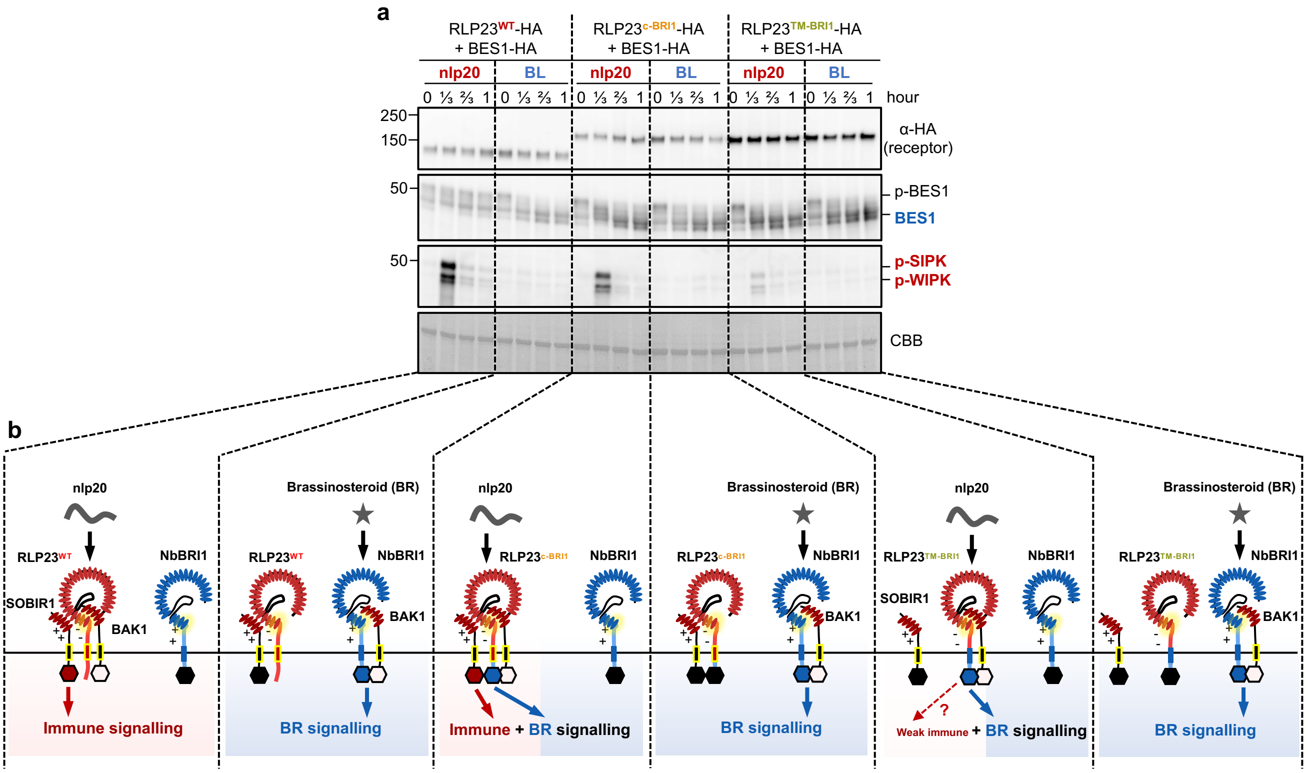

Supplementary figure 18. Activation of immune and development pathways by LRR-RLK-Xbs and LRR-RLPs. (a) Functionality testing of AtRLP23 chimeras with brassinosteroid (BL) as a positive control. Nb leaves expressing the indicated constructs were treated with 1µM nlp20 or BL, and samples were collected at indicated time points. Dephosphorylation of BESI1-HA was detected with HA antibody. Phosphorylation of NbSIPK and NbWIPK was detected with p-P42/44 antibody. The experiments were repeated at least twice with similar results. **(b)** Schematic model of the interaction between LRR-RLK-Xb (BRI1) and LRR-RLP (RLP23) with co-receptors to induce differential downstream signalling. Both receptor classes utilize the last 4 LRRs (highlighted in yellow) to interact with SERKs (BAK1). LRR-RLP evolved to interact with SOBIR1 with the GxxxG motifs in TM (highlighted in yellow outline). Colored hexagons on RLKs indicate activated kinases and black hexagon indicates an inactivated kinase.

References

1. Gómez-Gómez, L. & Boller, T. FLS2: an LRR receptor-like kinase involved in the perception of the bacterial elicitor flagellin in Arabidopsis. *Mol. Cell* **5,** 1003–1011 (2000).

2. Zipfel, C. *et al.* Perception of the bacterial PAMP EF-Tu by the receptor EFR restricts *Agrobacterium*-mediated transformation. *Cell* **125,** 749–760 (2006).

3. Song, W. Y. *et al.* A receptor kinase-like protein encoded by the rice disease resistance gene, Xa21. *Science* **270,** 1804–1806 (1995).

4. Ranf, S. *et al.* A lectin S-domain receptor kinase mediates lipopolysaccharide sensing in *Arabidopsis thaliana*. *Nat. Immunol.* **16,** 426–433 (2015).

5. Kato, H. *et al.* Recognition of pathogen-derived sphingolipids in Arabidopsis. *Science* **376,** 857–860 (2022).

6. Brutus, A., Sicilia, F., Macone, A., Cervone, F. & De Lorenzo, G. A domain swap approach reveals a role of the plant wall-associated kinase 1 (WAK1) as a receptor of oligogalacturonides. *Proc. Natl. Acad. Sci. USA* **107,** 9452–9457 (2010).

7. Decreux, A. & Messiaen, J. Wall-associated kinase WAK1 interacts with cell wall pectins in a calcium-induced conformation. *Plant Cell Physiol.* **46,** 268–278 (2005).

8. Yadeta, K. A. *et al.* A Cysteine-Rich Protein Kinase Associates with a Membrane Immune Complex and the Cysteine Residues Are Required for Cell Death. *Plant Physiol.* **173,** 771–787 (2017).

9. Acharya, B. R. *et al.* Overexpression of CRK13, an Arabidopsis cysteine-rich receptor-like kinase, results in enhanced resistance to Pseudomonas syringae. *The Plant Journal* **50,** 488–499 (2007).

10. Bourdais, G. *et al.* Large-Scale Phenomics Identifies Primary and Fine-Tuning Roles for CRKs in Responses Related to Oxidative Stress. *PLoS Genet.* **11,** e1005373 (2015).

11. Choi, J. *et al.* Identification of a plant receptor for extracellular ATP. *Science* **343,** 290–294 (2014).

12. Gouhier-Darimont, C., Stahl, E., Glauser, G. & Reymond, P. The Arabidopsis Lectin Receptor Kinase LecRK-I.8 Is Involved in Insect Egg Perception. *Front. Plant Sci.* **10,** 623 (2019).

13. Miya, A. *et al.* CERK1, a LysM receptor kinase, is essential for chitin elicitor signalling in Arabidopsis. *Proc. Natl. Acad. Sci. USA* **104,** 19613–19618 (2007).

14. Stegmann, M. *et al.* The receptor kinase FER is a RALF-regulated scaffold controlling plant immune signalling. *Science* **355,** 287–289 (2017).

15. Duan, Q., Kita, D., Li, C., Cheung, A. Y. & Wu, H.-M. FERONIA receptor-like kinase regulates RHO GTPase signalling of root hair development. *Proc. Natl. Acad. Sci. USA* **107,** 17821–17826 (2010).

16. Haruta, M., Sabat, G., Stecker, K., Minkoff, B. B. & Sussman, M. R. A peptide hormone and its receptor protein kinase regulate plant cell expansion. *Science* **343,** 408–411 (2014).

17. Ge, Z. *et al.* Arabidopsis pollen tube integrity and sperm release are regulated by RALF-mediated signalling. *Science* **358,** 1596–1600 (2017).

18. Huck, N., Moore, J. M., Federer, M. & Grossniklaus, U. The Arabidopsis mutant feronia disrupts the female gametophytic control of pollen tube reception. *Development* **130,** 2149–2159 (2003).

19. Tseng, Y.-H. *et al.* CORK1, A LRR-Malectin Receptor Kinase, Is Required for Cellooligomer-Induced Responses in Arabidopsis thaliana. *Cells* **11,** (2022).

20. Trotochaud, A. E., Jeong, S. & Clark, S. E. CLAVATA3, a multimeric ligand for the CLAVATA1 receptor-kinase. *Science* **289,** 613–617 (2000).

21. Jinn, T. L., Stone, J. M. & Walker, J. C. HAESA, an Arabidopsis leucine-rich repeat receptor kinase, controls floral organ abscission. *Genes Dev.* **14,** 108–117 (2000).

22. Takasaki, T. *et al.* The S receptor kinase determines self-incompatibility in Brassica stigma. *Nature* **403,** 913–916 (2000).

23. Wang, N. *et al.* The rice wall-associated receptor-like kinase gene *OsDEES1* plays a role in female gametophyte development. *Plant Physiol.* **160,** 696–707 (2012).

24. Wan, J. *et al.* A lectin receptor-like kinase is required for pollen development in Arabidopsis. *Plant Mol. Biol.* **67,** 469–482 (2008).

25. Peng, X. *et al.* Lectin receptor kinase OsLecRK-S.7 is required for pollen development and male fertility. *J. Integr. Plant Biol.* **62,** 1227–1245 (2020).

26. Li, J. & Chory, J. A putative leucine-rich repeat receptor kinase involved in brassinosteroid signal transduction. *Cell* **90,** 929–938 (1997).

27. Shinohara, H., Mori, A., Yasue, N., Sumida, K. & Matsubayashi, Y. Identification of three LRR-RKs involved in perception of root meristem growth factor in Arabidopsis. *Proc. Natl. Acad. Sci. USA* **113,** 3897–3902 (2016).

28. Matsuzaki, Y., Ogawa-Ohnishi, M., Mori, A. & Matsubayashi, Y. Secreted peptide signals required for maintenance of root stem cell niche in Arabidopsis. *Science* **329,** 1065–1067 (2010).

29. Xu, T. *et al.* Cell surface ABP1-TMK auxin-sensing complex activates ROP GTPase signalling. *Science* **343,** 1025–1028 (2014).

30. Chen, L.-J. *et al.* An S-domain receptor-like kinase OsSIK2 confers abiotic stress tolerance and delays dark-induced leaf senescence in rice. *Plant Physiol.* **163,** 1752–1765 (2013).

31. Lally, D., Ingmire, P., Tong, H.-Y. & He, Z.-H. Antisense expression of a cell wall–associated protein kinase, WAK4, inhibits cell elongation and alters morphology. *Plant Cell* **13,** 1317–1332 (2001).

32. Tanaka, H. *et al.* Abiotic stress-inducible receptor-like kinases negatively control ABA signalling in Arabidopsis. *The Plant Journal* **70,** 599–613 (2012).

33. Lu, K. *et al.* Overexpression of an Arabidopsis cysteine-rich receptor-like protein kinase, CRK5, enhances abscisic acid sensitivity and confers drought tolerance. *J. Exp. Bot.* **67,** 5009–5027 (2016).

34. Xin, Z., Wang, A., Yang, G., Gao, P. & Zheng, Z.-L. The Arabidopsis A4 subfamily of lectin receptor kinases negatively regulates abscisic acid response in seed germination. *Plant Physiol.* **149,** 434–444 (2009).

35. Joosten, M. H., Vogelsang, R., Cozijnsen, T. J., Verberne, M. C. & De Wit, P. J. The biotrophic fungus Cladosporium fulvum circumvents Cf-4-mediated resistance by producing unstable AVR4 elicitors. *Plant Cell* **9,** 367–379 (1997).

36. Albert, I. *et al.* An RLP23-SOBIR1-BAK1 complex mediates NLP-triggered immunity. *Nat. Plants* **1,** 15140 (2015).

37. Wang, Y. *et al.* Leucine-rich repeat receptor-like gene screen reveals that Nicotiana RXEG1 regulates glycoside hydrolase 12 MAMP detection. *Nat. Commun.* **9,** 594 (2018).

38. Willmann, R. *et al.* Arabidopsis lysin-motif proteins LYM1 LYM3 CERK1 mediate bacterial peptidoglycan sensing and immunity to bacterial infection. *Proc. Natl. Acad. Sci. USA* **108,** 19824–19829 (2011).

39. Liu, B. *et al.* Lysin motif-containing proteins LYP4 and LYP6 play dual roles in peptidoglycan and chitin perception in rice innate immunity. *Plant Cell* **24,** 3406–3419 (2012).

40. Jeong, S., Trotochaud, A. E. & Clark, S. E. The Arabidopsis CLAVATA2 gene encodes a receptor-like protein required for the stability of the CLAVATA1 receptor-like kinase. *Plant Cell* **11,** 1925–1934 (1999).

41. Nadeau, J. A. & Sack, F. D. Control of stomatal distribution on the Arabidopsis leaf surface. *Science* **296,** 1697–1700 (2002).

42. Roux, M. *et al.* The *Arabidopsis* leucine-rich repeat receptor-like kinases BAK1/SERK3 and BKK1/SERK4 are required for innate immunity to hemibiotrophic and biotrophic pathogens. *Plant Cell* **23,** 2440–2455 (2011).

43. Heese, A. *et al.* The receptor-like kinase SERK3/BAK1 is a central regulator of innate immunity in plants. *Proc. Natl. Acad. Sci. USA* **104,** 12217–12222 (2007).

44. Nam, K. H. & Li, J. BRI1/BAK1, a receptor kinase pair mediating brassinosteroid signalling. *Cell* **110,** 203–212 (2002).

45. Chinchilla, D. *et al.* A flagellin-induced complex of the receptor FLS2 and BAK1 initiates plant defence. *Nature* **448,** 497–500 (2007).

46. Liebrand, T. W. H. *et al.* Receptor-like kinase SOBIR1/EVR interacts with receptor-like proteins in plant immunity against fungal infection. *Proc. Natl. Acad. Sci. USA* **110,** 10010–10015 (2013).

47. Lu, D. *et al.* A receptor-like cytoplasmic kinase, BIK1, associates with a flagellin receptor complex to initiate plant innate immunity. *Proc. Natl. Acad. Sci. USA* **107,** 496–501 (2010).

48. Zhang, J. *et al.* Receptor-like cytoplasmic kinases integrate signalling from multiple plant immune receptors and are targeted by a Pseudomonas syringae effector. *Cell Host Microbe* **7,** 290–301 (2010).

49. Bi, G. *et al.* Receptor-Like Cytoplasmic Kinases Directly Link Diverse Pattern Recognition Receptors to the Activation of Mitogen-Activated Protein Kinase Cascades in Arabidopsis. *Plant Cell* **30,** 1543–1561 (2018).

50. Pruitt, R. N. *et al.* The EDS1-PAD4-ADR1 node mediates Arabidopsis pattern-triggered immunity. *Nature* **598,** 495–499 (2021).

51. Monaghan, J. *et al.* The calcium-dependent protein kinase CPK28 buffers plant immunity and regulates BIK1 turnover. *Cell Host Microbe* **16,** 605–615 (2014).

52. Gao, X. *et al.* Bifurcation of Arabidopsis NLR immune signalling via Ca^{2+}-dependent protein kinases. *PLoS Pathog.* **9,** e1003127 (2013).

53. Boudsocq, M. *et al.* Differential innate immune signalling via Ca(2+) sensor protein kinases. *Nature* **464,** 418–422 (2010).

54. Asai, T. *et al.* MAP kinase signalling cascade in Arabidopsis innate immunity. *Nature* **415,** 977–983 (2002).

55. Meena, M. K. *et al.* The ca2+ channel CNGC19 regulates arabidopsis defense against spodoptera herbivory. *Plant Cell* **31,** 1539–1562 (2019).

56. Yu, X. *et al.* The Receptor Kinases BAK1/SERK4 Regulate Ca2+ Channel-Mediated Cellular Homeostasis for Cell Death Containment. *Curr. Biol.* **29,** 3778–3790.e8 (2019).

57. Tian, W. *et al.* A calmodulin-gated calcium channel links pathogen patterns to plant immunity. *Nature* **572,** 131–135 (2019).

58. Thor, K. *et al.* The calcium-permeable channel OSCA1.3 regulates plant stomatal immunity. *Nature* **585,** 569–573 (2020).

59. Torres, M. A. *et al.* Six Arabidopsis thaliana homologues of the human respiratory burst oxidase (gp91phox). *The Plant Journal* **14,** 365–370 (1998).

60. Torres, M. A., Dangl, J. L. & Jones, J. D. G. Arabidopsis gp91phox homologues AtrbohD and AtrbohF are required for accumulation of reactive oxygen intermediates in the plant defense response. *Proc. Natl. Acad. Sci. USA* **99,** 517–522 (2002).

61. Parker, J. E. *et al.* Characterization of eds1, a mutation in Arabidopsis suppressing resistance to Peronospora parasitica specified by several different RPP genes. *Plant Cell* **8,** 2033–2046 (1996).

62. Zhou, N., Tootle, T. L., Tsui, F., Klessig, D. F. & Glazebrook, J. PAD4 functions upstream from salicylic acid to control defense responses in Arabidopsis. *Plant Cell* **10,** 1021–1030 (1998).

63. Jirage, D. *et al.* Arabidopsis thaliana PAD4 encodes a lipase-like gene that is important for salicylic acid signalling. *Proc. Natl. Acad. Sci. USA* **96,** 13583–13588 (1999).

64. Feys, B. J., Moisan, L. J., Newman, M. A. & Parker, J. E. Direct interaction between the Arabidopsis disease resistance signalling proteins, EDS1 and PAD4. *EMBO J.* **20,** 5400–5411 (2001).

65. Feys, B. J. *et al.* Arabidopsis SENESCENCE-ASSOCIATED GENE101 stabilizes and signals within an ENHANCED DISEASE SUSCEPTIBILITY1 complex in plant innate immunity. *Plant Cell* **17,** 2601–2613 (2005).

66. Bonardi, V. *et al.* Expanded functions for a family of plant intracellular immune receptors beyond specific recognition of pathogen effectors. *Proc. Natl. Acad. Sci. USA* **108,** 16463–16468 (2011).

67. Peart, J. R., Mestre, P., Lu, R., Malcuit, I. & Baulcombe, D. C. NRG1, a CC-NB-LRR protein, together with N, a TIR-NB-LRR protein, mediates resistance against tobacco mosaic virus. *Curr. Biol.* **15,** 968–973 (2005).

68. Zhang, Y. *et al.* Control of salicylic acid synthesis and systemic acquired resistance by two members of a plant-specific family of transcription factors. *Proc. Natl. Acad. Sci. USA* **107,** 18220–18225 (2010).

69. Li, C.-L. *et al.* THI1, a Thiamine Thiazole Synthase, Interacts with Ca2+-Dependent Protein Kinase CPK33 and Modulates the S-Type Anion Channels and Stomatal Closure in Arabidopsis. *Plant Physiol.* **170,** 1090–1104 (2016).

70. Zhao, L.-N. *et al.* Ca2+-dependent protein kinase11 and 24 modulate the activity of the inward rectifying K+ channels in Arabidopsis pollen tubes. *Plant Cell* **25,** 649–661 (2013).

71. Myers, C. *et al.* Calcium-dependent protein kinases regulate polarized tip growth in pollen tubes. *The Plant Journal* **59,** 528–539 (2009).

72. Tunc-Ozdemir, M. *et al.* Cyclic nucleotide gated channels 7 and 8 are essential for male reproductive fertility. *PLoS One* **8,** e55277 (2013).

73. Tunc-Ozdemir, M. *et al.* A cyclic nucleotide-gated channel (CNGC16) in pollen is critical for stress tolerance in pollen reproductive development. *Plant Physiol.* **161,** 1010–1020 (2013).

74. Gao, Q.-F. *et al.* Cyclic nucleotide-gated channel 18 is an essential Ca2+ channel in pollen tube tips for pollen tube guidance to ovules in Arabidopsis. *Proc. Natl. Acad. Sci. USA* **113,** 3096–3101 (2016).

75. Kaya, H. *et al.* Ca2+-activated reactive oxygen species production by Arabidopsis RbohH and RbohJ is essential for proper pollen tube tip growth. *Plant Cell* **26,** 1069–1080 (2014).

76. Matschi, S. *et al.* Function of calcium-dependent protein kinase CPK28 of Arabidopsis thaliana in plant stem elongation and vascular development. *The Plant Journal* **73,** 883–896 (2013).

77. Matschi, S., Hake, K., Herde, M., Hause, B. & Romeis, T. The calcium-dependent protein kinase CPK28 regulates development by inducing growth phase-specific, spatially restricted alterations in jasmonic acid levels independent of defense responses in Arabidopsis. *Plant Cell* **27,** 591–606 (2015).

78. Liu, K.-H. *et al.* Discovery of nitrate-CPK-NLP signalling in central nutrient-growth networks. *Nature* **545,** 311–316 (2017).

79. Tan, Y.-Q. *et al.* Three CNGC Family Members, CNGC5, CNGC6, and CNGC9, Are Required for Constitutive Growth of Arabidopsis Root Hairs as Ca2+-Permeable Channels. *Plant Commun.* **1,** 100001 (2020).

80. Zhang, X. *et al.* CBL1-CIPK26 mediated phosphorylation enhances activity of the NADPH oxidase RBOHC, but is dispensable for root hair growth. *FEBS Lett.* **592,** 2582–2593 (2018).

81. Jehle, A. K. *et al.* The receptor-like protein ReMAX of *Arabidopsis* detects the microbe-associated molecular pattern eMax from *Xanthomonas*. *Plant Cell* **25,** 2330–2340 (2013).

82. Zhang, W. *et al.* Arabidopsis receptor-like protein30 and receptor-like kinase suppressor of BIR1-1/EVERSHED mediate innate immunity to necrotrophic fungi. *Plant Cell* **25,** 4227–4241 (2013).

83. Fan, L. *et al.* Genotyping-by-sequencing-based identification of Arabidopsis pattern recognition receptor RLP32 recognizing proteobacterial translation initiation factor IF1. *Nat. Commun.* **13,** 1294 (2022).

84. Zhang, L. *et al.* Fungal endopolygalacturonases are recognized as microbe-associated molecular patterns by the *Arabidopsis* receptor-like protein RESPONSIVENESS TO BOTRYTIS POLYGALACTURONASES1. *Plant Physiol.* **164,** 352–364 (2014).

85. Saur, I. M. L. *et al.* NbCSPR underlies age-dependent immune responses to bacterial cold shock protein in *Nicotiana benthamiana*. *Proc. Natl. Acad. Sci. USA* **113,** 3389–3394 (2016).

86. Dixon, M. S. *et al.* The tomato Cf-2 disease resistance locus comprises two functional genes encoding leucine-rich repeat proteins. *Cell* **84,** 451–459 (1996).

87. Jones, D. A., Thomas, C. M., Hammond-Kosack, K. E., Balint-Kurti, P. J. & Jones, J. D. G. Isolation of the tomato *Cf-9* gene for resistance to *Cladosporium fulvum* by transposon tagging. *Science* **266,** 789–793 (1994).

88. Dixon, M. S., Hatzixanthis, K., Jones, D. A., Harrison, K. & Jones, J. D. The tomato Cf-5 disease resistance gene and six homologs show pronounced allelic variation in leucine-rich repeat copy number. *Plant Cell* **10,** 1915–1925 (1998).

89. Krüger, J. *et al.* A tomato cysteine protease required for Cf-2-dependent disease resistance and suppression of autonecrosis. *Science* **296,** 744–747 (2002).

90. Westerink, N., Brandwagt, B. F., de Wit, P. J. G. M. & Joosten, M. H. A. J. Cladosporium fulvum circumvents the second functional resistance gene homologue at the Cf-4 locus (Hcr9-4E) by secretion of a stable avr4E isoform. *Mol. Microbiol.* **54,** 533–545 (2004).

91. Ron, M. & Avni, A. The receptor for the fungal elicitor ethylene-inducing xylanase is a member of a resistance-like gene family in tomato. *Plant Cell* **16,** 1604–1615 (2004).

92. de Jonge, R. *et al.* Tomato immune receptor Ve1 recognizes effector of multiple fungal pathogens uncovered by genome and RNA sequencing. *Proc. Natl. Acad. Sci. USA* **109,** 5110–5115 (2012).

93. Fradin, E. F. *et al.* Genetic dissection of Verticillium wilt resistance mediated by tomato Ve1. *Plant Physiol.* **150,** 320–332 (2009).

94. Houterman, P. M., Cornelissen, B. J. C. & Rep, M. Suppression of plant resistance gene-based immunity by a fungal effector. *PLoS Pathog.* **4,** e1000061 (2008).

95. Du, J. *et al.* Elicitin recognition confers enhanced resistance to Phytophthora infestans in potato. *Nat. Plants* **1,** 15034 (2015).

96. Domazakis, E. *et al.* ELR is a true pattern recognition receptor that associates with elicitins from diverse Phytophthora species. *BioRxiv* (2020). doi:10.1101/2020.09.21.305813

97. Larkan, N. J. *et al.* The Brassica napus blackleg resistance gene LepR3 encodes a receptor-like protein triggered by the Leptosphaeria maculans effector AVRLM1. *New Phytol.* **197,** 595–605 (2013).

98. Hegenauer, V. *et al.* The tomato receptor CuRe1 senses a cell wall protein to identify Cuscuta as a pathogen. *Nat. Commun.* **11,** 5299 (2020).

99. Steinbrenner, A. D. *et al.* A receptor-like protein mediates plant immune responses to herbivore-associated molecular patterns. *Proc. Natl. Acad. Sci. USA* **117,** 31510–31518 (2020).

100. Chen, Z. *et al.* Convergent evolution of immune receptors underpins distinct elicitin recognition in closely related Solanaceous plants. *Plant Cell* (2023). doi:10.1093/plcell/koad002

101. Rao, S. *et al.* Roles of Receptor-Like Cytoplasmic Kinase VII Members in Pattern-Triggered Immune signalling. *Plant Physiol.* **177,** 1679–1690 (2018).

102. Hu, Z. *et al.* Genome-Wide Identification and Expression Analysis of Calcium-dependent Protein Kinase in Tomato. *Front. Plant Sci.* **7,** 469 (2016).

103. Liu, W. *et al.* Genome-wide survey and expression analysis of calcium-dependent protein kinase in Gossypium raimondii. *PLoS One* **9,** e98189 (2014).

104. Hamel, L.-P. *et al.* Ancient signals: comparative genomics of plant MAPK and MAPKK gene families. *Trends Plant Sci.* **11,** 192–198 (2006).

105. González-Coronel, J. M., Rodríguez-Alonso, G. & Guevara-García, Á. A. A phylogenetic study of the members of the MAPK and MEK families across Viridiplantae. *PLoS One* **16,** e0250584 (2021).

106. Mäser, P. *et al.* Phylogenetic relationships within cation transporter families of Arabidopsis. *Plant Physiol.* **126,** 1646–1667 (2001).

107. Chakraborty, S. *et al.* Genome-wide characterization and comparative analysis of the OSCA gene family and identification of its potential stress-responsive members in legumes. *Sci. Rep.* **13,** 5914 (2023).

108. Cheng, C. *et al.* Genome-wide analysis of respiratory burst oxidase homologs in grape (Vitis vinifera L.). *Int. J. Mol. Sci.* **14,** 24169–24186 (2013).

109. Li, X. *et al.* Tomato SlRbohB, a member of the NADPH oxidase family, is required for disease resistance against Botrytis cinerea and tolerance to drought stress. *Front. Plant Sci.* **6,** 463 (2015).

110. Lapin, D. *et al.* A Coevolved EDS1-SAG101-NRG1 Module Mediates Cell Death signalling by TIR-Domain Immune Receptors. *Plant Cell* **31,** 2430–2455 (2019).

111. Van Ghelder, C. *et al.* The large repertoire of conifer NLR resistance genes includes drought responsive and highly diversified RNLs. *Sci. Rep.* **9,** 11614 (2019).

112. Lin, G. *et al.* A receptor-like protein acts as a specificity switch for the regulation of stomatal development. *Genes Dev.* **31,** 927–938 (2017).

113. Sun, Y. *et al.* Plant receptor-like protein activation by a microbial glycoside hydrolase. *Nature* **610,** 335–342 (2022).

114. Jumper, J. *et al.* Highly accurate protein structure prediction with AlphaFold. *Nature* **596,** 583–589 (2021).

115. Wang, J. *et al.* iCn3D, a web-based 3D viewer for sharing 1D/2D/3D representations of biomolecular structures. *Bioinformatics* **36,** 131–135 (2020).

116.. Yotsui, I. *et al.* Genetic and phosphoproteomic basis of LysM-mediated immune signalling in *Marchantia polymorpha* highlights conserved elements and new aspect of pattern-triggered immunity in land plants. *BioRxiv* (2022). doi:10.1101/2022.12.28.521631

117. Chu, J. *et al.* Conservation of the PBL-RBOH immune module in land plants. *Curr. Biol.* **33,** 1130–1137.e5 (2023).

118. Weinberger, F. Pathogen-induced defense and innate immunity in macroalgae. *Biol Bull* **213,** 290–302 (2007).
