# Supplementary material for: Evolutionary trajectory of pattern recognition receptors in plants
Source: Nat Commun. 2024 Feb 1;15:308. doi: 10.1038/s41467-023-44408-3 (PMC10834447; doi:10.1038/s41467-023-44408-3)
Supplement: Supplementary file 3 — Description of Additional Supplementary Files [file 41467_2023_44408_MOESM3_ESM.pdf]

Description of Additional Supplementary Files:

Supplementary Data 1: Ecto- and Endo-domain analysis of cell-surface receptors.

Supplementary Data 2: LRR-RLP ID groups with subclass classifications.

Supplementary Data 3: Protein counts per species.
