# Supplementary material for: Evolutionary trajectory of pattern recognition receptors in plants
Source: Nat Commun. 2024 Feb 1;15:308. doi: 10.1038/s41467-023-44408-3 (PMC10834447; doi:10.1038/s41467-023-44408-3)
Supplement: Supplementary file 4 — Supplementary Data 1 [file 41467_2023_44408_MOESM4_ESM.pdf]

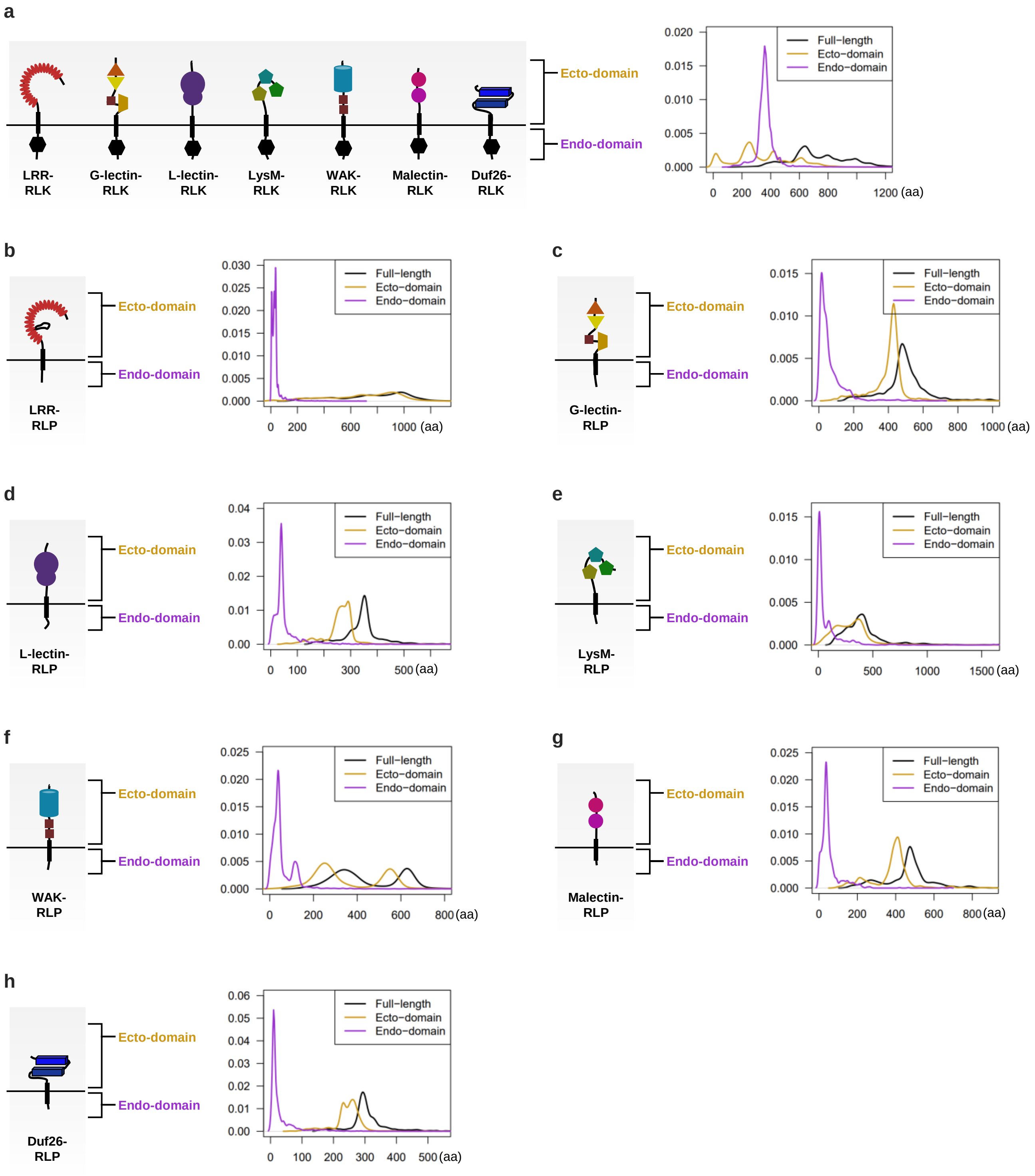

Supplementary Data 1a. Ecto- and endo-domain analysis of RLKs and RLPs. The length (in amino acid; AA) of full-protein, ecto-domains and endo-domains of **a** RLKs, **b** LRR-RLPs, **c** G-lectin-RLPs, **d** L-lectin-RLPs, **e** LysM-RLPs, **f** WAK-RLPs, **g** Malectin-RLPs, and **h** Duf26-RLPs are shown in the distribution plot on the right. Y-axis represents length (in AA; aa), X-axis represents the relative proportion of input proteins for the analysis.

a

Searched for all available PFAM HMMs in the endo-domains (e-value threshold of 1e-10) → Kept the hit with the best score for each endo-domain → Count number of hits per PFAM domains → Kept domains that were found in more than 1 or 1 out of 10,000 input sequences, filter out endo-domains that match any ecto-domains. → Final PFAM list

b

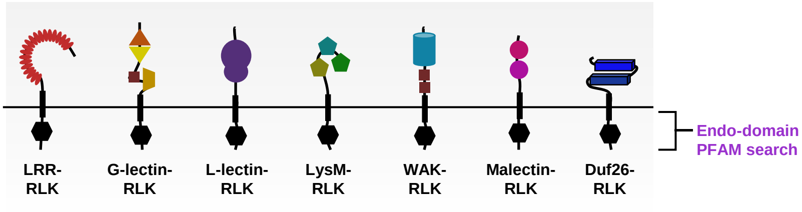

PFAM	Domain	Count	Percentage (%)
PF00069.26	Protein kinase domain	104,915	59.06
PF07714.18	Protein tyrosine and serine/threonine kinase	71704	40.36
PF06479.13	Ribonuclease 2-5A	192	0.11

c

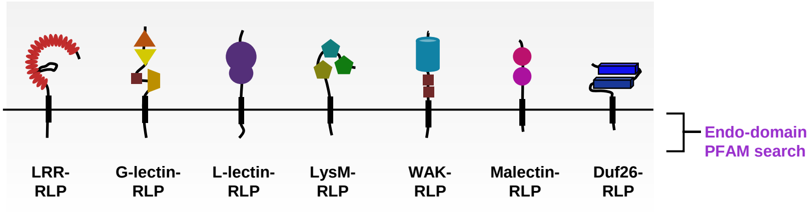

PFAM	Domain	Count	Percentage (%)
PF05686.13	Glycosyl transferase family 90	6	0.02
PF02319.21	E2F/DP family winged-helix DNA-binding domain	2	0.02

d

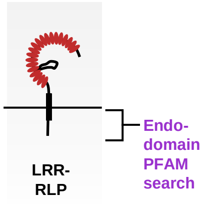

PFAM	Domain	Count	Percentage (%)
PF01582.21	TIR domain	7	0.03
PF07727.15	RNA-dependent DNA polymerase	7	0.03
PF13041.7	PPR repeat family	7	0.03

e

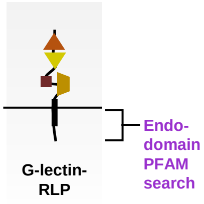

PFAM	Domain	Count	Percentage (%)
PF11883.9	DUF3403	31	1.56
PF01246.21	Ribosomal protein L24e	5	0.25
PF03030.17	Inorganic H+ pyrophosphatase	5	0.25

f

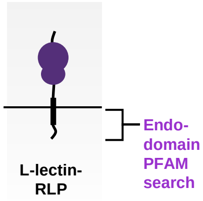

PFAM	Domain	Count	Percentage (%)
PF13561.7	Enoyl-(Acyl carrier protein) reductase	4	0.29
PF00106.26	Short chain dehydrogenase	3	0.22

g

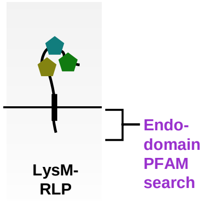

PFAM	Domain	Count	Percentage (%)
PF07714.18	Protein tyrosine and serine/threonine kinase	53	4.02
PF08392.13	FAE1/Type III polyketide synthase-like protein	21	1.59
PF00168.31	C2 doamin	8	0.61

h

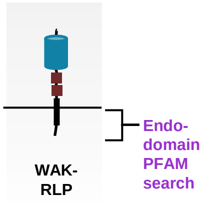

PFAM	Domain	Count	Percentage (%)
PF13639.7	Ring finger domain	538	13.94
PF07714.18	Protein tyrosine and serine/threonine kinase	5	0.13
PF14380.7	Wall-associated receptor kinase C-terminal	5	0.13

i

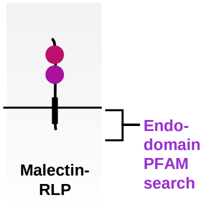

PFAM	Domain	Count	Percentage (%)
PF00076.23	RNA recognition motif	2	0.22
PF04646.13	DUF604	2	0.22

j

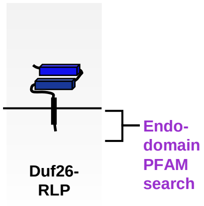

PFAM	Domain	Count	Percentage (%)
PF02358.17	Trehalose-phosphatase	12	0.32
PF00011.22	Hsp20/alpha crystallin family	2	0.05
PF14223.7	gag-polypeptide of LTR copia-type	2	0.05

Supplementary Data 1b. Endo-domain analysis of RLKs and RLPs. a Method of domain identification in the endo-domain of RLKs and RLPs. For details, please refer to methods. Enriched domain found in the endo-domain of **b** RLKs, **c** RLPs, **d** LRR-RLPs, **e** G-lectin-RLPs, **f** L-lectin-RLPs, **g** LysM-RLPs, **h** WAK-RLPs, **i** Malectin-RLPs, and **j** Duf26-RLP. Percentage (%) represents the proportion of proteins with the stated PFAM domain relative to the total number of input proteins.

a

Searched for all available PFAM HMMs in the protein (e-value threshold of 1e-10) → Kept the hit with the best score for each protein → Count number of hits per PFAM domains → Kept domains that were found in more than 1 or 1 out of 10,000 input sequences, filter out endo-domains that match any ecto-domains. → Final PFAM list

b

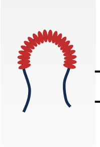

LRR-only (No TM/KD)

Associated domain PFAM search

PFAM	Domain	Count	Percentage (%)
PF13041.7	PPR repeat family	96267	1.45
PF00076.23	RNA recognition motif	54013	0.81
PF01535.21	PPR repeat	51291	0.77
PF00249.32	Myb-like DNA-binding domain	50464	0.76
PF00201.19	UDP-glucoronosyl and UDP-glucosyl transferase	43230	0.65
PF00067.23	Cytochrome P450	41635	0.63
PF00400.33	WD domain, G-beta repeat	41440	0.62
PF00141.24	Peroxidase	33654	0.51
PF02365.16	No apical meristem (NAM) protein	31772	0.48
PF00847.21	AP2 domain	31597	0.47

PFAM	Domain	Count	Percentage (%)
PF02458.16	Transferase family	28467	0.43
PF03171.21	2OG-Fe(II) oxygenase superfamily	27954	0.42
PF00071.23	Ras family	25065	0.38
PF03106.16	WRKY DNA -binding domain	24373	0.37
PF14111.7	Domain of unknown function (DUF4283)	23908	0.36
PF00481.22	Protein phosphatase 2C	22334	0.34
PF00657.23	GDSL-like Lipase/Acyl hydrolase	22055	0.33
PF00270.30	DEAD/DEAH box helicase	20013	0.3
PF00004.30	ATPase family associated with various cellular activities (AAA)	18537	0.28
PF02362.22	B3 DNA binding domain	17101	0.26

PFAM	Domain	Count	Percentage (%)
PF00319.19	SRF-type transcription factor	17003	0.26
PF03514.15	GRAS domain family	16866	0.25
PF00082.23	Subtilase family	16836	0.25
PF00153.28	Mitochondrial carrier protein	16164	0.24
PF00931.23	NB-ARC domain	16074	0.24
PF00190.23	Cupin	15937	0.24
PF00225.24	Kinesin motor domain	15212	0.23
PF14543.7	Xylanase inhibitor N-terminal	14418	0.22
PF00036.33	EF hand	14203	0.21
PF03195.15	Lateral organ boundaries (LOB) domain	14044	0.21

c

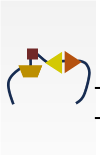

G-lectin-only (No TM/KD)

Associated domain PFAM search

PFAM	Domain	Count	Percentage (%)
PF00400.33	WD domain, G-beta repeat	14	0.27
PF00332.19	Glycosyl hydrolases family 17	12	0.23
PF05183.13	RNA dependent RNA polymerase	5	0.1

d

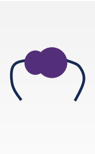

L-lectin-only (No TM/KD

Associated domain PFAM search

PFAM	Domain	Count	Percentage (%)
PF00515.29	Tetratricopeptide repeat	9	0.55
PF00332.19	Glycosyl hydrolases family 17	4	0.24
PF07719.18	Tetratricopeptide repeat	3	0.18

e

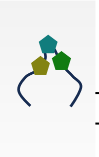

LysM-only (No TM/KD)

Associated domain PFAM search

PFAM	Domain	Count	Percentage (%)
PF10358.10	N-terminal C2 in EEIG1 and EHBP1 proteins	170	4.92
PF00112.24	Papain family cysteine protease	81	2.34
PF01551.23	Peptidase family M23	28	0.81

f

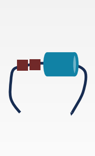

WAK-only (No TM/KD)

Associated domain PFAM search

PFAM	Domain	Count	Percentage (%)
PF14380.7	Wall-associated receptor kinase C-terminal	758	11.2
PF08488.12	Wall-associated kinase	44	0.65
PF01457.17	Leishmanolysin	28	0.41

g

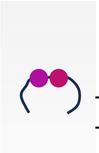

Malectin-only (No TM/KD)

Associated domain PFAM search

PFAM	Domain	Count	Percentage (%)
PF00225.24	Kinesin motor domain	524	22.13
PF00514.24	Armadillo/beta-catenin-like repeat	6	0.25
PF01833.25	IPT/TIG domain	4	0.17

h

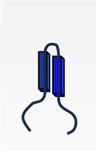

Duf26-only (No TM/KD)

Associated domain PFAM search

PFAM	Domain	Count	Percentage (%)
PF04720.13	PDDEXK-like family of unknown function	17	0.32
PF02358.17	Trehalose-phosphatase	12	0.23
PF01627.24	Hpt domain	3	0.06

Supplementary Data 1c. Ectodomain-Associated domain analysis. a Method of associated-domain identification in the ectodomain-only proteins. For details, please refer to methods. Enriched domain found in the ectodomain-only proteins of **b** LRR, **c** G-lectin, **d** L-lectin, **e** LysM, **f** WAK, **g** Malectin, and **h** Duf26. Percentage (%) represents the proportion of proteins with the stated PFAM domain relative to the total number of input proteins.
