# Supplementary material for: Evolutionary trajectory of pattern recognition receptors in plants
Source: Nat Commun. 2024 Feb 1;15:308. doi: 10.1038/s41467-023-44408-3 (PMC10834447; doi:10.1038/s41467-023-44408-3)
Supplement: Supplementary file 7 — Reporting Summary [file 41467_2023_44408_MOESM7_ESM.pdf]

nature portfolio

Corresponding author(s): Ken Shirasu

Last updated by author(s): Dec 11, 2023

Reporting Summary

Nature Portfolio wishes to improve the reproducibility of the work that we publish. This form provides structure for consistency and transparency in reporting. For further information on Nature Portfolio policies, see our Editorial Policies and the Editorial Policy Checklist.

Statistics

For all statistical analyses, confirm that the following items are present in the figure legend, table legend, main text, or Methods section.

n/a	Confirmed	
☐	☒	The exact sample size (*n*) for each experimental group/condition, given as a discrete number and unit of measurement
☐	☒	A statement on whether measurements were taken from distinct samples or whether the same sample was measured repeatedly
☐	☒	The statistical test(s) used AND whether they are one- or two-sided<br>*Only common tests should be described solely by name; describe more complex techniques in the Methods section.*
☒	☐	A description of all covariates tested
☐	☒	A description of any assumptions or corrections, such as tests of normality and adjustment for multiple comparisons
☐	☒	A full description of the statistical parameters including central tendency (e.g. means) or other basic estimates (e.g. regression coefficient) AND variation (e.g. standard deviation) or associated estimates of uncertainty (e.g. confidence intervals)
☐	☒	For null hypothesis testing, the test statistic (e.g. *F*, *t*, *r*) with confidence intervals, effect sizes, degrees of freedom and *P* value noted<br>*Give P values as exact values whenever suitable.*
☒	☐	For Bayesian analysis, information on the choice of priors and Markov chain Monte Carlo settings
☒	☐	For hierarchical and complex designs, identification of the appropriate level for tests and full reporting of outcomes
☐	☒	Estimates of effect sizes (e.g. Cohen's *d*, Pearson's *r*), indicating how they were calculated

Our web collection on statistics for biologists contains articles on many of the points above.

Software and code

Policy information about availability of computer code

Data collection	Proteomes of 350 species used in this study were downloaded from either NCBI, Phyozome13, ensemblplants, JGI, Fernbase, Penium Genome Database or directly from the publications. A complete list of the proteomes and associated data used in this study are provided in Supplementary Table 1 of our previous publication (DOI: 10.1038/s41477-022-01260-5). Sources, publication identifiers (DOIs), contact information and other relevant information regarding these publicly available proteomes are as well.
Data analysis	All analyses were done as described with publicly available tools (hmmer, tmhmm ,usearch, diamond, FastTree, FAMSA, gotree, R).

For manuscripts utilizing custom algorithms or software that are central to the research but not yet described in published literature, software must be made available to editors and reviewers. We strongly encourage code deposition in a community repository (e.g. GitHub). See the Nature Portfolio guidelines for submitting code & software for further information.

Data

Policy information about availability of data

All manuscripts must include a data availability statement. This statement should provide the following information, where applicable:

- Accession codes, unique identifiers, or web links for publicly available datasets
- A description of any restrictions on data availability
- For clinical datasets or third party data, please ensure that the statement adheres to our policy

All data generated or analysed during this study are included in the article or supplementary materials. Proteomes of 350 species used in this study are downloaded from either NCBI, Phyozome13, ensemblplants, JGI, Fernbase, Penium Genome Database, or directly from the publications. A complete list of the proteomes and

associated data used in this study is published4. Sequences, alignment and tree files of the identified receptors and downstream signaling components, together with relevant phylogenetic analyses are available on Zenodo (https://zenodo.org/records/10059978). Source data are provided with this paper.

Research involving human participants, their data, or biological material

Policy information about studies with human participants or human data. See also policy information about sex, gender (identity/presentation), and sexual orientation and race, ethnicity and racism.

Reporting on sex and gender	N/A
Reporting on race, ethnicity, or other socially relevant groupings	N/A
Population characteristics	N/A
Recruitment	N/A
Ethics oversight	N/A

Note that full information on the approval of the study protocol must also be provided in the manuscript.

Field-specific reporting

Please select the one below that is the best fit for your research. If you are not sure, read the appropriate sections before making your selection.

- [x] Life sciences
- [ ] Behavioural & social sciences
- [ ] Ecological, evolutionary & environmental sciences

For a reference copy of the document with all sections, see nature.com/documents/nr-reporting-summary-flat.pdf

Life sciences study design

All studies must disclose on these points even when the disclosure is negative.

Sample size	Sample sizes for each experiment are described in the figure legends or methods. Samples sizes were determined based on experimental trials and previous publications (https://doi.org/10.1038/s41586-021-03315-7).
Data exclusions	No data were excluded from the analyses.
Replication	All experiments were independently replicated at least two times. All experimental results can be successfully reproduced.
Randomization	All samples were randomly arranged into experimental groups.
Blinding	All experiments were performed without prior knowledge of the outcomes. Investigators were not blinded during data collection or analyses.

Reporting for specific materials, systems and methods

We require information from authors about some types of materials, experimental systems and methods used in many studies. Here, indicate whether each material, system or method listed is relevant to your study. If you are not sure if a list item applies to your research, read the appropriate section before selecting a response.

Materials & experimental systems

n/a	Involved in the study	
[ ]	[x]	Antibodies
[x]	[ ]	Eukaryotic cell lines
[x]	[ ]	Palaeontology and archaeology
[x]	[ ]	Animals and other organisms
[x]	[ ]	Clinical data
[x]	[ ]	Dual use research of concern
[ ]	[x]	Plants

Methods

n/a	Involved in the study	
[x]	[ ]	ChIP-seq
[x]	[ ]	Flow cytometry
[x]	[ ]	MRI-based neuroimaging

Antibodies

Antibodies used	Phospho-p44/42 MAPK (Erk1/2) (Thr202/Tyr204) (D13.14.4E) XP® Rabbit mAb (CST, #4370); Anti-HA high affinity antibody 3F10 (Roche, 11867431001); Anti-GFP antibody ab290 (abcam, ab290); Monoclonal ANTI-FLAG® M2-Peroxidase (HRP) antibody (MERCK, A8592); Rabbit IgG HRP Linked F(ab')2 (Cytiva, NA9340)

Validation	Antibodies used in this study are commercially available and have been validated for specificity.

Dual use research of concern

Policy information about dual use research of concern

Hazards

Could the accidental, deliberate or reckless misuse of agents or technologies generated in the work, or the application of information presented in the manuscript, pose a threat to:

No	Yes	
☒	☐	Public health
☒	☐	National security
☒	☐	Crops and/or livestock
☒	☐	Ecosystems
☒	☐	Any other significant area

Experiments of concern

Does the work involve any of these experiments of concern:

No	Yes	
☒	☐	Demonstrate how to render a vaccine ineffective
☒	☐	Confer resistance to therapeutically useful antibiotics or antiviral agents
☒	☐	Enhance the virulence of a pathogen or render a nonpathogen virulent
☒	☐	Increase transmissibility of a pathogen
☒	☐	Alter the host range of a pathogen
☒	☐	Enable evasion of diagnostic/detection modalities
☒	☐	Enable the weaponization of a biological agent or toxin
☒	☐	Any other potentially harmful combination of experiments and agents

Plants

Seed stocks	N/A
Novel plant genotypes	No novel plant genotypes were produced.
Authentication	N/A
