# Supplementary material for: Evolutionary trajectory of pattern recognition receptors in plants
Source: Nat Commun. 2024 Feb 1;15:308. doi: 10.1038/s41467-023-44408-3 (PMC10834447; doi:10.1038/s41467-023-44408-3)

Uncropped gel

Figure 5g

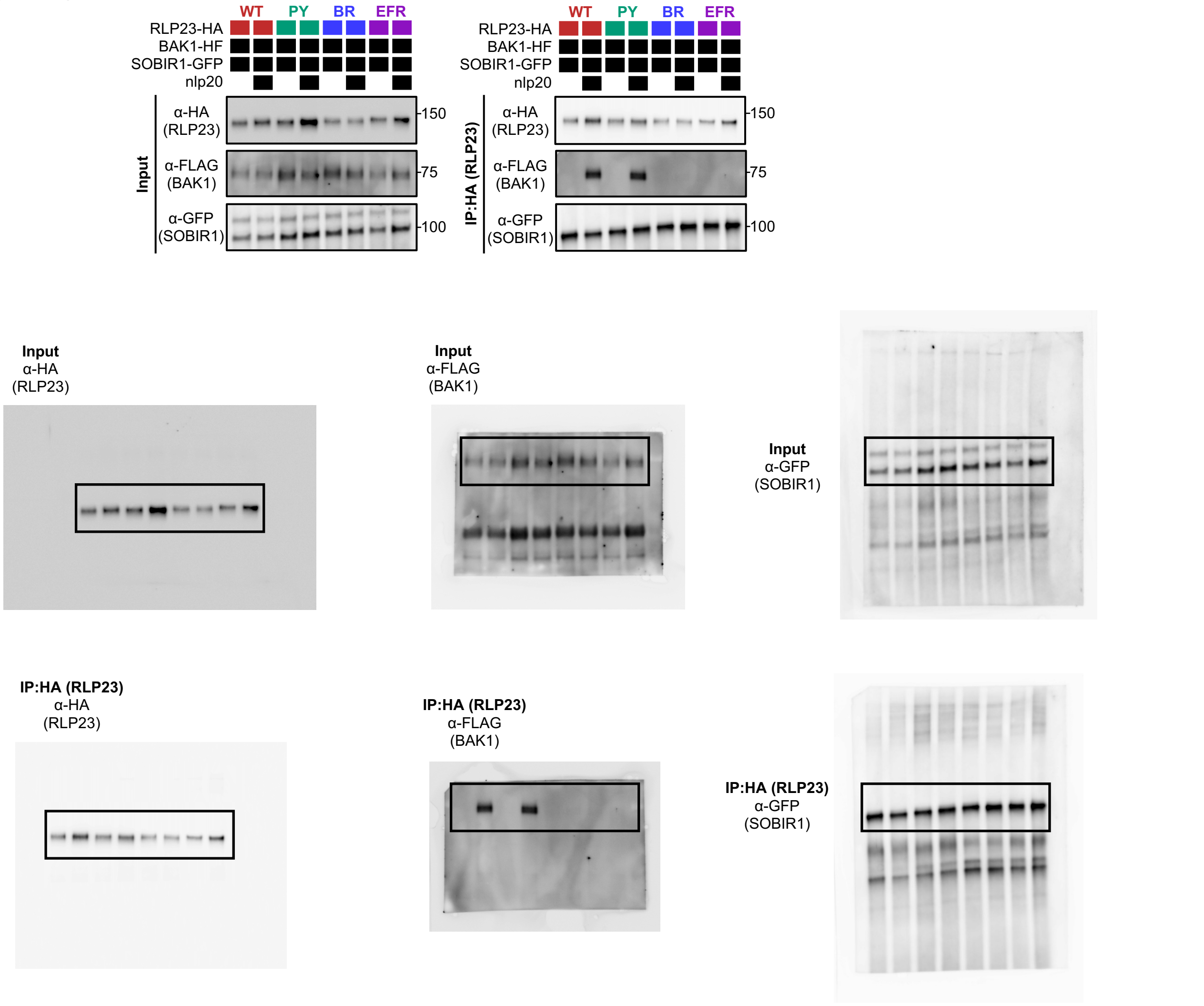

Figure 5h

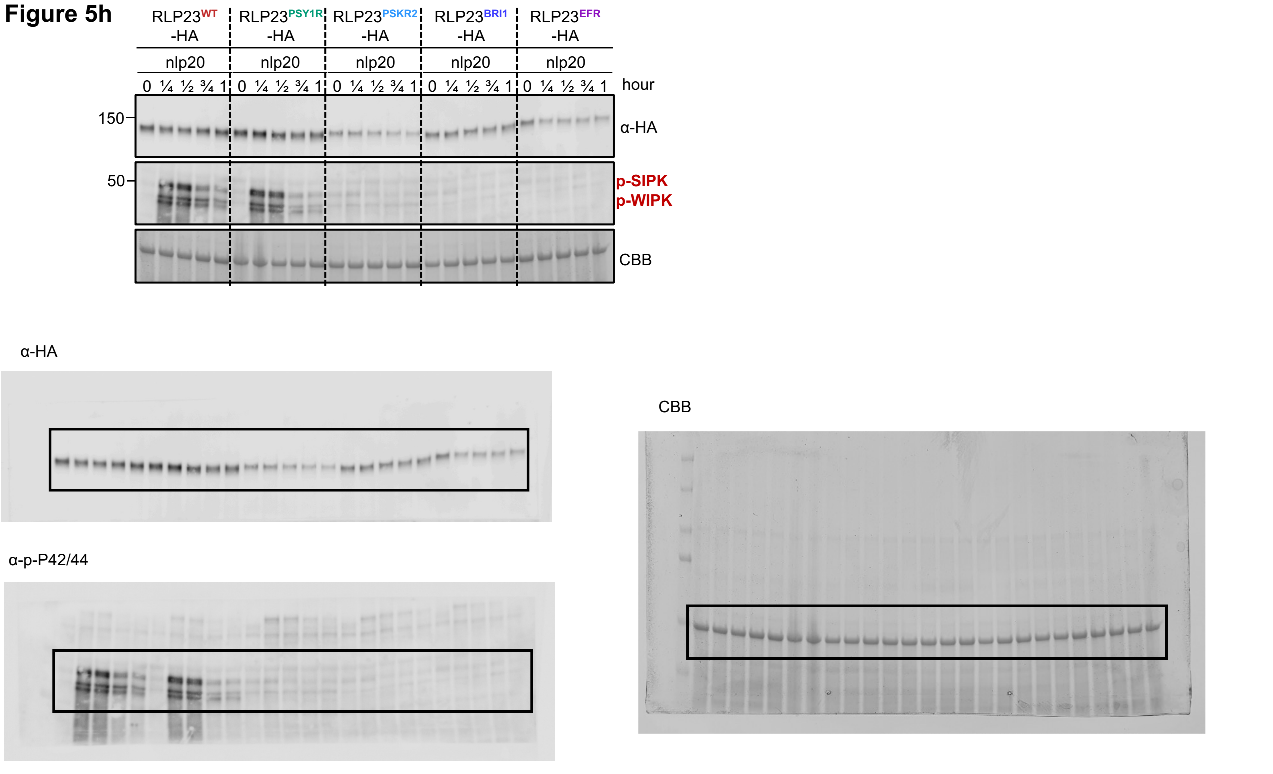

Uncropped gel

Figure 5j

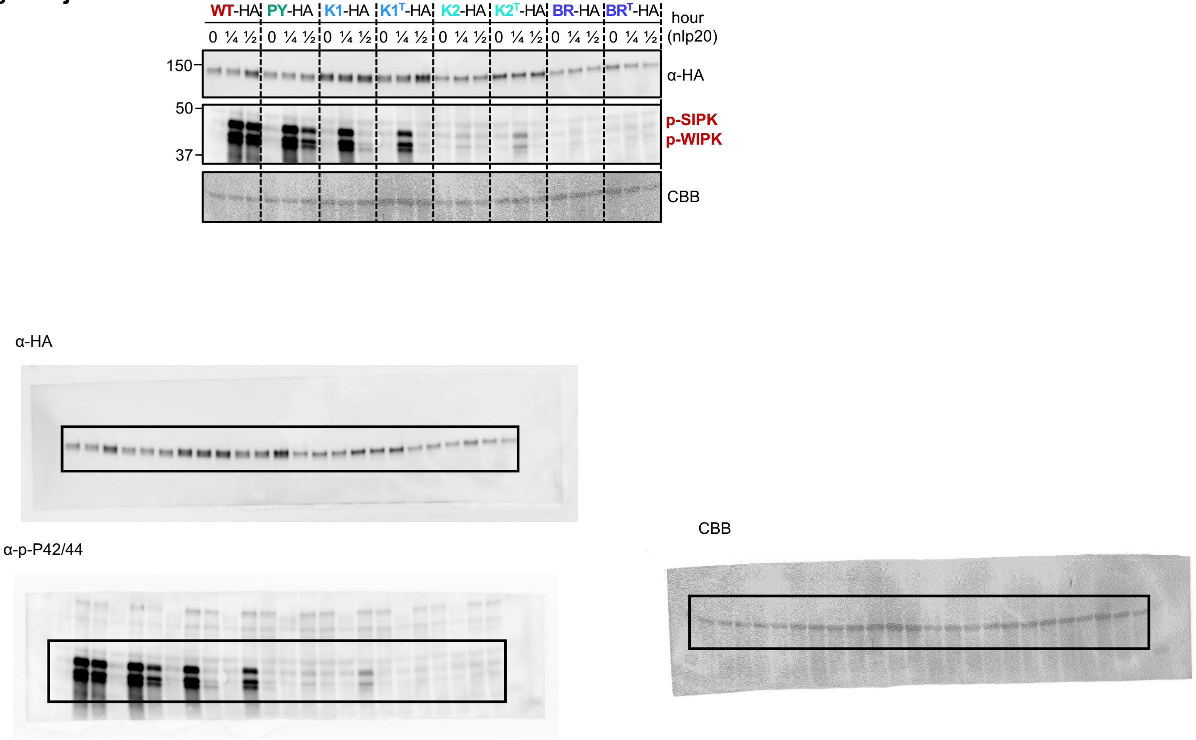

Figure 5l

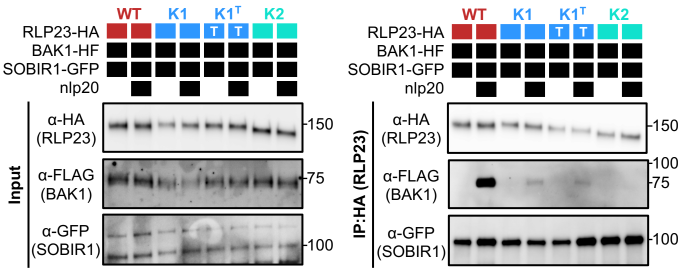

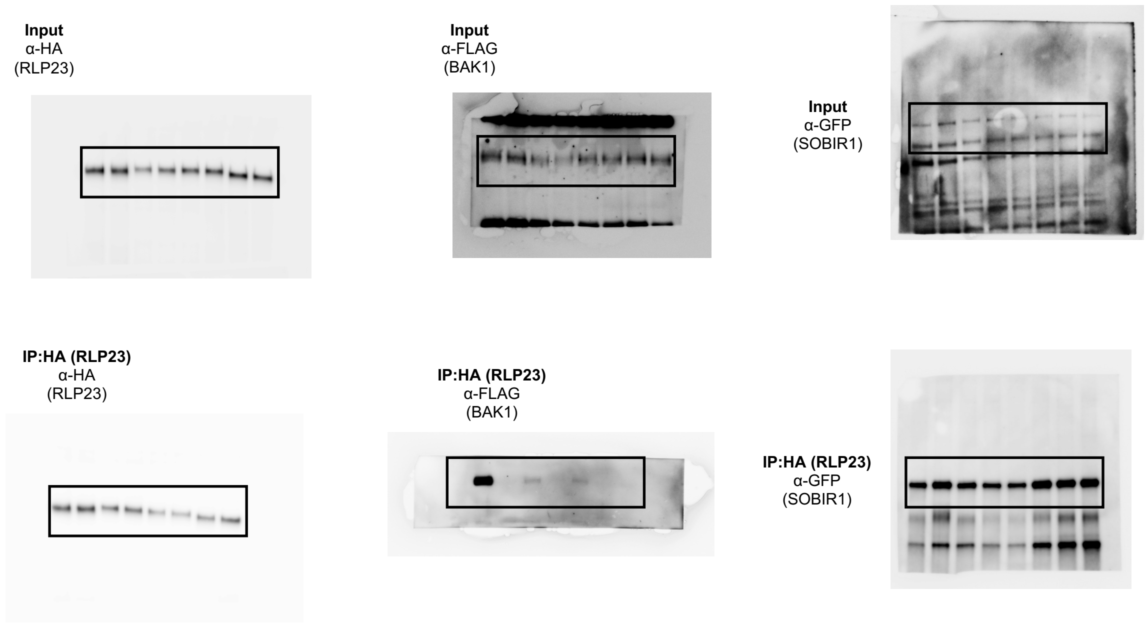

Uncropped gel

Supp Figure 18

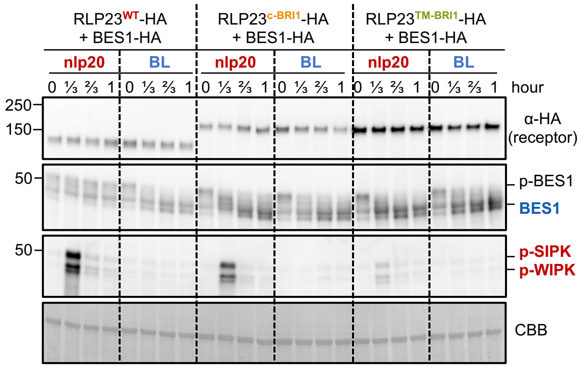

α-HA (receptor)

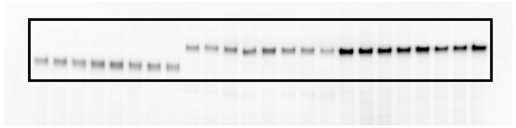

α-HA (BES1)

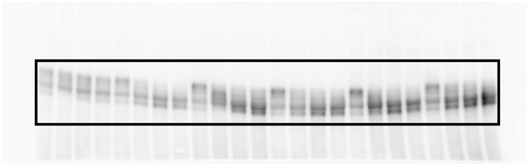

α-p-P42/44

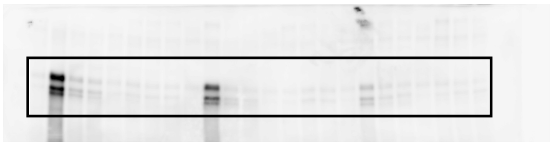

CBB

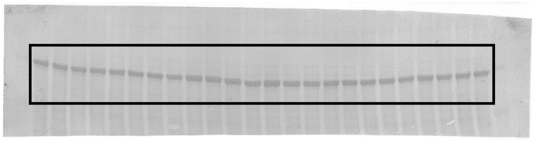

Uncropped gel

Figure 8d

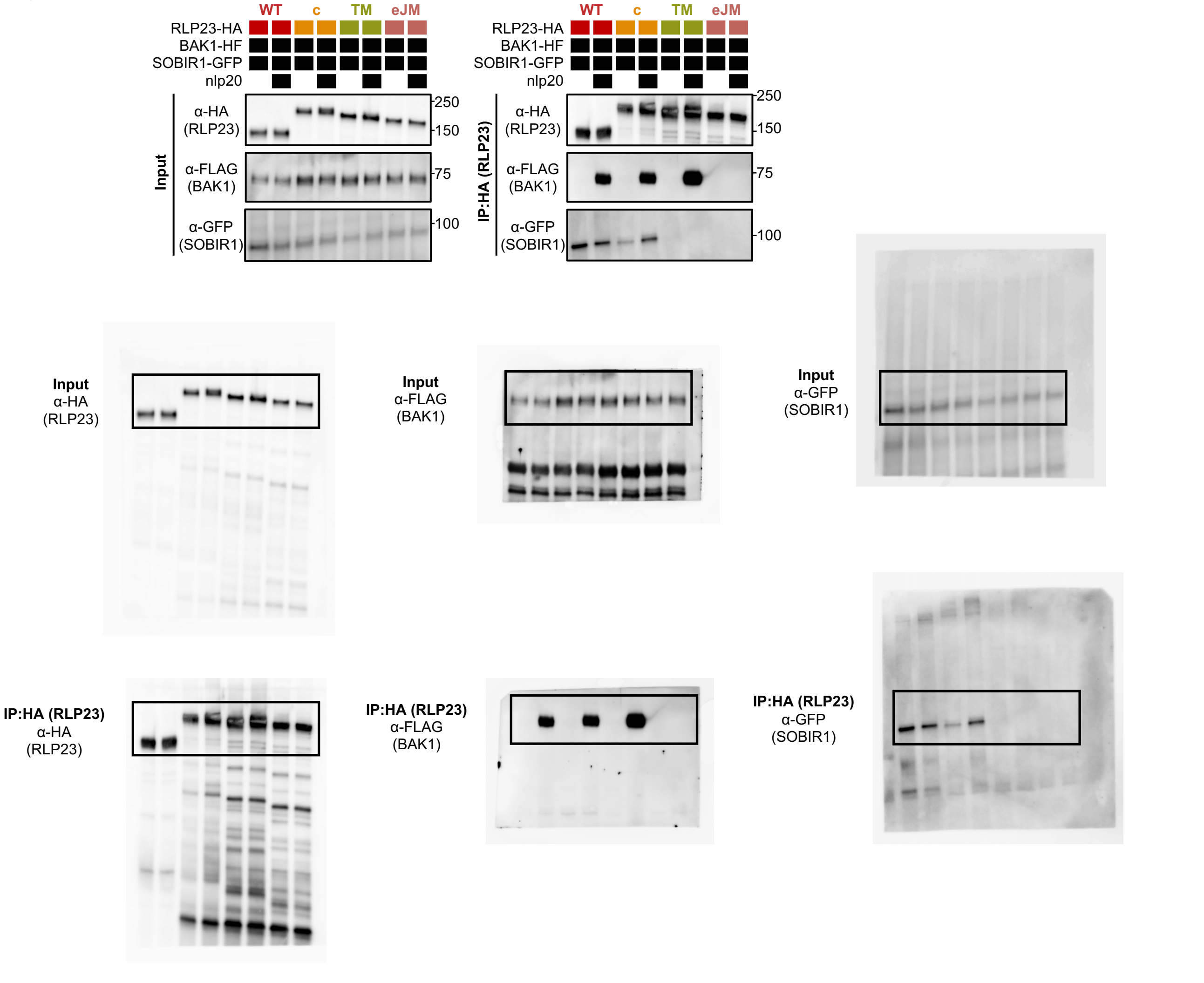

Figure 8e

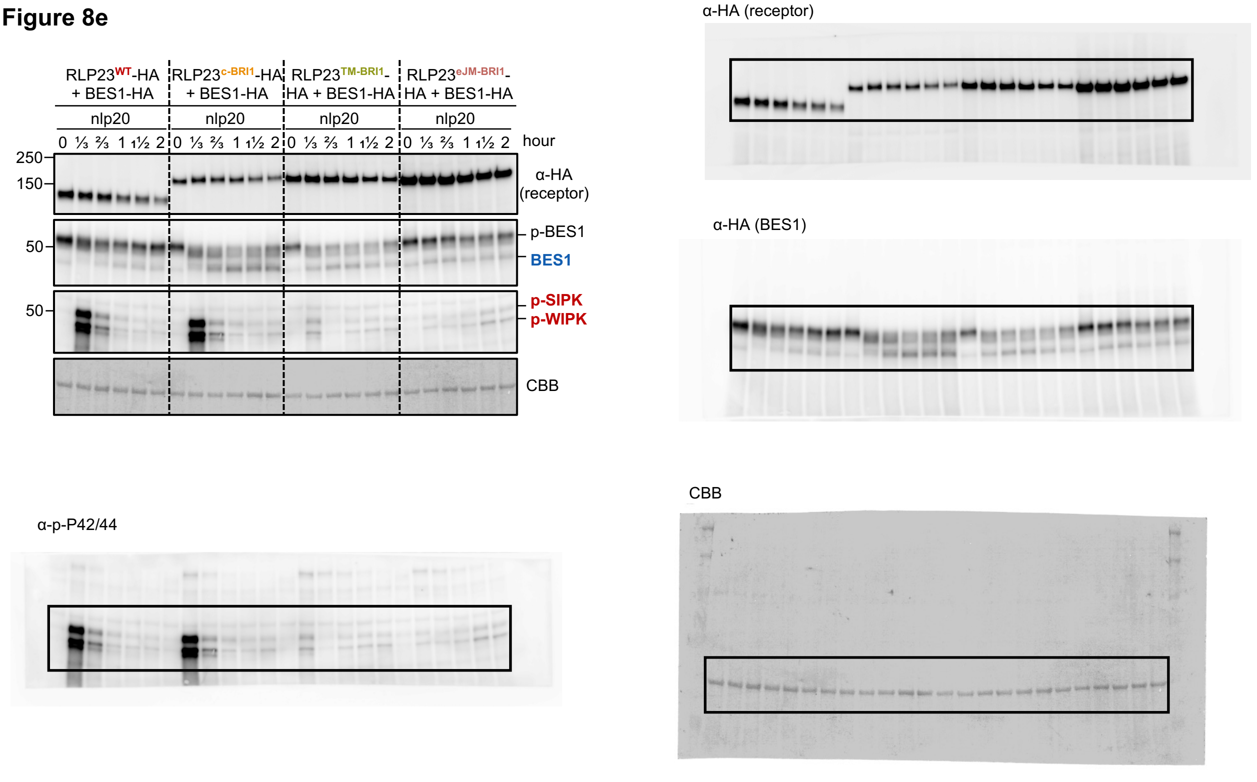

Uncropped gel

Figure 8g

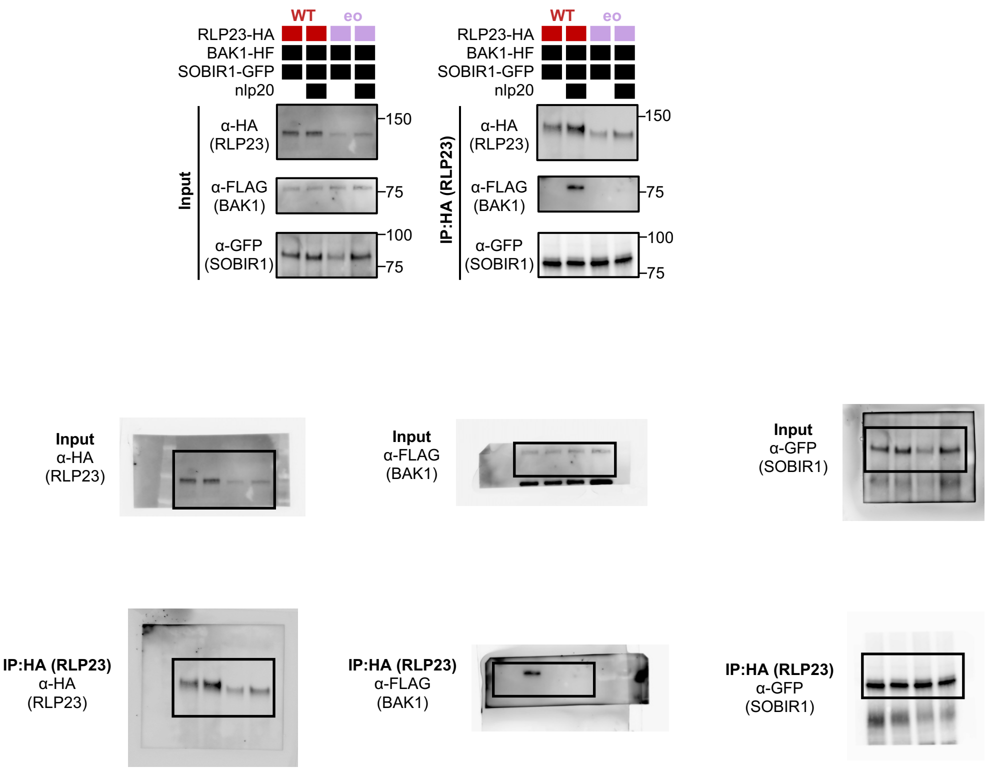

Figure 8h

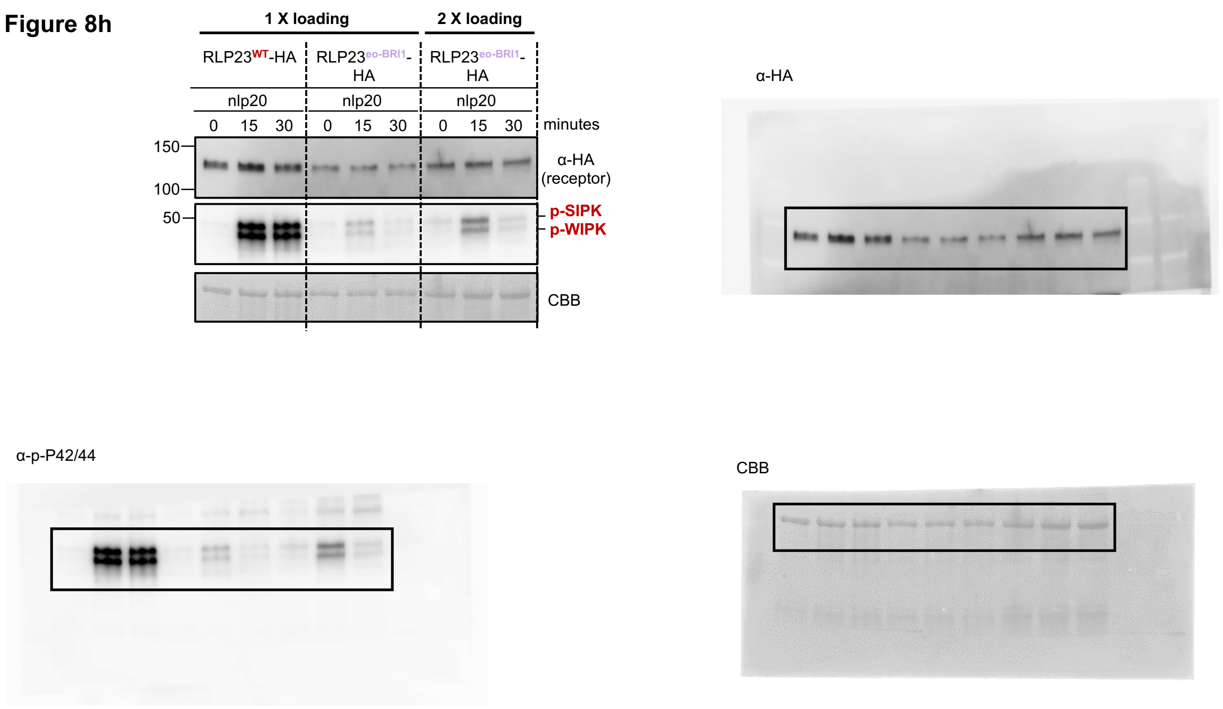

Supplement: Supplementary file 8 — Source data file [file 41467_2023_44408_MOESM8_ESM.zip › Source data (uncropped gel).pdf]
